# Prolonged exposure to traffic-related particulate matter and gaseous pollutants implicate distinct molecular mechanisms of lung injury in rats

**DOI:** 10.1186/s12989-021-00417-y

**Published:** 2021-06-25

**Authors:** Yu-Teng Jheng, Denise Utami Putri, Hsiao-Chi Chuang, Kang-Yun Lee, Hsiu-Chu Chou, San-Yuan Wang, Chia-Li Han

**Affiliations:** 1 Master Program in Clinical Pharmacogenomics and Pharmacoproteomics, College of Pharmacy, Taipei Medical University, Mailing address: 250 Wuxing St, Taipei, 11031 Taiwan; 2 International Ph.D. Program in Medicine, College of Medicine, Taipei Medical University, Taipei, Taiwan; 3 Pulmonary Research Center, Division of Pulmonary Medicine, Department of Internal Medicine, Wan Fang Hospital, Taipei Medical University, Taipei, Taiwan; 4 School of Respiratory Therapy, College of Medicine, Taipei Medical University, Taipei, Taiwan; 5 Division of Pulmonary Medicine, Department of Internal Medicine, School of Medicine, College of Medicine, Taipei Medical University, Taipei, Taiwan; 6 Division of Pulmonary Medicine, Department of Internal Medicine, Shuang Ho Hospital, Taipei Medical University, New Taipei City, Taiwan; 7 Department of Anatomy and Cell Biology, School of Medicine, College of Medicine, Taipei Medical University, Taipei, Taiwan

**Keywords:** Traffic-related air pollution, Particulate matter, Gaseous pollutant, Lung injury, Molecular mechanism, Proteomics

## Abstract

**Background:**

Exposure to air pollution exerts direct effects on respiratory organs; however, molecular alterations underlying air pollution-induced pulmonary injury remain unclear. In this study, we investigated the effect of air pollution on the lung tissues of Sprague-Dawley rats with whole-body exposure to traffic-related PM_1_ (particulate matter < 1 μm in aerodynamic diameter) pollutants and compared it with that in rats exposed to high-efficiency particulate air–filtered gaseous pollutants and clean air controls for 3 and 6 months. Lung function and histological examinations were performed along with quantitative proteomics analysis and functional validation.

**Results:**

Rats in the 6-month PM_1_-exposed group exhibited a significant decline in lung function, as determined by decreased FEF_25–75%_ and FEV_20_/FVC; however, histological analysis revealed earlier lung damage, as evidenced by increased congestion and macrophage infiltration in 3-month PM_1_-exposed rat lungs. The lung tissue proteomics analysis identified 2673 proteins that highlighted the differential dysregulation of proteins involved in oxidative stress, cellular metabolism, calcium signalling, inflammatory responses, and actin dynamics under exposures to PM_1_ and gaseous pollutants. The presence of PM_1_ specifically enhanced oxidative stress and inflammatory reactions under subchronic exposure to traffic-related PM_1_ and suppressed glucose metabolism and actin cytoskeleton signalling. These factors might lead to repair failure and thus to lung function decline after chronic exposure to traffic-related PM_1_. A detailed pathogenic mechanism was proposed to depict temporal and dynamic molecular regulations associated with PM_1_- and gaseous pollutants-induced lung injury.

**Conclusion:**

This study explored several potential molecular features associated with early lung damage in response to traffic-related air pollution, which might be used to screen individuals more susceptible to air pollution.

**Supplementary Information:**

The online version contains supplementary material available at 10.1186/s12989-021-00417-y.

## Background

Ambient air pollution substantially contributes to disease burden and mortality globally, with at least 4.2 million deaths reported in 2016 [1]. Among various pollutants present in ambient air, exposure to particulate matter (PM), particularly those in fine (< 2.5 μm in aerodynamic diameter; PM_2.5_) and ultrafine ranges (< 0.1 μm; PM_0.1_), is considered a key risk factor for many adverse health consequences; prominently reported are acute or chronic respiratory complaints, which are caused by direct deterioration of respiratory organs upon inhalation of air pollutants [2, 3]. Both short- and long-term exposure to fine PM significantly reduce lung function through increased pulmonary oxidative stress and persistent inflammation [4–7]. Multiple signalling pathways involved in the transcriptional and/or translational activations of AMP-activated protein kinase (AMPK)/signal transducer and activator of transcription (STAT)-1 [8], epithelial growth factor receptor (EGFR)/mitogen-activated protein kinase (MAPK)/nuclear factor κB (NF-κB) [9], and transforming growth factor (TGF)-β/Smad [10] that promote the release of proinflammatory cytokines have been proposed as potential pathogenic mechanisms of PM-induced pulmonary toxicity in lung epithelial cells or rodent models. However, the detailed pathogenesis remains to be fully investigated.

Mass spectrometry (MS)-based proteomics analysis, which enables unbiased identification and quantification of thousands of proteins, has been applied to examine aberrant molecular profiles upon PM-induced damage to primary skin keratinocytes [11], trophoblast cells [12], lung epithelial cells [13], rat lungs [14], and rat brains [15]. Several dysregulated proteins involved in mitochondrial dysfunction, energy metabolism, and endoplasmic reticulum stress have been found to be potential biomarkers for PM-induced organ damage. To exploit the effect of PM on organ injury, we previously established a rat model with a whole-body exposure system and reported central neurotoxicity induced by subchronic or chronic exposure to traffic-related PM_1_ [16]. In the present study, we investigated the effects of traffic-related air pollution in the lungs by using the same rat model. In particular, we elaborated the lung function and pathological changes upon subchronic or chronic exposure to traffic-related air pollution as well as systematically elucidated dysregulated molecules and signalling pathways by quantitative proteomics analysis. Furthermore, we constructed the detailed molecular mechanisms underlying traffic-related air pollution-induced lung injury.

## Results

### Lung function and histological examination

**Fig. 1:**
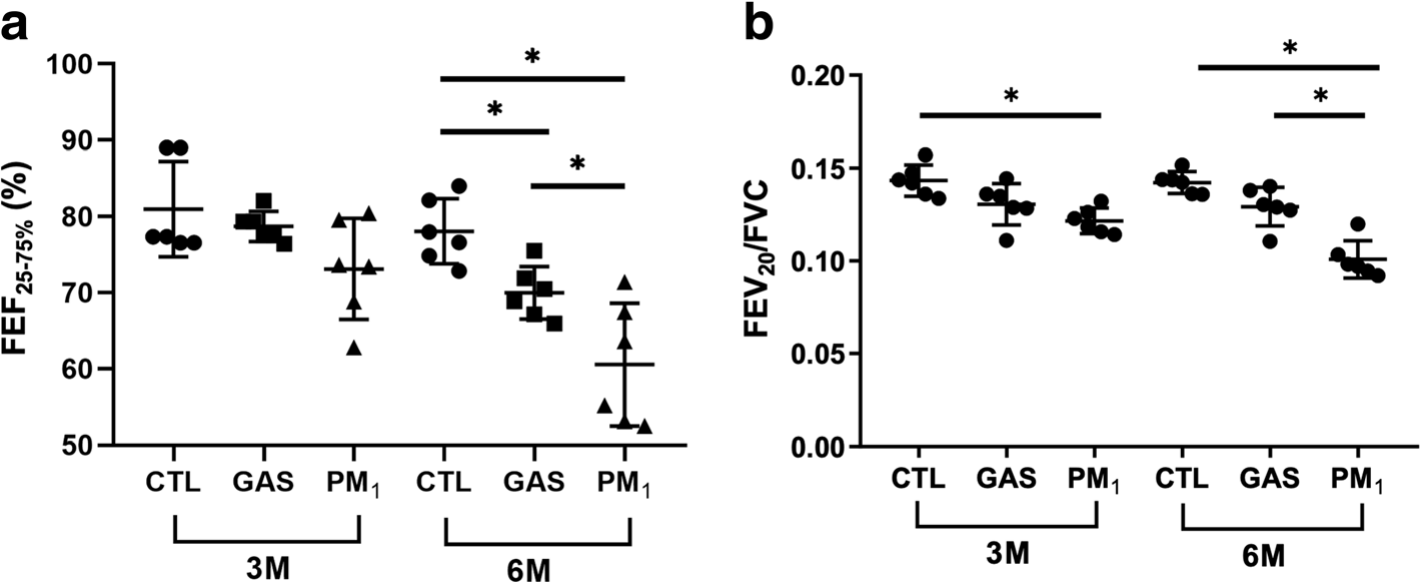
Lung function examination. Forced expiratory flow at 25–75% of forced vital capacity (FEF_25–75%_) and the ratio of forced expiratory volume at 20 ms and forced vital capacity (FEV_20_/FVC) were measured for each rat with at least three acceptable measurements. Significant reductions in FEF_25–75%_ and FEV_20_/FVC were observed in rats from the 6 M-PM_1_ group; * *p* < 0.05

**Fig. 2:**
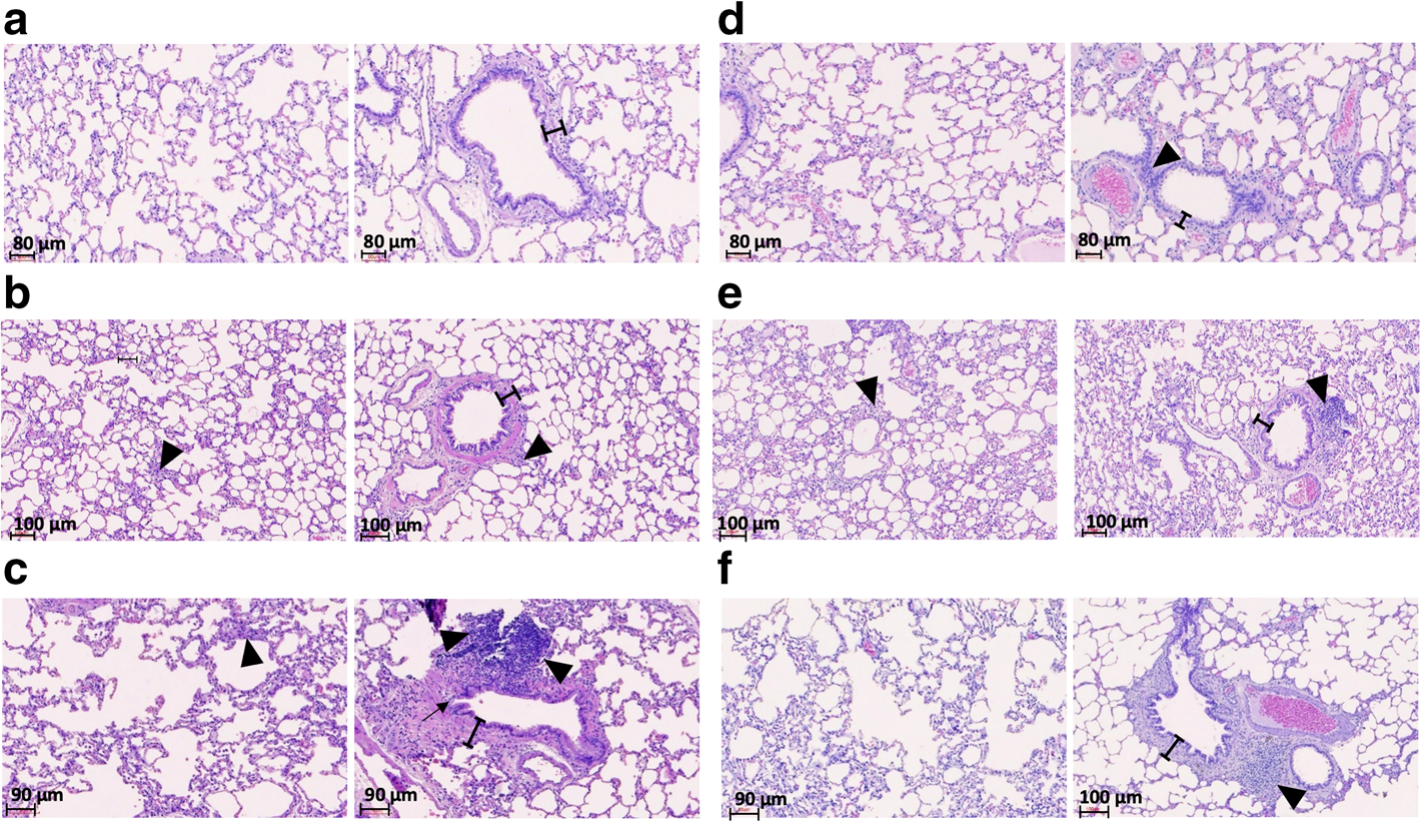
Histological images of rat lung tissues. **a**, **b**, and **c** are representative images of the 3 M-CTL, 3 M-GAS, and 3 M-PM_1_ groups, whereas **d**, **e**, and **f** are representative images of the 6 M-CTL, 6 M-GAS, and 6 M-PM_1_ groups, respectively. The images show the alveoli (left panel) and airway (right panel) tissues. Bars in the airway figures indicate the thickness of the airway wall. Black arrowheads point to the infiltration of immune cells within the alveolar and bronchial walls. The black arrow in **c** indicates damage within the bronchial wall

We examined the lung function of each rat by measuring the forced expiratory flow at 25–75% of the pulmonary volume (FEF_25–75%_) and the ratio of forced expiratory volume at 20 ms and forced vital capacity (FEV_20_/FVC). As depicted in Fig. 1, no significant differences existed in FEF_25–75%_ among rats in the 3-month (3 M) exposure groups, while FEV_20_/FVC was significantly lower in PM_1_ group compared to the control. After 6 months of exposure, rats in the PM_1_ group exhibited significant decreases in both FEF_25–75%_ and FEV_20_/FVC compared with rats treated with high-efficiency particulate air (HEPA)-filtered gaseous pollutants (GAS) and clean air controls (CTL). In addition, rats in the GAS group exhibited decreased FEF_25–75%_ compared with those in the CTL group. Subsequently, we performed lung histological analysis to evaluate the degree of lung injury based on the presence and severity of congestion, haemorrhage, immune cell infiltration, and alveolar wall thickness. The 3 M-PM_1_-exposed lungs already exhibited higher levels of congestion and macrophage infiltration, indicating significant lung injury in PM_1_ rats compared with GAS and CTL rats. Similar results were observed after chronic exposure to PM_1_ (Table 1). Figure 2 presents histological images demonstrating the increased thickness of airway walls and the disruption of alveolar and airway integrity, along with abundant immune cell accumulation within the peribronchial area in both 3 M and 6-month (6 M) PM_1_-exposed lungs. However, no significant difference in lung injuries was observed between rats under 3 M and 6 M exposures.

**Table 1:** Lung injury score

Time exposure		Congestion		Hemorrhage		Thickness		Infiltration of immune cells		Lung injury score	
		Median	Range	Median	Range	Median	Range	Median	Range	Total	Range
**3 M**	**CTL**	1	(1–2)	1	(1–2)	1	(1–1)	1	(1–2)	5	(4–6)
	**GAS**	1	(0–1)	1	(1–2)	1	(1–1)	1	(0–1)	4^a^	(2–5)
	**PM1**	2^ab^	(1–2)	2	(1–3)	1	(1–2)	3^ab^	(1–3)	7.5^ab^	(5–9)
**6 M**	**CTL**	1	(0–1)	1	(1–2)	1	(1–2)	1	(0–2)	4	(2–6)
	**GAS**	1	(0–1)	1.5	(1–2)	1	(1–1)	1	(0–1)	4	(2–5)
	**PM1**	1^e^	(1–2)	2	(1–3)	1	(1–2)	2^cd^	(1–2)	6^de^	(4–8)

^a^vs 3 M-CTL, *p* < 0.05; ^b^vs 3 M-GAS, *p* < 0.05; ^c^vs 3 M-PM1, *p* < 0.05; ^d^vs 6 M-CTL, *p* < 0.05; ^e^vs 6 M-GAS, *p* < 0.05; Mann-Whitney Test

### Quantitative proteomics analysis of rat lungs

**Fig. 3:**
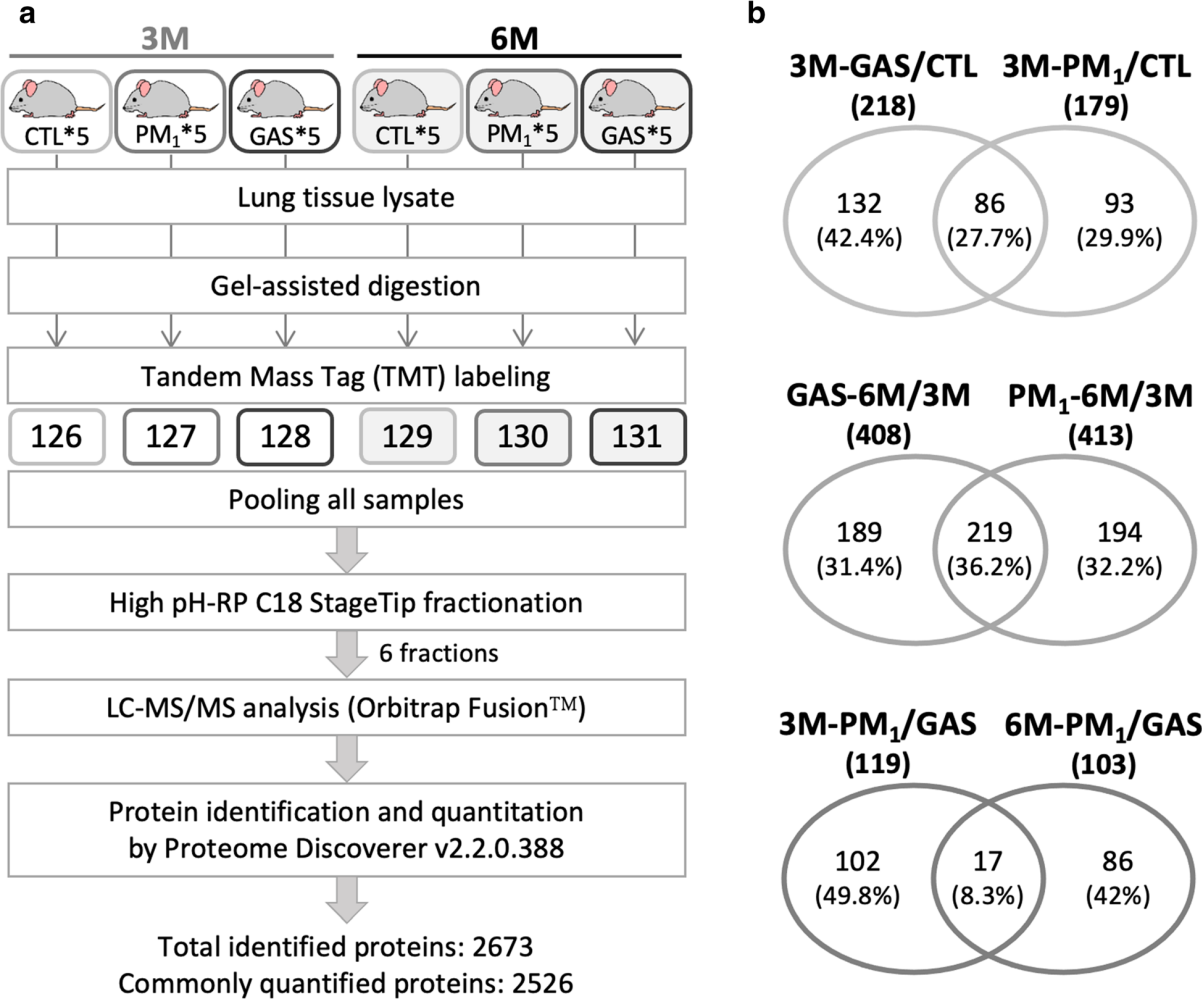
Workflow and DEPs in lung tissue proteomics analysis. **a** Experimental workflow for lung tissue proteomics analysis. The lung tissues from five rats in individual groups were lysed, digested, and pooled to generate six pooled peptide samples. Each pooled sample was labelled with one of the TMTs separately. The labelled peptides were then combined for reverse phase StageTip fractionation, followed by LC-MS/MS analysis and database searching for protein identification and quantitation. A total of 2673 proteins were identified, of which 2526 proteins were quantified. **b** The overlapping of DEPs in the 3 M-GAS and 3 M-PM_1_ groups, progressive GAS and PM_1_ exposures, and particle-specific DEPs under 3- and 6-month exposure

To elucidate molecular mechanisms underlying traffic-related air pollution (TRAP)-induced lung injury, we applied tandem mass tag (TMT)-based quantitative proteomics analysis to lung tissues obtained from the six exposure groups (Fig. 3A). A total of 2673 proteins were identified with a high confidence (*p* < 0.05, false discovery rate [FDR] < 1%), of which 2562 proteins were quantified (Fig. 3A). Differentially expressed proteins (DEPs) were identified by a log2 ratio higher than 0.38 or lower than − 0.38 (i.e., a 30% change in the expression level) in two comparisons. We arranged our analyses in three directions: (1) First, we observed the subchronic effect of TRAP by comparing protein expressions in the 3 M GAS and PM_1_ exposure groups with those in the CTL group; 218 and 179 DEPs were generated in the 3 M-GAS and 3 M-PM_1_ groups, respectively. (2) Second, proteins associated with progressive lung injury from exposure to GAS and PM_1_ were studied by comparing protein expressions between 6 M and 3 M exposures in the GAS and PM_1_ groups. A total of 408 and 413 progression-associated DEPs were identified in the GAS and PM_1_ groups, respectively. (3) Third, we elucidated particle-specific regulations by comparing the PM_1_ group with the GAS group under subchronic (3 M) and chronic (6 M) exposures, which resulted in 119 and 103 DEPs, respectively (Fig. 3B). The DEPs are listed in Additional files 1, 2 and 3: Table S1–3, and they were further analysed using Gene Ontology (GO) and Ingenuity Pathway Analysis (IPA) to delineate dysregulated cellular functions and pathways in the lungs.

### Dysregulated cellular functions and pathways in rat lungs under subchronic and chronic exposure to GAS and PM_1_ pollutants

**Fig. 4:**
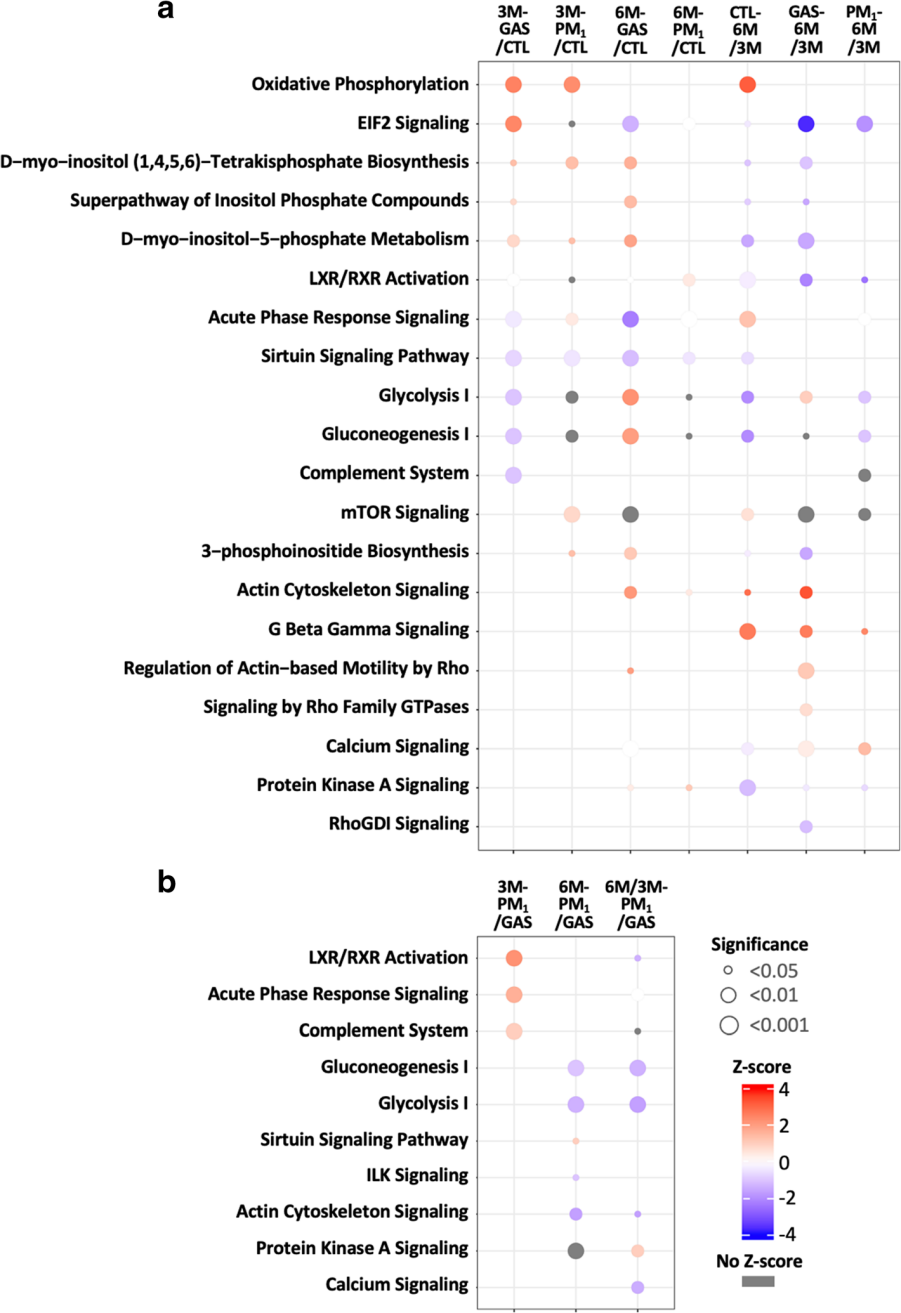
Enriched pathways associated with subchronic and chronic exposures to GAS and PM_1_. **a** The enriched pathways associated with subchronic (3-month, the two left columns), chronic (6-month, the two middle columns) to GAS and PM_1_ as well as progressive exposure to GAS, PM_1_ and CTL (the three right columns). **b** The enriched pathways specifically regulated by fine particles. Pathway enrichment analysis was performed using IPA. The size of the circle represents the *p* value for enrichment analysis. The colour of the circle indicates activation z-scores (red for upregulation and blue for downregulation)

As presented in Additional file 4: Fig. S1, 3 M exposure to traffic-related gaseous and PM_1_ pollutants induced alterations in proteins involved in metabolism- and acute phase response-related biological processes. Upregulation of the arginine metabolic process and fatty acid beta-oxidation as well as downregulation of endopeptidase inhibitor activity and negative regulation of the mRNA metabolic process were enriched in the 3 M-GAS group (Additional file 4: Fig. S1A). Similarly, subchronic exposure to PM_1_ upregulated triglyceride catabolism, reactive oxygen species (ROS) metabolism, long-chain fatty acid metabolism, and myelination and downregulated the glycolytic process in lung tissues (Additional file 4: Fig. S1B). The 3 M-GAS group exhibited downregulation of immune-related functions, including negative regulation of the CaN-NFAT signalling cascade, blood coagulation, and complement activation (classical pathway). Moreover, the 3 M-PM_1_ group exhibited regulated processes related to tissue damage and wound healing, such as ATP biosynthesis, ROS metabolism, wound healing, and DNA geometric changes. DEPs in both the 3 M-GAS and 3 M-PM_1_ groups were enriched in mitochondrial membrane proteins. In addition, the 3 M-PM_1_ group exhibited enriched proteins localised in the sarcoplasmic reticulum (Additional file 4: Fig. S1). Pathway analysis of DEPs suggested common upregulation of oxidative phosphorylation and inositol phosphate metabolism and downregulation of the sirtuin signalling pathway in the 3 M-PM_1_ and 3 M-GAS groups (Fig. 4). The glycolysis and gluconeogenesis pathways were also enriched in both groups; however, inhibition was observed only in the 3 M-GAS group. Inflammation-related pathways, including the acute phase response signalling and mTOR signalling pathways, were activated exclusively in the 3 M-PM_1_ group, whereas the complement system and acute phase response signalling pathway were inhibited in the 3 M-GAS group. Taken together, our results indicate that exposure to TRAP induced early metabolic changes. Subchronic PM_1_ exposure promoted more acute phase responses within lung tissues, whereas exposure to GAS exerted greater suppression on the complement system.

Subsequently, we analysed the injury progression–related biological process within the lung tissues of the 3 M- and 6 M-GAS groups and 3 M- and 6 M-PM_1_ groups. As illustrated in Additional file 5: Fig. S2A and S2B, we observed several tissue development– and wound healing–related processes in both GAS- and PM_1_-exposed rats. The DEPs involved in the inflammatory response and metabolism processes were also enriched. Cellular component mapping indicated that DEPs located in the extracellular vesicle and plasma membrane were upregulated, whereas cytosolic proteins were prominently downregulated in both groups. The pathway enrichment results shown in Fig. 4A indicate exclusive inhibition of inositol phosphate metabolism pathways in lung tissues upon prolonged exposure to gaseous pollutants. The glycolysis I pathway was upregulated in the GAS group but downregulated in the PM_1_ group. Inflammatory pathways including the acute phase response signalling pathway and the complement system were exclusively enriched in the PM_1_ group; however, the net effect was unclear. In addition, we observed some common pathways including activated G beta gamma and calcium signalling as well as inhibited protein kinase A, EIF2 signalling, and LXR/RXR activation in both the GAS and PM_1_ groups. Exposure to gaseous pollutants uniquely enriched the progressive upregulation of pathways related to actin cytoskeleton and Rho family GTPase signalling, which are involved in cell migration, muscle contraction, and potentially tissue repair. The dysregulated biological processes and pathways associated with the progressive injury in CTL groups (by comparing 6 M- and 3 M-CTL groups) are presented in Additional file 5: Fig. S2C and Fig. 4A, whereas those involved in chronic (6 M) exposure to traffic-related gaseous and PM_1_ pollutants (compared with the CTL group) are presented in Additional file 6: Fig. S3 and Fig. 4A.

Regarding particle-specific effects (determined by comparing the PM_1_ and GAS groups), the results of GO analysis suggested that fine particles in the present study prominently upregulated endopeptidase inhibitor activity and humoral immune responses in the subchronic exposure stage, whereas blood coagulation was continuously activated upon prolonged exposure (Additional file 7: Fig. S4). Furthermore, particles downregulated proteins located in chromosomes under subchronic exposure, whereas later at 6 M exposure, downregulated proteins localised within the A band of the muscle fibre were noted (Additional file 7: Fig. S4). Pathway analysis results indicated that during subchronic exposure, particles in TRAP specifically induced higher inflammatory reactions through LXR/RXR activation, acute phase response signalling, and complement responses (Fig. 4B). Upon prolonged exposure, the particles inhibited glycolysis and gluconeogenesis as well as ILK and actin cytoskeleton signalling compared with the GAS group. In summary, from 3 to 6 months of PM_1_ exposure, we observed early induction of inflammatory reactions and a progressive reduction in glucose metabolism and cell movement functions.

### Proposed molecular mechanisms underlying gaseous and PM_1_-induced lung injury

**Fig. 5:**
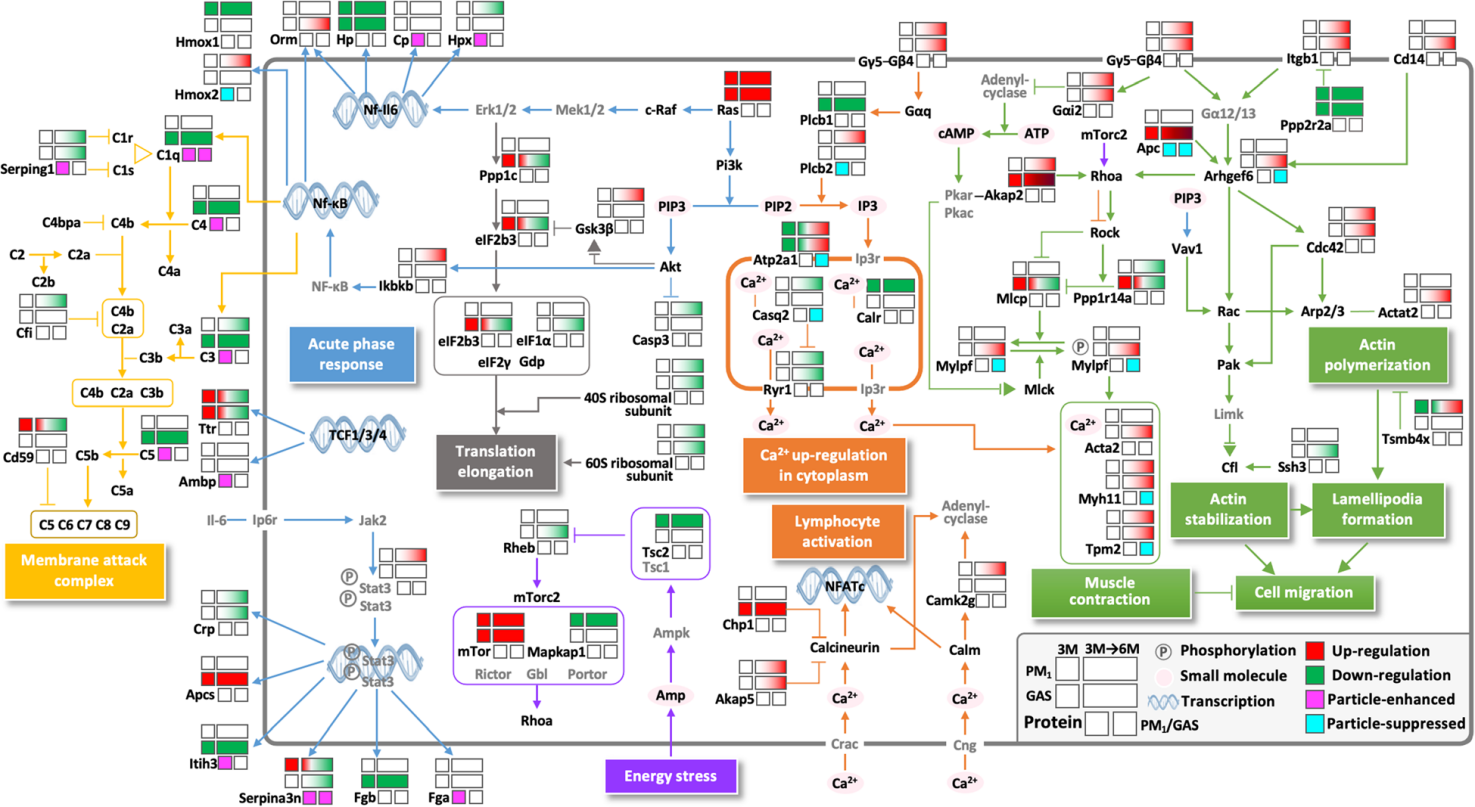
Proposed molecular mechanisms associated with lung injury under subchronic and chronic exposures to traffic-related GAS and PM_1_. Boxes in the upper side of the protein gene symbol indicate the protein expression in PM_1_ (upper) and GAS (lower) groups. Boxes in the left panel of the upper side show the protein ratios of 3 M-PM_1_/CTL and 3 M-GAS/CTL, whereas those in the right panel represent progression from 3- to 6-month exposures (PM_1_-6 M/3 M and GAS-6 M/3 M, respectively). The particle specific regulations are shown as boxes in the right side of the protein gene symbol (3 M-PM_1_/GAS, 6 M-PM_1_/GAS, respectively). Upregulated proteins are marked in red, downregulated proteins in green, and unchanged proteins in white. Protein expression enhanced by particles (PM_1_/GAS) is marked in fuchsia, whereas suppression is marked in cyan

On the basis of the results of functional analysis, we proposed a detailed molecular mechanism (Fig. 5). Changes in the expression of DEPs were displayed using boxes that surrounded the protein gene symbol. First, we highlighted dysregulated proteins and pathways involved in acute phase response signalling and the complement system during subchronic (3 M) exposure to traffic-related GAS and PM_1_; the Ras/Erk to NF-IL6 pathway, Ras/Pi3k/Akt to Nf-κB pathway, Il-6 to Stat3 pathway, and Tcf transcriptional regulations exhibited differential expressions in both 3 M-GAS and 3 M-PM_1_ rats (Fig. 5, the pathways in blue). Proteins responding to oxidative stress, including Ttr, Hp, Hmox1, and the inflammation-related Serpina3, were dysregulated in 3 M-PM_1_ rats compared with 3 M-CTL rats. By contrast, 3 M-GAS rats exhibited upregulation of Ttr and Apcs and downregulation of Hp, Itih3, and Fgb in the acute phase response pathway as well as uniquely downregulated complement proteins (C3, C4, C5, and C1q) in the complement system.

The mTOR complex, which responds to stress and regulates cellular metabolism, cell growth, and cell survival, was dysregulated under subchronic (3 M) exposure to PM_1_ and GAS (Figs. 4A and 5, the pathway in purple). In response to PM exposure, mTOR has been reported to be activated in macrophages [17] or suppressed in the airway epithelium [18], where it protected against both lung injury by attenuation of inflammatory responses and cell death. We observed upregulation of the mTOR pathway, particularly the mTORC2 complex with upregulation of mTor in the GAS and PM_1_ groups, and downregulation of Mapkap1 and inhibitory Tsc2 in the PM_1_ group, suggesting potential protective and homeostasis effects that minimise lung injury. Furthermore, the upregulated Ras/Raf/Mek/Erk signalling promoted Eif2 signalling through upregulation of the proteins Ppp1c and Eif2b3 in the 3 M-GAS group, suggesting elevated production of proteins in response to GAS (Fig. 5, pathways in grey). However, this pathway was gradually downregulated under prolonged exposure to both GAS and PM_1_ with concurrent decreases in 40S and 60S ribosomal protein complexes, resulting in decreased protein synthesis and enhanced apoptosis [19].

Second, we highlighted the dysregulation of the calcium signalling cascade in injury progression (Fig. 5, pathways in orange). In our data, multiple signalling evoking the regulation of intracellular calcium ions (Ca^2+^) exhibited differential expressions in the GAS and PM_1_ groups. The inositol 1,4,5-trisphosphate (IP3) pathway was triggered by progressive elevation of G protein-coupled receptor (represented as Gβ4)/phospholipase C (Plcb2) signalling to release Ca^2+^ from the endoplasmic reticulum to mitochondria and lysosomes, which further regulated metabolic processes. The progressive upregulation of sarcoplasmic/endoplasmic reticulum Ca^2+^-ATPase (presented as Atp2a1) and downregulation of ryanodine receptor (Ryr1) in the GAS and PM_1_ groups suggested restoration and inhibited release of Ca^2+^ from the sarcoplasmic reticulum to cytoplasm in cells. However, we observed the progressive upregulation of intracellular calcium-binding Camk2g (calcium/calmodulin-dependent protein kinase type II subunit gamma) as well as inhibitory Akap5 (A-kinase anchor protein 5) proteins in the PM_1_ group, which suppressed calcium-dependent calcineurin phosphatase activity to control calcium homeostasis. On the basis of these results, we speculated that prolonged exposure to PM_1_ stimulates the accumulation of intracellular Ca^2+^ in the rat lungs, which might promote lymphocyte activation during injury progression. Alternatively, the accumulation of intracellular Ca^2+^ in GAS rats might predominantly promote muscle contraction.

Third, proteins involved in RhoA/Rock-, Rac-, and Cdc42-mediated signalling for controlling muscle contraction, actin dynamics, and cell migration were also impaired during the progression of lung injury (Fig. 5, pathways in green). We observed an unbalanced activation of these three pathways in PM_1_ rats. The RhoA/Rock pathway activated through Gβ4/Gαi2 and mTORC2 ultimately upregulated Acta2, myosin 11 (Myh11), and tropomyosin (Tpm2, 5, 12) complex, which increased contractility in the lungs of both GAS and PM_1_ rats. However, the Rac- and Cdc42-mediated pathways were activated only in GAS rats through the upregulation of Cd14, Arhgef, Apc, and Acta2 and the downregulation of inhibitory Tmsb4 and Ssh3 proteins, promoting actin polymerisation and stabilisation and subsequent cell migration. These results suggested the activation of myosin–axis contraction but an absence of actin dynamics under prolonged exposure to traffic-related PM_1_.

### Functional validation

**Fig. 6:**
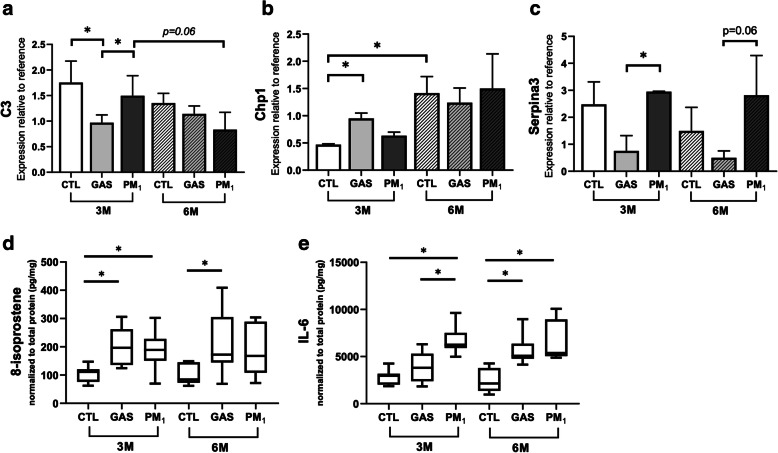
Functional validation of selected proteins, oxidative stress, and IL-6 in lung tissues. **a**, **b**, and **c** present the statistical results of Western blot analysis for C3, Chp1, and Serpina3 proteins in lung tissues, respectively. Vinculin was used as the loading control. The expression level of each protein was further normalized to that in the reference sample running in every gel for between-gel comparison. **d** and **e** present the ELISA results of 8-isoprostane and IL-6, representing the status of oxidative stress and inflammation in lung tissues, respectively. The quantities of 8-isoprostane and IL-6 were normalized to the total protein lysate before comparison. * *p* < 0.05

Among discovered DEPs, we selectively validated the expression levels of inflammatory related proteins, C3, Chp1, and Serpina3, in the rat lungs through Western blotting. The representative images are shown in Additional file 8: Fig. S5. The statistical results shown in Fig. 6A indicate that C3 was significantly downregulated in 3 M-GAS rats compared with 3 M-CTL and 3 M-PM_1_ rats, whereas no significant difference was observed in the 6 M exposure groups. A significantly higher and slightly higher expression of Serpina3 were observed in the 3 M-PM_1_ and 6 M-PM_1_ groups, respectively, than in the corresponding GAS groups (Fig. 6C). The inhibitory Chp1 protein was upregulated in only the 3 M-GAS group. In addition, we investigated oxidative stress and inflammatory profiles within the lung tissue by measuring 8-isoprostane and IL-6 by performing the enzyme-linked immunosorbent assay (ELISA), respectively. The results revealed a higher level of oxidative stress in both the 3 M-GAS and 3 M-PM_1_ groups, whereas only the 6 M-GAS group exhibited higher oxidative stress than did the CTL group (Fig. 6D). Notably, a higher IL-6 level was observed in the rat lungs in the 3 M-PM1 group than in the GAS and CTL groups, whereas the IL-6 level did not significantly differ between the 6 M-PM_1_ and 6 M-GAS groups (Fig. 6E). These results suggest earlier occurrence of oxidative stress and inflammatory reaction in 3 M-PM_1_- and GAS-exposed rats; this finding is consistent with proteomics data.

## Discussion

Epidemiological studies have reported that prolonged exposure to traffic-related air pollution is associated with declined lung function [20, 21]; however, the detailed molecular mechanism remains unclear. Thus, we investigated how traffic-related air pollution affects the lungs in the rat model with whole-body exposure that we previously reported [16]. Our whole-body exposure system had a higher penetration rate for PM_1_, which is in the range of ultrafine to fine PM [16]. The levels of air pollution during the study period have been reported previously [16]. Briefly, continuous monitoring of ambient air exposure characteristics revealed that the PM_1_ mass concentration was 16.3 ± 8.2 (4.7–68.8) μg/m^3^ with a geometric mean diameter of 55.8 ± 7.3 (40.3–74.5) nm. The particle number concentration was 11,257 ± 4388 (2218–25,733) particles/m^3^. The average ambient PM_2.5_ was 19.7 ± 9.8 μg/m^3^, which was obtained from the Yonghe air quality monitoring station. The PM_1_ to PM_2.5_ ratio was 0.827, which is commonly observed in traffic-dominated urban areas. The results of correlation analysis between PM_1_ and PM_2.5_ revealed an *r*^2^ of 0.829 (*p* < 0.001) [22], suggesting that rats were mainly exposed to outdoor air pollution during experimental periods. The temperature was 20 °C ± 4 °C (12 °C–29 °C) and relative humidity was 72% ± 9% (47–92%) throughout the study period. The lung-deposited surface area in the alveolar region was 55.1 ± 21.7 (20.7–136.6) μm^2^/cm^3^. The black carbon (BC) mass concentration was 1800 ± 784 (219–4732) ng/m^3^. The gaseous pollutant profile indicated NOx, SO_2_, and O_3_ levels of 32.9 ± 16.4 (8.4–86.6) ppb, 2.5 ± 1.0 (0.2–5.0) ppb, and 29.7 ± 11.0 (6.7–58.2) ppb, respectively [16, 23].

In the present study, we observed a statistically significant decline in lung function with decreased FEV_20_/FVC after 3 months of pulmonary exposure to traffic-related PM_1_ (compared to CTL group, Fig. 1B). After 6-month exposure to traffic-related PM_1_, the rats further exhibited decreased FEF_25–75%_ in comparison to both CTL and GAS groups (Fig. 1A). The histological examination of the lung tissues also revealed a significant lung injury under subchronic (3 M) exposure to PM_1_, as characterised by increased levels of congestion and macrophage infiltration (Table 1). However, no significant lung injury was observed in rats in the 3 M- and 6 M-GAS groups, even though FEF_25–75%_ decreased in 6 M-GAS rats. These results suggested that the presence of PM caused lung damage and lung function decline under pulmonary exposure to traffic-related PM_1_. Similar to our findings, another study on rats exposed to biomass fuel and vehicle exhaust pollutants reported increased leukocyte counts in bronchoalveolar lavage and accumulated inflammatory cells in airway walls as early as 1–3 months of exposure, whereas a significant reduction in lung functions was observed at only 7 months of exposure [7]. A multitude of studies described comparable, if not greater, alterations of pulmonary function [24], immune cell accumulation, and expression of gamma-glutamyl transpeptidase, a marker of injury in the bronchoalveolar lavage fluid [25, 26] resulted from exposure to the gaseous and whole component of diesel exhaust allergen. Furthermore, the Advanced Collaborative Emissions Study reported similar lung pathology in rodents upon exposure to unfiltered diesel exhaust and NO_2_ apart [27]. Therefore, filtration of particulate matter may not appreciably attenuate the health effect associated with exposure to air pollution [28].

To elucidate the underlying mechanism, we systematically studied perturbed functions and pathways underlying lung injury upon subchronic and chronic exposure to traffic-related PM_1_ and GAS by performing MS-based quantitative proteomics analysis (Figs. 3 and 4, and Additional files 4, 5, 6 and 7: Figs. S1–4). The functional analysis of DEPs in 3 M-PM_1_ and 3 M-GAS rats revealed early dysregulation of lipid, glucose, and protein metabolism in acute phase response signalling and the complement system, which may directly or indirectly contribute to lung injury or lung protection under subchronic exposure to PM_1_ and GAS. Notably, GAS significantly suppressed acute phase response signalling and the complement system, whereas PM_1_ upregulated them to the same levels as in the CTL group. As injury progressed, metabolic pathways (except glycolysis I) were mainly downregulated or not enriched, whereas actin dynamics–related functions and pathways were activated, especially in rats in the GAS group. Nevertheless, rats in the 6 M-PM_1_ group exhibited a significant decline in lung function through decreased FEV_20_/FVC and FEF_25–75%_, which might have been caused by the failure to trigger the actin dynamics pathway for wound healing. Our data also suggested that the presence of fine particles specifically inhibited glucose metabolism and actin cytoskeleton signalling after chronic exposure. The temporal and dynamic proteome changes observed in this study indicated differential and complex regulation at cellular and molecular levels in the lungs caused by traffic-related PM_1_ and GAS.

Fine and ultrafine PMs have been reported to induce lung diseases through the generation of ROS and oxidative stress as well as the activation of innate and adaptive immunity, leading to cell barrier and tissue damage [29, 30]. Several known promoters of inflammatory responses, such as NF-κB, activation protein-1, nuclear factor erythroid 2-related factor 2, and CREB-binding proteins, are activated by oxidative stress [31–33]. In this study, we observed significantly higher levels of oxidative stress and IL-6 together with the upregulation of oxidative phosphorylation and acute phase response signalling in rats exposed to PM_1_ for 3 months. By contrast, 3 M-GAS rats exhibited higher levels of oxidative stress but no difference in IL-6 expression compared with 3 M-CTL rats, possibly because of upregulated oxidative phosphorylation and downregulated acute phase response signalling and complement system. As key components in the innate immune system, pathogen infection and tissue damage trigger the complement system, which further promotes chemotaxis [34], activates neutrophils and macrophages for chemokine secretion [35], and exacerbates acute lung injury through autophagy-mediated alveolar macrophage apoptosis [36]. Walters et al. reported that PM_2.5_-treated mice underwent airway hyper-responsiveness resulting from the activation of C3 [37]. In adults older than 65 years in China, short-term exposure to PM_2.5_ resulted in a significant increase in serum complement C3 and inflammatory reaction [38]. Ultrafine PM (PM_0.1_) enhanced pulmonary inflammation through the rapid influx of neutrophils and the secretion of proinflammatory cytokines [39] and also induced higher IL-6 release from A549 cells compared with coarse particles [40]. Moreover, particles < 100 nm may penetrate deep into the respiratory tract, be absorbed by the blood stream, and ultimately be deposited in organs [41]. Because our rat model was mainly exposed to ultrafine and fine PM, we similarly observed an elevation in oxidative stress in the lungs and higher immune cell infiltration, which contributed to the activation of acute phase signalling pathways and thus to significant lung injury in 3 M-PM_1_ rats (Table 1 and Fig. 2C). However, we noted that proteins involved in the complement system and acute phase response signalling did not exhibit significant changes upon chronic exposure to PM_1_ and GAS. Thus, we speculated that acute phase response signalling and complement activation may be early events mediating pulmonary inflammation upon PM_1_ or GAS exposure. Such early inflammatory regulation can be characterised as unique molecular features in the subchronic stage of lung injury.

PM infiltration in the lungs can disrupt cell membrane integrity and subsequently increase the intracellular calcium ion (Ca^2+^) concentration. Calcium signalling affects a broad spectrum of cellular functions such as motility, metabolism, cell growth, proliferation, and apoptosis [42]; moreover, the dysregulation of intracellular Ca^2+^ has been reported to be associated with PM-induced oxidative stress and inflammation in human lung fibroblast cells [43], pulmonary artery endothelial cells [44], and mouse lungs [45]. Proteins that control the efflux and influx of calcium play crucial roles in calcium homeostasis. In cystic fibrosis, decreased SERCA expression and activity were observed to increase the susceptibility of airway epithelial cells to oxidant gas exposure and cell death [46, 47]. In our model, we observed dysregulation of IP3-, SERCA-, and calmodulin/calcineurin-mediated calcium signalling. Among involved DEPs, SERCA (as Atp2a1, sarcoplasmic/endoplasmic reticulum calcium ATPase 1) was inhibited in both the 3 M-PM_1_ and 3 M-GAS groups, probably as an early response to TRAP. SERCA was later upregulated in both 6 M-PM_1_ and 6 M-GAS rats together with a lower expression of Ryr1, which could have been an attempt to limit Ca^2+^ leakage from the sarcoplasmic/endoplasmic reticulum and balance the elevated cytoplasmic Ca^2+^ concentration. The high intracellular Ca^2+^ concentration was also reported to promote NFATc signalling and subsequent activation of lymphocytes [48], demonstrating its role as a proinflammatory mediator. Despite the biological significance of calcium signalling in lung injury, how this dysregulated calcium signalling coordinately contributes to PM- or GAS-induced oxidative stress, metabolism, and inflammation remains unclear. Therefore, more cellular and molecular studies are required to address these issues.

The accumulation of Ca^2+^ in the cytoplasm may serve as a signal to initiate wound healing in injured rat lungs [49]. Wound healing and tissue repair are a multistep process composed of wound sensing and blocking, plasma membrane restoration, and cytoskeleton remodelling. Although the detailed wound healing mechanism is not yet clear, it is reported to be tightly controlled by Rho family GTPase pathways, including RhoA/Rock, Rac, and Cdc42 signalling [50–52]. In our model, Rhoa/Rock-mediated actomyosin contraction was activated in response to prolonged exposure to PM_1_ and GAS by upregulated Gβ4 and Itgb1 proteins, the activated mTORC2 complex, and increased intracellular Ca^2+^ levels, suggesting the initiation of repair for membrane resealing and wound closure. We noted that rats in the GAS group exhibited additional upregulation of Mlcp, Ppp1r14a, Mylpf, and Acta2, which triggered actin–myosin–tropomyosin complexes for muscle contraction. In addition, proteins involved in Rac signalling (Apc and Arhgef6) and Cdc42 signalling (Cdc42 and Actat2) were uniquely upregulated in GAS rats but not activated or inhibited (through increased expression of inhibitory Tsmb4x) in PM_1_ rats; thus, rats exposed to prolonged traffic-related PM were unable to trigger actin polymerisation and stabilisation for cytoskeleton remodelling [53], thereby limiting the potential for tissue repair and ultimately leading to declined lung function. The concomitant upregulation of these Rho family GTPase-mediated pathways was exclusively observed in GAS-exposed rats, highlighting the role of PM in disrupting repair potential. By contrast, the progressive upregulation of actin, myosin, and tropomyosin protein complexes in PM_1_ rats may represent the increased quantity of the smooth muscle, as observed in the thickening of the airway wall, or possible fibrosis formation within the lung tissue [54].

Although not included in Fig. 5, both GAS and PM_1_ pollutants stimulated alterations in metabolic pathways, notably in oxidative phosphorylation, lipid (inositol phosphate superpathways), and glucose (glycolysis/gluconeogenesis) metabolism. Increased oxidative phosphorylation was observed in both the 3 M-PM_1_ and 3 M-GAS groups, which may imply impaired mitochondrial functions, higher oxidative stress [55], and probably a compensation of metabolic shifting from glycolysis to the pentose phosphate pathway [56]. Glycolysis was reported to worsen infection-related pulmonary fibrosis [57], and inhibition of glycolysis has been demonstrated to attenuate injury by suppressing inflammation and apoptosis [58, 59]. Concurrently, our model exhibited negative regulation of glycolysis/gluconeogenesis in chronic PM_1_-exposed rats, suggesting a potential mechanism for limiting lung injury. Regarding lipid metabolism, a recent metabolomics study reported that an organic component of PM_2.5_, namely benzo[a]pyrene, induces lung injury through altering lipid metabolism and phospholipase A2 activity [60]. Downregulation of lipid metabolism and upregulation of glucose metabolism mediated by autophagy were also reported to be involved in alveolar repair after bleomycin-induced injury [61], whereas another study reported that inhibition of lipid synthesis exacerbated bleomycin-induced lung fibrosis [62]. These controversial results indicate that the interplay between lipid and glucose metabolism induced by PM remains poorly characterised.

Overall, we studied the effect of traffic-related air pollution on lung injury by using our previously established rat model to mimic dynamic day-to-day exposure in people. We elaborated the lung function and histological changes as well as elucidated dysregulated molecular mechanisms upon subchronic and chronic exposure to traffic-related PM_1_ and GAS by performing quantitative proteomics analysis. A detailed molecular mechanism was proposed, and differential regulations were suggested among gaseous and PM pollutants. However, this study has several limitations that should be addressed. The use of HEPA filters in air purifiers yields the lowest clean air delivery rate for particles < 0.1 μm [63]; thus, the possibility of rats in the GAS group being exposed to particles < 0.1 μm cannot be ruled out. In addition, the composition of PM pollutants and exposure times could be potential factors causing inconsistencies in observations compared with those of previous studies. Because of the unclear pathogenesis underlying gaseous and PM pollution, more cellular and molecular studies are required to evaluate temporal changes induced by such pollutants, especially mechanisms involved in metabolism, tissue repair, and calcium signalling. To translate our findings into potential clinical applications, more studies should be conducted to detect observed dysregulated proteins in the peripheral blood of high-risk human populations, thus evaluating their potential as noninvasive biomarker candidates.

## Conclusion

This study systematically explored phenotypes and pathogenic mechanisms in the rat lungs upon subchronic and chronic exposure to traffic-related air pollution. The in-depth quantitative tissue proteomics analysis explored detailed molecular mechanisms involved in the progression of lung injury, which eventually led to the disturbance of lung functions. According to the findings, we proposed several potential proteins associated with early lung damage in response to traffic-related PM_1_ or GAS, which might be used to screen individuals more susceptible to air pollution.

## Methods

### Chemicals and reagents

CelLytic™ MT cell lysis reagent, ammonium hydroxide solution, formic acid (FA), triethylammonium bicarbonate (TEABC), and Tween-20 were purchased from Sigma-Aldrich (Saint Louis, MO, USA). Trifluoroacetic acid was purchased from Wako (Osaka, Japan). Clarity™ Western ECL Substrate and nitrocellulose membranes were purchased from Bio-Rad (Hercules, CA, USA). Acetonitrile (ACN) was purchased from Spectrum (California, USA). Ethylenediaminetetraacetic acid (EDTA) and protease inhibitors were purchased from G-Biosciences (St. Louis, MO, USA). Trypsin was purchased from Promega (Madison, USA). The bicinchoninic acid (BCA) protein assay kit and TMT assay were purchased from Thermo Fisher Scientific (Rockford, USA). Furthermore, 10% neutral buffered formalin was purchased from CHIN IPAO CO., LTD (Taipei, Taiwan), paraffin was purchased from Leica Microsystems Pvt. Ltd. (Macquarie Park, Australia), and haematoxylin and eosin (H&E) were purchased from Roche Diagnostics (Indianapolis, USA).

### Rat model with whole-body exposure to TRAP

Male six-month-old Sprague-Dawley (SD) rats weighing 600–700 g were obtained from the National Laboratory Animal Center (Taipei, Taiwan) and housed at a constant temperature of 22 °C ± 2 °C, a relative humidity of 55% ± 10%, and a 12-h light–dark cycle. Our whole-body exposure system, which we previously presented in [16], was used to mimic TRAP exposure in humans. Briefly, ambient air was continuously sampled using an omnidirectional PM inlet located on the roof of the animal housing. The stream was introduced into each cage of the whole-body exposure system. Simultaneously, a stream was sampled from an empty whole-body exposure cage (without rats) to characterise the physical features of PM. All procedures were performed in compliance with the Animal and Ethics Review Committee of the Laboratory Animal Center at Taipei Medical University (Taipei, Taiwan).

Rats were randomly assigned to three groups for exposure in two different periods: (1) three and six months of exposure to whole air from TRAP (3 M-PM_1_ and 6 M-PM_1_ groups, respectively); (2) three and six months of exposure to HEPA-filtered TRAP (3 M-GAS and 6 M-GAS groups, respectively); and (3) three and six months of exposure to HEPA-filtered conditioned clean air (3 M-CTL and 6 M-CTL groups, respectively). Each group had 16 rats. The PM_1_ and GAS groups were placed in an urban region near a major highway and expressway in New Taipei City, Taiwan. The average daily traffic volume is 444 cars/hour and 4731 motorcycles/hour during the evening rush hour (17:00–20:00) and 462 cars/hour and 2183 motorcycles/hour during the morning rush hour (06:00–08:00) [64]. The CTL group was housed in a specific-pathogen-free level of the Laboratory Animal Center (Taipei Medical University).

All PM_1_ exposure on rats was determined in the exposure system as reported previously [64, 65]. Briefly, a tapered element oscillating microbalance (1400a, Thermo Fisher Scientific) was used to monitor the particle mass concentration. A scanning mobility particle sizer (TSI 3936) and an aerodynamic particle sizer (TSI 3321) were used to characterize submicron and supermicron particle number concentrations, respectively. A nanoparticle surface area monitor (TSI 3550) was used to monitor the lung deposition surface area concentration in the alveolar region. The BC mass concentration was measured using an aethalometer (Magee Scientific AE33, Berkeley, CA, USA). Gaseous pollution was referenced from the nearest Taiwan Environmental Protection Agency air quality station.

### Lung function examination

Body weight was measured before lung function test [16]. Invasive lung function measurement was examined using a ventilated bias flow whole-body plethysmograph (WBP) (BioSystem XA, DSI, Wilmington, NC) under anaesthesia by Zoletil, which consisted of a reference chamber and animal chamber interconnected by a pressure transducer (MAX1320, Buxco Electronics, Sharon, CT). According to the Buxco pulmonary maneuvers protocol and previous reports [66–68], data for forced expiratory flow at 25–75% of forced vital capacity (FEF_25–75%_) and forced expiratory volume in 20 ms to forced vital capacity (FEV_20_/FVC) were presented. At least three acceptable maneuvers for each test of every rat were conducted to obtain a reliable mean spirometry data.

### Histological evaluation

Lung tissues were collected, washed with ice-cold phosphate-buffered saline, and fixed through tracheal instillation of 10% buffered formalin at a pressure of 25 cmH_2_O for 10 min. Subsequently, these tissues were embedded in paraffin, sectioned, and stained with H&E. Histological examinations were conducted under a light microscope by a histopathologist in a blinded manner [69]. The degree of lung injury was scored according to four criteria: (1) alveolar congestion, (2) haemorrhage, (3) immune cell infiltration, and (4) thickness of the alveolar wall [69]. The extent of alveolar congestion was measured by the degree to which red blood cells (RBCs) congested and dilated the lumen of capillaries within the alveolar wall. The severity of congestion and thickness of the alveolar wall were graded on a five-point scale as follows: 0 = minimal (little), 1 = mild, 2 = moderate, 3 = severe, and 4 = maximal. Haemorrhage was graded as follows: 0 = no RBCs outside of blood vessels, 1 = a few interstitial RBCs, 2 = a few RBCs in some alveoli, 3 = a moderate number of RBCs in some alveoli, 4 = many RBCs in most alveoli, and 5 = large numbers of RBC in all alveoli. Infiltration of macrophages was graded as follows: 0 = none or rare, 1 = 1–10% of alveoli/saccules contain macrophages, 2 = 10–25%, 3 = 25–75%, and 4 = > 75% [70].

### Tissue lysate collection

The lung tissues from each rat were grounded in liquid nitrogen, collected in a microcentrifuge tube, and lysed with CelLytic MT cell lysis reagent, protease inhibitors, and EDTA at a volume ratio of 98:1:1. The tissue lysate was homogenised using a Minilys® personal homogeniser (Bertin, Rockville, MD, USA) on a high-speed mode for 15 s twice, and the homogenate was centrifuged at 13,000 rpm at 4 °C for 10 min to collect the clear supernatant as the lung tissue lysate. The lung tissue lysate from each rat was assayed using the BCA protein assay kit to determine the protein concentration.

### Gel-assisted digestion, TMT labelling, and high pH reverse phase StageTip fractionation

To elucidate molecular mechanisms underlying TRAP-induced lung injury, we performed TMT-based quantitative proteomics analysis by using the lung tissues obtained from the six exposure groups. Briefly, 50 μg of lung tissue proteins were aliquoted from five randomly selected rats from each group, and gel-assisted digestion was individually performed using trypsin [71]. The resulting peptides were vacuum dried and resuspended in 100 mM TEABC for the BCA protein assay; 10 μg of peptides were aliquoted from each of the five rats from a particular exposure group to generate a pooled peptide sample. The pooled peptides from the 3 M-CTL, 3 M-PM_1_, and 3 M-GAS groups were labelled with TMT126, TMT127, and TMT128, respectively, whereas those from the 6 M-CTL, 6 M-PM_1_, and 6 M-GAS groups were labelled with TMT129, TMT130, and TMT131, respectively. The TMT-labelled peptides from each group were pooled for reverse phase StageTip fractionation following the protocol reported in [72]. Peptides were sequentially eluted using 11.1, 14.5, 17.4, 19, 23, and 27%–80% ACN in ammonium hydroxide solution (pH 11.5). The peptides from each fraction were vacuum dried and resuspended in 0.1% FA for LC-MS/MS analysis.

### NanoLC–nanoESI-MS/MS analysis

NanoLC–nanoESI-MS/MS analysis was performed using a Thermo UltiMate 3000 RSLCnano system connected to an Orbitrap Fusion™ Tribrid™ Mass Spectrometer (Thermo Fisher Scientific, Bremen, Germany) equipped with a nanospray interface (New Objective, Woburn, MA). Peptide mixtures were loaded onto a 75-μm ID, 25-cm PepMap C18 column (Thermo Fisher Scientific) packed with 2-μm particles with a pore width of 100 Å. The peptides were eluted using a 103-min gradient using 5 to 45% mobile phase B (99.9% ACN, 0.1% FA in HPLC-grade water) at a flow rate of 0.4 μL/min. The gradients were slightly adjusted for each RP fraction.

LC-MS/MS experiments were performed in a data-dependent acquisition mode to sequentially select the top 15 most intense precursor ions for higher-energy collision dissociation with a normalised collision energy of 40%. Full MS scans were acquired in Orbitrap from *m/z* 300–1600 with a resolution of 120,000 and automated gain control of 400,000 charges or maximum ion time of 50 ms. For MS/MS scans, fragment ions were acquired in Orbitrap with a resolution of 60,000 and automated gain control of 1E5 or a max ion time of 100 ms. Precursors with assigned charge states from 2+ to 7+ were included. Previously targeted precursors were dynamically excluded from reacquisition for 15 s.

### Proteome identification and quantification

LC-MS raw data were searched against the SwissProt *Rattus norvegicus* database (version 2018_11, 8054 entries) by using Mascot implemented in Proteome Discoverer (version 2.2.0.388, Thermo Fisher). The MS and MS/MS tolerances were set to 20 ppm and 0.1 Da, respectively. Tryptic peptides with a maximum of two missed cleavages were allowed. Methylthio (Cys) was set as a fixed modification, whereas oxidation (Met), acetylation (protein N-terminal), deamidation (Asn and Gln), and TMT tags (N-terminal, Lys) were set as variable modifications. Next, 1% FDR was applied in peptide spectral matches and peptide and protein levels for confident identification. Peptides identified with high confidence with at least six amino acids were accepted. For proteome quantification, only unique peptides were included to estimate protein abundance, which was further normalised by the total peptide abundance. Proteins with 1.3-fold changes in abundance (log2 ratio more than 0.38 or less than − 0.38) were considered DEPs.

### Functional enrichment analysis

DEPs from each exposure group were uploaded to IPA [73] for pathway enrichment analysis as well as Cytoscape (version 3.7.1) with ClueGo (version 2.5.4) plugin [74, 75] for Gene Ontology (GO) analysis (*Rattus norvegicus* database, v.20.05.2019). The GO fusion and GO group were selected, whereas other settings were left on default. Only GO terms from the biological process, molecular function, and cellular component with the lowest term *p* value (corrected with Bonferroni step down) were included for analysis. For pathway enrichment analysis using IPA, z-scores were obtained for the enriched canonical pathway as well as disease and biofunction annotations. Annotations with *p* < 0.05 were considered significant. A z-score of > 0 indicated activation, whereas that of < 0 indicated inhibition of a cellular function or pathway.

### Western blot analysis

Subsequently, 40 μg protein from 4 to 5 rats in each group was run in gel electrophoresis by using 4–20% Mini-PROTEAN® TGX™ Gel (Bio-Rad, CA, USA), followed by protein transfer onto nitrocellulose membranes. After blocking for 1 h at room temperature, the membranes were incubated overnight at 4 °C with primary antibodies against Chp1, Serpina3 (1:1000, ABclonal technology, MA, USA), C3, or Vcl (1:1000, both from Santa Cruz, TX, USA), separately. The membranes were thoroughly washed with phosphate-buffered saline with Tween-20 and then incubated with the antirabbit or antimouse IgG secondary antibody (1:10000, Bioss Antibodies, MA, USA) for 1 h at room temperature. Clarity™ Western ECL Substrate was used to detect protein bands. Vinculin (Vcl) was used as the loading control, and one rat sample from the 6 M-CTL group was included in each gel as a reference for between-gel comparison. The detected protein bands were quantified using AzureSpot (Azure Biosystems, CA, USA).

### Elisa

The ELISA approach was adopted to measure 8-isoprostane (Cayman, USA) and IL-6 (R&D System, Minneapolis, MN, USA) in the lung tissue lysate following the manufacturers’ instructions. Data are presented after normalisation to the total protein amount.

### Statistical analysis

The lung function and lung injury results are reported as medians with interquartile ranges. The Mann–Whitney U test was performed to evaluate significant differences; only differences with *p* < 0.05 were considered significant. Statistical analyses were performed using GraphPad (version 8).

## Supplementary Information

**Additional file 1: Table S1.** The differentially expressed proteins (DEPs) in 3 month GAS- and PM1-exposed groups

**Additional file 2: Table S2.** The progression related DEPs in GAS- and PM1exposed groups

**Additional file 3: Table S3.** The PM-specific DEPs in 3 M- and 6 M-exposed groups

**Additional file 4: Figure S1.** The Gene Ontology analysis of DEPs in subchronic exposure groups. The enriched biological process, molecular function and cellular component in (A) 3 M-GAS group and (B) 3 M-PM1 group with comparison to 3 M-CTL group. The percentage of up-regulated proteins involved in each enriched term is indicated next to bar.

**Additional file 5: Figure S2**. The Gene Ontology analysis of DEPs in progressive exposure to GAS, PM1, and CTL groups. The enriched biological process, molecular function and cellular component in (A) GAS group, (B) PM1 group, and (C) CTL group. The percentage of up-regulated proteins involved in each enriched term is indicated next to bar.

**Additional file 6: Figure S3**. The Gene Ontology analysis of DEPs in chronic exposure groups. The enriched biological process, molecular function and cellular component in (A) 6 M-GAS group and (B) 6 M-PM1 group with comparison to 6 M-CTL group. The percentage of up-regulated proteins involved in each enriched term is indicated next to bar.

**Additional file 7: Figure S4**. The Gene Ontology analysis of DEPs specifically regulated by particles. The enriched biological process and molecular function specifically regulated by particles under (A) 3-month and (B) 6-month exposures. The percentage of up-regulated proteins involved in each enriched term is indicated next to bar.

**Additional file 8: Figure S5**. Western blot validation. The representative Western blot results for expression levels of C3, Serpina3 and Chp1 in rat lung tissues. A reference sample was run in every analysis for normalization. Vinculin is served as loading control.

## Data Availability

The data sets used and/or analysed during the current study are available from the corresponding author upon reasonable request.

## Abbreviations

PM: Particulate matter
TRAP: Traffic-related air pollution
HEPA: High-efficiency particulate air
GAS: HEPA-filtered traffic-related gaseous pollutants
CTL: Clean air control
LC-MS/MS: Liquid chromatography tandem mass spectrometry
TMT: Tandem mass tag
RP: Reverse phase
DEP: Differentially expressed protein
GO: Gene ontology
IPA: Ingenuity Pathway Analysis
ROS: Reactive oxygen species
C3: Complement 3
Chp1: Calcineurin B homologous protein 1
Serpina3: Serine protease inhibitor A3K
IL: Interleukin
Atp2a1: Sarcoplasmic/endoplasmic reticulum calcium ATPase 1
Camk2g: Calcium/calmodulin-dependent protein kinase type II subunit gamma
Ryr1: Ryanodine receptor 1
Akap5: A-kinase anchor protein 5
Vcl: Vinculin
ELISA: Enzyme-linked immunosorbent assay
FA: Formic acid
ACN: Acetonitrile
FDR: False discovery rate
RBC: Red blood cells
FEF_25–75%_: Forced expiratory flow at 25–75% of the pulmonary volume
FEV_20_: Forced expiratory volume at 20 ms
FVC: Functional vital capacity
BCA: Bicinchoninic acid.
