# Supplementary material for: Prolonged exposure to traffic-related particulate matter and gaseous pollutants implicate distinct molecular mechanisms of lung injury in rats
Source: Part Fibre Toxicol. 2021 Jun 25;18:24. doi: 10.1186/s12989-021-00417-y (PMC8235648; doi:10.1186/s12989-021-00417-y)
Supplement: Supplementary file 1 — Additional file 1: Table S1. The differentially expressed proteins (DEPs) in 3 month GAS- and PM1-exposed groups [file 12989_2021_417_MOESM1_ESM.pdf]

Supplementary Information

Prolonged Exposure to Traffic-related Particulate Matter and Gaseous Pollutants Implicate Distinct Molecular Mechanisms of Lung Injury in Rats

Yu-Teng Jheng[1&], Denise Utami Putri[2,3&], Hsiao-Chi Chuang[4], Kang-Yun Lee[5,6], Hsiu-Chu Chou[7], San-Yuan Wang[1], Chia-Li Han[1*]

[1]*Master Program in Clinical Pharmacogenomics and Pharmacoproteomics, College of Pharmacy, Taipei, Taiwan*

[2]*International Ph.D. Program in Medicine, College of Medicine, Taipei Medical University, Taipei, Taiwan*

[3]*Pulmonary Research Center, Division of Pulmonary Medicine, Department of Internal Medicine, Wan Fang Hospital, Taipei Medical University, Taipei, Taiwan*

[4]*School of Respiratory Therapy, College of Medicine, Taipei Medical University, Taipei, Taiwan*

[5]*Division of Pulmonary Medicine, Department of Internal Medicine, School of Medicine, College of Medicine, Taipei Medical University, Taipei, Taiwan*

[6]*Division of Pulmonary Medicine, Department of Internal Medicine, Shuang Ho Hospital, Taipei Medical University, New Taipei City, Taiwan*

[7]*Department of Anatomy and Cell Biology, School of Medicine, College of Medicine, Taipei Medical University, Taipei, Taiwan*

[&] These authors contributed equally to this work

[*]Corresponding author

List of Supplementary Materials

Additional file 1: Table S1. The differentially expressed proteins (DEPs) in 3-month GAS- and PM_1-exposed groups

Additional file 2: Table S2. The progression related DEPs in GAS- and PM_1-exposed groups

Additional file 3: Table S3. The PM-specific DEPs in 3M- and 6M-exposed groups

Additional file 4: Figure S1. The Gene Ontology analysis of DEPs in subchronic exposure groups. The enriched biological process, molecular function and cellular component in (**A**) 3M-GAS group and (**B**) 3M-PM_1 group with comparison to 3M-CTL group. The percentage of up-regulated proteins involved in each enriched term is indicated next to bar.

Additional file 5: Figure S2. The Gene Ontology analysis of DEPs in progressive exposure to GAS, PM_1, and CTL groups. The enriched biological process, molecular function and cellular component in (**A**) GAS group, (**B**) PM_1 group, and **(C)** CTL group. The percentage of up-regulated proteins involved in each enriched term is indicated next to bar.

Additional file 6: Figure S3. The Gene Ontology analysis of DEPs in chronic exposure groups. The enriched biological process, molecular function and cellular component in (**A**) 6M-GAS group and (**B**) 6M-PM_1 group with comparison to 6M-CTL group. The percentage of up-regulated proteins involved in each enriched term is indicated next to bar.

Additional file 7: Figure S4. The Gene Ontology analysis of DEPs specifically regulated by particles. The enriched biological process and molecular function specifically regulated by particles under (**A**) 3-month and (**B**) 6-month exposures. The percentage of up-regulated proteins involved in each enriched term is indicated next to bar.

Additional file 8: Figure S5. Western blot validation. The representative Western blot results for expression levels of C3, Serpina3 and Chp1 in rat lung tissues. A reference sample was run in every analysis for normalization. Vinculin is served as loading control.

Table S1. The differentially expressed proteins (DEPs) in 3-month GAS- and PM1-exposed groups

Accession	Gnen name	Description	#Unique Peptides	#PSMs	MW [kDa]	Mascot Score	3M-GAS/CTL (log_2Ratio)	3M-PM1/CTL (log_2Ratio)
P01836		Ig kappa chain C region, A allele OS=Rattus norvegicus OX=10116 PE=1 SV=1	2	91	11.7	1389	↑ 3.28	↑ 4.14
O08949	Gtf2a1	Transcription initiation factor IIA subunit 1 OS=Rattus norvegicus OX=10116 GN=Gtf2a1 PE=1 SV=1	1	1	41.5	22	↑ 1.15	↑ 0.53
Q9JK71	Magi3	Membrane-associated guanylate kinase, WW and PDZ domain-containing protein 3 OS=Rattus norvegicus OX=10116 GN=Magi3 PE=1 SV=2	7	15	160.5	188	↑ 1.07	→ 0.09
Q99P55	Sgpp1	Sphingosine-1-phosphate phosphatase 1 OS=Rattus norvegicus OX=10116 GN=Sgpp1 PE=1 SV=2	1	1	47.6	0	↑ 0.92	↑ 0.41
Q5PQJ7	Tbcel	Tubulin-specific chaperone cofactor E-like protein OS=Rattus norvegicus OX=10116 GN=Tbcel PE=1 SV=1	1	2	48	21	↑ 0.91	→ 0.02
Q5M7T2	Spryd7	SPRY domain-containing protein 7 OS=Rattus norvegicus OX=10116 GN=Spryd7 PE=2 SV=1	2	3	21.7	76	↑ 0.82	→ 0.37
Q923V4	Fbxo6	F-box only protein 6 OS=Rattus norvegicus OX=10116 GN=Fbxo6 PE=1 SV=1	5	11	32.8	93	↑ 0.81	→ -0.26
Q01812	Grik4	Glutamate receptor ionotropic, kainate 4 OS=Rattus norvegicus OX=10116 GN=Grik4 PE=1 SV=1	1	3	107.2	38	↑ 0.76	↑ 0.62
O55000	Ppp1r10	Serine/threonine-protein phosphatase 1 regulatory subunit 10 OS=Rattus norvegicus OX=10116 GN=Ppp1r10 PE=1 SV=1	1	1	92.8	22	↑ 0.76	→ 0.24
Q9WTR8	Phlpp1	PH domain leucine-rich repeat protein phosphatase 1 OS=Rattus norvegicus OX=10116 GN=Phlpp1 PE=1 SV=1	1	1	183.2	0	↑ 0.75	↑ 0.39
P70478	Apc	Adenomatous polyposis coli protein OS=Rattus norvegicus OX=10116 GN=Apc PE=1 SV=1	1	1	310.3	0	↑ 0.75	→ 0.24
O08700	Vps45	Vacuolar protein sorting-associated protein 45 OS=Rattus norvegicus OX=10116 GN=Vps45 PE=1 SV=1	6	12	64.9	96	↑ 0.75	→ 0.21
O35263	Pafah1b3	Platelet-activating factor acetylhydrolase IB subunit gamma OS=Rattus norvegicus OX=10116 GN=Pafah1b3 PE=2 SV=1	2	4	25.8	52	↑ 0.75	→ 0.14
O54715	Atp6ap1	V-type proton ATPase subunit S1 OS=Rattus norvegicus OX=10116 GN=Atp6ap1 PE=2 SV=1	1	6	51.1	121	↑ 0.74	↑ 0.55
Q6TXG9	Sfr1	Swi5-dependent recombination DNA repair protein 1 homolog OS=Rattus norvegicus OX=10116 GN=Sfr1 PE=2 SV=1	2	5	28.7	51	↑ 0.74	↑ 0.7
P15865	Hist1h1e	Histone H1.4 OS=Rattus norvegicus OX=10116 GN=Hist1h1e PE=1 SV=3	11	145	22	3464	↑ 0.68	↑ 0.45
P83565	Mrpl40	39S ribosomal protein L40, mitochondrial OS=Rattus norvegicus OX=10116 GN=Mrpl40 PE=1 SV=2	1	1	24.4	0	↑ 0.67	↑ 0.64
Q68FP2	Pon3	Serum paraoxonase/lactonase 3 OS=Rattus norvegicus OX=10116 GN=Pon3 PE=2 SV=1	6	54	39.4	849	↑ 0.67	↑ 0.49
P60815	Flvcr2	Feline leukemia virus subgroup C receptor-related protein 2 OS=Rattus norvegicus OX=10116 GN=Flvcr2 PE=2 SV=1	1	8	59.7	304	↑ 0.66	→ 0.31
Q4QQV3	Fam162a	Protein FAM162A OS=Rattus norvegicus OX=10116 GN=Fam162a PE=2 SV=1	2	3	17.8	29	↑ 0.65	↑ 0.57

P31016	Dlg4	Disks large homolog 4 OS=Rattus norvegicus OX=10116 GN=Dlg4 PE=1 SV=1	1	3	80.4	31	↑	0.65	↑	0.59
P02767	Ttr	Transthyretin OS=Rattus norvegicus OX=10116 GN=Ttr PE=1 SV=1	4	36	15.7	726	↑	0.64	↑	0.38
P16228	Ctse	Cathepsin E OS=Rattus norvegicus OX=10116 GN=Ctse PE=1 SV=3	2	4	43	29	↑	0.64	↑	0.69
Q5U2U2	Crkl	Crk-like protein OS=Rattus norvegicus OX=10116 GN=Crkl PE=1 SV=1	5	19	33.8	304	↑	0.63	→	0.32
Q62739	Rab3ip	Rab-3A-interacting protein OS=Rattus norvegicus OX=10116 GN=Rab3ip PE=1 SV=1	1	1	50.9	20	↑	0.62	→	0.28
Q9Z2L0	Vdac1	Voltage-dependent anion-selective channel protein 1 OS=Rattus norvegicus OX=10116 GN=Vdac1 PE=1 SV=4	8	58	30.7	1925	↑	0.62	↑	0.59
Q9JKA8	Hcn3	Potassium/sodium hyperpolarization-activated cyclic nucleotide-gated channel 3 OS=Rattus norvegicus OX=10116 GN=Hcn3 PE=1 SV=1	1	2	86.8	0	↑	0.62	→	0.37
P60192	Snapin	SNARE-associated protein Snapin OS=Rattus norvegicus OX=10116 GN=Snapin PE=1 SV=1	2	6	14.9	192	↑	0.61	↑	0.42
Q5XIA1	Ncln	Nicalin OS=Rattus norvegicus OX=10116 GN=Ncln PE=2 SV=1	2	3	63	77	↑	0.6	→	0.24
P70475	Myt1l	Myelin transcription factor 1-like protein OS=Rattus norvegicus OX=10116 GN=Myt1l PE=1 SV=3	1	1	132.8	25	↑	0.6	→	0.01
Q8CGV7	Thtpa	Thiamine-triphosphatase OS=Rattus norvegicus OX=10116 GN=Thtpa PE=2 SV=3	2	5	24.5	153	↑	0.59	↑	0.52
B0BN70	Smim7	Small integral membrane protein 7 OS=Rattus norvegicus OX=10116 GN=Smim7 PE=3 SV=1	1	1	8.6	0	↑	0.58	→	0.32
P15589	Sts	Steryl-sulfatase OS=Rattus norvegicus OX=10116 GN=Sts PE=1 SV=2	2	3	62.6	32	↑	0.58	→	0.12
P62083	Rps7	40S ribosomal protein S7 OS=Rattus norvegicus OX=10116 GN=Rps7 PE=1 SV=1	5	26	22.1	417	↑	0.58	↑	0.4
P19944	Rplp1	60S acidic ribosomal protein P1 OS=Rattus norvegicus OX=10116 GN=Rplp1 PE=3 SV=1	1	5	11.5	136	↑	0.57	→	0.25
P19234	Ndufv2	NADH dehydrogenase [ubiquinone] flavoprotein 2, mitochondrial OS=Rattus norvegicus OX=10116 GN=Ndufv2 PE=1 SV=2	3	12	27.4	98	↑	0.57	→	0.16
Q9Z1E1	Flot1	Flotillin-1 OS=Rattus norvegicus OX=10116 GN=Flot1 PE=2 SV=2	8	20	47.5	191	↑	0.56	→	0.29
P11240	Cox5a	Cytochrome c oxidase subunit 5A, mitochondrial OS=Rattus norvegicus OX=10116 GN=Cox5a PE=1 SV=1	4	18	16.1	223	↑	0.56	↑	0.5
Q9Z340	Pard3	Partitioning defective 3 homolog OS=Rattus norvegicus OX=10116 GN=Pard3 PE=1 SV=1	1	1	149.4	0	↑	0.55	↑	0.42
Q3ZU82	Golga5	Golgin subfamily A member 5 OS=Rattus norvegicus OX=10116 GN=Golga5 PE=1 SV=1	2	8	82.3	40	↑	0.55	↑	0.53
P61023	Chp1	Calcineurin B homologous protein 1 OS=Rattus norvegicus OX=10116 GN=Chp1 PE=1 SV=2	2	3	22.4	40	↑	0.54	→	0.35
P02634	S100g	Protein S100-G OS=Rattus norvegicus OX=10116 GN=S100g PE=1 SV=3	2	3	9	122	↑	0.54	→	-0.15
P08427	Sftpa1	Pulmonary surfactant-associated protein A OS=Rattus norvegicus OX=10116 GN=Sftpa1 PE=1 SV=3	3	50	26.3	1367	↑	0.54	→	0.16
Q99MC0	Ppp1r14a	Protein phosphatase 1 regulatory subunit 14A OS=Rattus norvegicus OX=10116 GN=Ppp1r14a PE=1 SV=1	3	14	16.7	236	↑	0.53	→	0.36
P84817	Fis1	Mitochondrial fission 1 protein OS=Rattus norvegicus OX=10116 GN=Fis1 PE=1 SV=1	1	12	17	229	↑	0.53	→	0.26
P01041	Cstb	Cystatin-B OS=Rattus norvegicus OX=10116 GN=Cstb PE=1 SV=1	3	26	11.2	427	↑	0.53	→	0.12
Q920D5	Casp12	Caspase-12 OS=Rattus norvegicus OX=10116 GN=Casp12 PE=1 SV=1	1	2	47.8	18	↑	0.53	→	0.29
P00406	Mtco2	Cytochrome c oxidase subunit 2 OS=Rattus norvegicus OX=10116 GN=Mtco2 PE=1 SV=3	6	28	25.9	222	↑	0.53	↑	0.67

Q6PCT3	Tpd52l2	Tumor protein D54 OS=Rattus norvegicus OX=10116 GN=Tpd52l2 PE=1 SV=1	6	25	24	500	↑	0.52	→	0.29
Q5M7W6	Fam234a	Protein FAM234A OS=Rattus norvegicus OX=10116 GN=Fam234a PE=1 SV=1	2	4	60.6	59	↑	0.52	↑	0.52
Q09073	Slc25a5	ADP/ATP translocase 2 OS=Rattus norvegicus OX=10116 GN=Slc25a5 PE=1 SV=3	6	99	32.9	1468	↑	0.52	↑	0.56
Q8K1P9	Fads3	Fatty acid desaturase 3 OS=Rattus norvegicus OX=10116 GN=Fads3 PE=1 SV=1	2	5	51.4	90	↑	0.52	→	0.32
Q07652	Cacna1e	Voltage-dependent R-type calcium channel subunit alpha-1E OS=Rattus norvegicus OX=10116 GN=Cacna1e PE=1 SV=1	1	3	252	0	↑	0.51	→	0.05
Q9ET64	Smpd2	Sphingomyelin phosphodiesterase 2 OS=Rattus norvegicus OX=10116 GN=Smpd2 PE=1 SV=1	1	1	47.6	21	↑	0.51	↑	0.53
P23680	Apcs	Serum amyloid P-component OS=Rattus norvegicus OX=10116 GN=Apcs PE=2 SV=2	3	7	26.2	32	↑	0.5	→	0.37
F1LQY6	Necab2	N-terminal EF-hand calcium-binding protein 2 OS=Rattus norvegicus OX=10116 GN=Necab2 PE=1 SV=3	1	1	43.5	0	↑	0.5	→	0.07
Q9WTR7	Sec11c	Signal peptidase complex catalytic subunit SEC11C OS=Rattus norvegicus OX=10116 GN=Sec11c PE=2 SV=3	1	2	21.6	28	↑	0.5	→	0.3
Q6MG62	Msh5	MutS protein homolog 5 OS=Rattus norvegicus OX=10116 GN=Msh5 PE=2 SV=1	1	4	92.4	29	↑	0.49	↑	0.55
P12075	Cox5b	Cytochrome c oxidase subunit 5B, mitochondrial OS=Rattus norvegicus OX=10116 GN=Cox5b PE=1 SV=2	1	6	13.9	47	↑	0.49	→	0.32
Q6PDU7	Atp5mg	ATP synthase subunit g, mitochondrial OS=Rattus norvegicus OX=10116 GN=Atp5mg PE=1 SV=2	3	16	11.4	424	↑	0.49	↑	0.46
Q63186	Eif2b4	Translation initiation factor eIF-2B subunit delta OS=Rattus norvegicus OX=10116 GN=Eif2b4 PE=2 SV=1	4	7	57.8	128	↑	0.49	→	0.09
P70531	Eef2k	Eukaryotic elongation factor 2 kinase OS=Rattus norvegicus OX=10116 GN=Eef2k PE=1 SV=1	3	8	81.4	74	↑	0.49	→	0.23
P11654	Nup210	Nuclear pore membrane glycoprotein 210 OS=Rattus norvegicus OX=10116 GN=Nup210 PE=1 SV=1	1	1	204	0	↑	0.48	→	-0.02
Q00495	Csf1r	Macrophage colony-stimulating factor 1 receptor OS=Rattus norvegicus OX=10116 GN=Csf1r PE=1 SV=1	2	3	109.2	68	↑	0.48	→	0.28
P43278	H1f0	Histone H1.0 OS=Rattus norvegicus OX=10116 GN=H1f0 PE=2 SV=2	2	6	20.9	98	↑	0.48	→	0.32
Q63072	Bst1	ADP-ribosyl cyclase/cyclic ADP-ribose hydrolase 2 OS=Rattus norvegicus OX=10116 GN=Bst1 PE=2 SV=1	2	3	35.1	37	↑	0.48	→	-0.05
Q06000	Lpl	Lipoprotein lipase OS=Rattus norvegicus OX=10116 GN=Lpl PE=1 SV=1	1	1	53	0	↑	0.48	↑	0.41
P09875	Ugt2b1	UDP-glucuronosyltransferase 2B1 OS=Rattus norvegicus OX=10116 GN=Ugt2b1 PE=2 SV=1	2	5	60.4	67	↑	0.48	→	0.04
Q498U4	Sarnp	SAP domain-containing ribonucleoprotein OS=Rattus norvegicus OX=10116 GN=Sarnp PE=1 SV=3	1	6	23.6	189	↑	0.48	→	0.36
P07483	Fabp3	Fatty acid-binding protein, heart OS=Rattus norvegicus OX=10116 GN=Fabp3 PE=1 SV=2	1	6	14.8	94	↑	0.48	→	0.29
Q66HG3	Cndp1	Beta-Ala-His dipeptidase OS=Rattus norvegicus OX=10116 GN=Cndp1 PE=1 SV=1	2	7	54.9	49	↑	0.48	→	0.2

Q920G0	Skap2	Src kinase-associated phosphoprotein 2 OS=Rattus norvegicus OX=10116 GN=Skap2 PE=1 SV=1	2	3	40.7	80	↑	0.48	→	0.11
F1M3G7	Akap13	A-kinase anchor protein 13 OS=Rattus norvegicus OX=10116 GN=Akap13 PE=1 SV=2	1	1	301.2	0	↑	0.48	→	0.22
Q9JHW1	Cpd	Carboxypeptidase D OS=Rattus norvegicus OX=10116 GN=Cpd PE=1 SV=2	2	5	152.5	0	↑	0.47	→	0.24
P0C865	Mapk7	Mitogen-activated protein kinase 7 OS=Rattus norvegicus OX=10116 GN=Mapk7 PE=1 SV=1	1	1	87.8	0	↑	0.47	→	0.32
P42346	Mtor	Serine/threonine-protein kinase mTOR OS=Rattus norvegicus OX=10116 GN=Mtor PE=1 SV=1	8	20	288.6	105	↑	0.47	↑	0.49
Q64350	Eif2b5	Translation initiation factor eIF-2B subunit epsilon OS=Rattus norvegicus OX=10116 GN=Eif2b5 PE=1 SV=2	4	10	80.2	116	↑	0.47	→	0.27
P06302	Ptma	Prothymosin alpha OS=Rattus norvegicus OX=10116 GN=Ptma PE=1 SV=2	1	13	12.4	459	↑	0.47	→	0.23
P49864	Gzmk	Granzyme K OS=Rattus norvegicus OX=10116 GN=Gzmk PE=1 SV=1	1	1	28.4	35	↑	0.47	→	0.34
Q5FVG2	Epb41l5	Band 4.1-like protein 5 OS=Rattus norvegicus OX=10116 GN=Epb41l5 PE=2 SV=2	3	8	81.7	47	↑	0.46	↑	0.51
Q56A18	Smarce1	SWI/SNF-related matrix-associated actin-dependent regulator of chromatin subfamily E member 1 OS=Rattus norvegicus OX=10116 GN=Smarce1 PE=1 SV=1	1	3	42.8	58	↑	0.46	→	0.36
B2GV38	Ubl4a	Ubiquitin-like protein 4A OS=Rattus norvegicus OX=10116 GN=Ubl4a PE=2 SV=1	1	1	17.8	31	↑	0.46	→	-0.04
Q7TQ16	Uqcrq	Cytochrome b-c1 complex subunit 8 OS=Rattus norvegicus OX=10116 GN=Uqcrq PE=3 SV=1	1	2	9.8	35	↑	0.46	↑	0.45
Q3KR86	Immt	MICOS complex subunit Mic60 (Fragment) OS=Rattus norvegicus OX=10116 GN=Immt PE=1 SV=1	9	19	67.1	329	↑	0.46	↑	0.42
P47875	Csrp1	Cysteine and glycine-rich protein 1 OS=Rattus norvegicus OX=10116 GN=Csrp1 PE=1 SV=2	8	88	20.6	1802	↑	0.45	↑	0.45
P60203	Plp1	Myelin proteolipid protein OS=Rattus norvegicus OX=10116 GN=Plp1 PE=1 SV=2	1	2	30.1	0	↑	0.45	↑	0.46
P70483	Strn	Striatin OS=Rattus norvegicus OX=10116 GN=Strn PE=1 SV=1	4	7	86.2	71	↑	0.45	→	0.18
Q4FZT0	Stoml2	Stomatin-like protein 2, mitochondrial OS=Rattus norvegicus OX=10116 GN=Stoml2 PE=1 SV=1	3	10	38.4	222	↑	0.45	↑	0.53
B0BN86	Tmem11	Transmembrane protein 11, mitochondrial OS=Rattus norvegicus OX=10116 GN=Tmem11 PE=2 SV=1	1	1	21.3	0	↑	0.45	↑	0.47
P63174	Rpl38	60S ribosomal protein L38 OS=Rattus norvegicus OX=10116 GN=Rpl38 PE=1 SV=2	2	4	8.2	78	↑	0.45	↑	0.77
F1LTR1	Wdr26	WD repeat-containing protein 26 OS=Rattus norvegicus OX=10116 GN=Wdr26 PE=3 SV=2	1	3	58.5	92	↑	0.45	→	-0.03
P62864	Fau	40S ribosomal protein S30 OS=Rattus norvegicus OX=10116 GN=Fau PE=1 SV=1	1	4	6.6	96	↑	0.45	↑	0.62
Q9Z2Q4	Mtr	Methionine synthase OS=Rattus norvegicus OX=10116 GN=Mtr PE=1 SV=1	1	3	139.1	0	↑	0.44	→	0.26
P16257	Tspo	Translocator protein OS=Rattus norvegicus OX=10116 GN=Tspo PE=1 SV=1	1	6	18.9	58	↑	0.44	↑	0.47
P29410	Ak2	Adenylate kinase 2, mitochondrial OS=Rattus norvegicus OX=10116 GN=Ak2 PE=2 SV=2	5	19	26.4	281	↑	0.44	→	0.16
Q5JCS6	Sipa1l2	Signal-induced proliferation-associated 1-like protein 2 OS=Rattus norvegicus OX=10116 GN=Sipa1l2 PE=1 SV=1	1	5	189.5	26	↑	0.44	→	0.35
Q99MY2	Nudt4	Diphosphoinositol polyphosphate phosphohydrolase 2 OS=Rattus norvegicus OX=10116 GN=Nudt4 PE=2 SV=1	2	3	20.1	33	↑	0.44	↑	0.38

Q923I8	Cry2	Cryptochrome-2 OS=Rattus norvegicus OX=10116 GN=Cry2 PE=1 SV=1	1	2	67.2	24	↑	0.43	→	0.14
O08557	Ddah1	N(G),N(G)-dimethylarginine dimethylaminohydrolase 1 OS=Rattus norvegicus OX=10116 GN=Ddah1 PE=1 SV=3	8	58	31.4	1091	↑	0.43	↑	0.39
Q9R0C9	Sigmar1	Sigma non-opioid intracellular receptor 1 OS=Rattus norvegicus OX=10116 GN=Sigmar1 PE=1 SV=1	1	2	25.3	0	↑	0.43	→	0.28
Q9WUJ3	Pde4dip	Myomegalin OS=Rattus norvegicus OX=10116 GN=Pde4dip PE=1 SV=1	1	1	261.9	0	↑	0.43	→	0.02
O35986	Zranb2	Zinc finger Ran-binding domain-containing protein 2 OS=Rattus norvegicus OX=10116 GN=Zranb2 PE=1 SV=2	1	1	37.3	39	↑	0.43	↑	0.58
Q4V8B7	Hsdl1	Inactive hydroxysteroid dehydrogenase-like protein 1 OS=Rattus norvegicus OX=10116 GN=Hsdl1 PE=2 SV=1	2	5	36.9	20	↑	0.43	→	0.17
E9PTT0	Zdhhc17	Palmitoyltransferase ZDHHC17 OS=Rattus norvegicus OX=10116 GN=Zdhhc17 PE=1 SV=1	1	2	71.3	82	↑	0.43	↑	0.77
P11951	Cox6c2	Cytochrome c oxidase subunit 6C-2 OS=Rattus norvegicus OX=10116 GN=Cox6c2 PE=1 SV=3	4	21	8.4	113	↑	0.43	↑	0.42
Q5RJZ1	Rtel1	Regulator of telomere elongation helicase 1 OS=Rattus norvegicus OX=10116 GN=Rtel1 PE=2 SV=2	1	3	141.7	19	↑	0.43	↓	-0.48
Q1HCL7	Nadk2	NAD kinase 2, mitochondrial OS=Rattus norvegicus OX=10116 GN=Nadk2 PE=1 SV=1	2	4	48.1	52	↑	0.43	↑	0.44
I6L9G5	Rcn3	Reticulocalbin-3 OS=Rattus norvegicus OX=10116 GN=Rcn3 PE=1 SV=1	1	2	37.9	29	↑	0.43	↑	0.39
P0C1Q3	Lpcat2	Lysophosphatidylcholine acyltransferase 2 OS=Rattus norvegicus OX=10116 GN=Lpcat2 PE=3 SV=1	1	1	59.8	0	↑	0.42	→	0.3
D3Z8L7	Rras	Ras-related protein R-Ras OS=Rattus norvegicus OX=10116 GN=Rras PE=1 SV=1	5	15	23.9	288	↑	0.42	↑	0.45
Q5XIC0	Eci2	Enoyl-CoA delta isomerase 2, mitochondrial OS=Rattus norvegicus OX=10116 GN=Eci2 PE=1 SV=1	2	9	43	199	↑	0.42	→	0.3
A0JPN2	Slc39a4	Zinc transporter ZIP4 OS=Rattus norvegicus OX=10116 GN=Slc39a4 PE=2 SV=1	2	9	71.1	322	↑	0.42	→	0.36
P62628	Dynlrb1	Dynein light chain roadblock-type 1 OS=Rattus norvegicus OX=10116 GN=Dynlrb1 PE=1 SV=3	2	8	11	91	↑	0.41	→	0.3
P40241	Cd9	CD9 antigen OS=Rattus norvegicus OX=10116 GN=Cd9 PE=1 SV=2	3	44	25.2	418	↑	0.41	↑	0.54
Q5HZB0	Tmem123	Porimin OS=Rattus norvegicus OX=10116 GN=Tmem123 PE=2 SV=1	1	3	20	38	↑	0.41	→	0.31
P61515	Rpl37a-ps1	Putative 60S ribosomal protein L37a OS=Rattus norvegicus OX=10116 GN=Rpl37a-ps1 PE=5 SV=2	1	2	10.3	133	↑	0.41	↑	0.47
P53812	Pitpnb	Phosphatidylinositol transfer protein beta isoform OS=Rattus norvegicus OX=10116 GN=Pitpnb PE=1 SV=2	3	14	31.4	146	↑	0.41	→	0.18
P11505	Atp2b1	Plasma membrane calcium-transporting ATPase 1 OS=Rattus norvegicus OX=10116 GN=Atp2b1 PE=1 SV=3	11	63	134.6	720	↑	0.41	→	0.3
Q64542	Atp2b4	Plasma membrane calcium-transporting ATPase 4 OS=Rattus norvegicus OX=10116 GN=Atp2b4 PE=1 SV=1	12	59	133	643	↑	0.41	→	0.35

P97521	Slc25a20	Mitochondrial carnitine/acylcarnitine carrier protein OS=Rattus norvegicus OX=10116 GN=Slc25a20 PE=1 SV=1	7	18	33.1	165	↑	0.41	↑	0.43
P62142	Ppp1cb	Serine/threonine-protein phosphatase PP1-beta catalytic subunit OS=Rattus norvegicus OX=10116 GN=Ppp1cb PE=1 SV=3	2	34	37.2	409	↑	0.41	→	0.1
Q5FVF1	Fra10ac1	Protein FRA10AC1 homolog OS=Rattus norvegicus OX=10116 GN=Fra10ac1 PE=1 SV=1	1	2	37.1	0	↑	0.4	→	0.21
P16573	Ceacam1	Carcinoembryonic antigen-related cell adhesion molecule 1 OS=Rattus norvegicus OX=10116 GN=Ceacam1 PE=1 SV=4	1	6	57.4	76	↑	0.4	→	0.2
P24485	Cd53	Leukocyte surface antigen CD53 OS=Rattus norvegicus OX=10116 GN=Cd53 PE=1 SV=3	1	2	24.2	30	↑	0.4	→	0.2
O55156	Clip2	CAP-Gly domain-containing linker protein 2 OS=Rattus norvegicus OX=10116 GN=Clip2 PE=1 SV=1	2	5	115.4	61	↑	0.4	↑	0.39
Q62638	Glg1	Golgi apparatus protein 1 OS=Rattus norvegicus OX=10116 GN=Glg1 PE=1 SV=1	4	8	133.5	64	↑	0.4	→	0.33
O35795	Entpd2	Ectonucleoside triphosphate diphosphohydrolase 2 OS=Rattus norvegicus OX=10116 GN=Entpd2 PE=1 SV=1	1	1	54.4	0	↑	0.4	→	-0.05
P07756	Cps1	Carbamoyl-phosphate synthase [ammonia], mitochondrial OS=Rattus norvegicus OX=10116 GN=Cps1 PE=1 SV=1	1	5	164.5	72	↑	0.4	↑	0.55
P35763	Prf1	Perforin-1 OS=Rattus norvegicus OX=10116 GN=Prf1 PE=2 SV=1	3	5	61.5	76	↑	0.4	→	0.28
P17178	Cyp27a1	Sterol 26-hydroxylase, mitochondrial OS=Rattus norvegicus OX=10116 GN=Cyp27a1 PE=1 SV=1	1	2	60.7	0	↑	0.4	→	0.22
G3V801	Prss12	Neurotrypsin OS=Rattus norvegicus OX=10116 GN=Prss12 PE=1 SV=1	2	3	84.2	0	↑	0.4	→	0.14
D3ZAF6	Atp5mf	ATP synthase subunit f, mitochondrial OS=Rattus norvegicus OX=10116 GN=Atp5mf PE=1 SV=1	3	25	10.4	417	↑	0.39	↑	0.39
P55053	Fabp5	Fatty acid-binding protein 5 OS=Rattus norvegicus OX=10116 GN=Fabp5 PE=1 SV=3	3	28	15.1	414	↑	0.39	→	0.22
Q9EPJ3	Mrps26	28S ribosomal protein S26, mitochondrial OS=Rattus norvegicus OX=10116 GN=Mrps26 PE=1 SV=1	1	1	23.3	0	↑	0.39	→	0.35
D3ZFJ3	Sh3bp1	SH3 domain-binding protein 1 OS=Rattus norvegicus OX=10116 GN=Sh3bp1 PE=1 SV=1	3	5	74.8	43	↑	0.39	→	-0.3
P14173	Ddc	Aromatic-L-amino-acid decarboxylase OS=Rattus norvegicus OX=10116 GN=Ddc PE=1 SV=1	3	11	54	104	↑	0.39	→	0.26
Q5RKJ1	Maea	E3 ubiquitin-protein transferase MAEA OS=Rattus norvegicus OX=10116 GN=Maea PE=2 SV=2	1	1	45.3	0	↑	0.39	→	0.16
P04550	Ptms	Parathymosin OS=Rattus norvegicus OX=10116 GN=Ptms PE=1 SV=2	2	14	11.6	531	↑	0.39	→	-0.01
P16970	Abcd3	ATP-binding cassette sub-family D member 3 OS=Rattus norvegicus OX=10116 GN=Abcd3 PE=1 SV=3	3	7	75.3	162	↑	0.39	↑	0.46
P04182	Oat	Ornithine aminotransferase, mitochondrial OS=Rattus norvegicus OX=10116 GN=Oat PE=1 SV=1	6	25	48.3	271	↑	0.39	→	0.04
Q0H8B9	Clec2d11	C-type lectin domain family 2 member D11 OS=Rattus norvegicus OX=10116 GN=Clec2d11 PE=1 SV=1	4	29	23.5	528	↑	0.38	↑	0.43

D3ZXD8	Tmem245	Transmembrane protein 245 OS=Rattus norvegicus OX=10116 GN=Tmem245 PE=1 SV=1	1	2	97.2	50	↑	0.38	↑	0.39
P47864	Aqp5	Aquaporin-5 OS=Rattus norvegicus OX=10116 GN=Aqp5 PE=2 SV=1	3	12	28.4	252	↑	0.38	↑	0.5
P11662	Mtnd2	NADH-ubiquinone oxidoreductase chain 2 OS=Rattus norvegicus OX=10116 GN=Mtnd2 PE=3 SV=3	1	2	38.6	19	↑	0.38	→	0.15
P81155	Vdac2	Voltage-dependent anion-selective channel protein 2 OS=Rattus norvegicus OX=10116 GN=Vdac2 PE=1 SV=2	7	36	31.7	459	↑	0.38	→	0.37
C9WPN6	Eif2s3y	Eukaryotic translation initiation factor 2 subunit 3, Y-linked OS=Rattus norvegicus OX=10116 GN=Eif2s3y PE=2 SV=2	1	22	51.1	475	↑	0.38	→	0.3
Q63487	RragB	Ras-related GTP-binding protein B OS=Rattus norvegicus OX=10116 GN=RragB PE=1 SV=1	3	5	43.2	153	↑	0.38	→	0.21
P00884	Aldob	Fructose-bisphosphate aldolase B OS=Rattus norvegicus OX=10116 GN=Aldob PE=1 SV=2	1	8	39.6	59	↑	0.38	→	0.33
P70541	Eif2b3	Translation initiation factor eIF-2B subunit gamma OS=Rattus norvegicus OX=10116 GN=Eif2b3 PE=2 SV=2	4	14	50.4	152	↑	0.38	→	0.13
P08426	Try3	Cationic trypsin-3 OS=Rattus norvegicus OX=10116 GN=Try3 PE=2 SV=1	3	9	26.3	44	↑	0.38	→	0.31
P48675	Des	Desmin OS=Rattus norvegicus OX=10116 GN=Des PE=1 SV=2	14	117	53.4	1746	↑	0.38	↑	0.67
Q9EPT8	Clic5	Chloride intracellular channel protein 5 OS=Rattus norvegicus OX=10116 GN=Clic5 PE=1 SV=1	9	96	28.3	1831	→	0.37	↑	0.39
Q5M821	Ppm1h	Protein phosphatase 1H OS=Rattus norvegicus OX=10116 GN=Ppm1h PE=1 SV=2	1	2	56.3	53	→	0.37	↑	0.45
P29418	Atp5f1e	ATP synthase subunit epsilon, mitochondrial OS=Rattus norvegicus OX=10116 GN=Atp5f1e PE=1 SV=2	2	12	5.8	46	→	0.37	↑	0.4
P70709	Rnase3	Eosinophil cationic protein OS=Rattus norvegicus OX=10116 GN=Rnase3 PE=2 SV=1	2	3	18	17	→	0.37	↑	0.51
Q8VGC3	Cacnb2	Voltage-dependent L-type calcium channel subunit beta-2 OS=Rattus norvegicus OX=10116 GN=Cacnb2 PE=1 SV=2	1	2	73.2	35	→	0.37	↑	0.39
Q5XI96	Rnaseh2b	Ribonuclease H2 subunit B OS=Rattus norvegicus OX=10116 GN=Rnaseh2b PE=2 SV=1	1	1	34.6	31	→	0.37	↑	0.56
Q63246	Foxc2	Forkhead box protein C2 OS=Rattus norvegicus OX=10116 GN=Foxc2 PE=1 SV=2	1	1	52.8	0	→	0.36	↑	0.4
Q9ESS6	Bcam	Basal cell adhesion molecule OS=Rattus norvegicus OX=10116 GN=Bcam PE=2 SV=1	13	93	67.5	1384	→	0.36	↑	0.42
P02650	Apoe	Apolipoprotein E OS=Rattus norvegicus OX=10116 GN=Apoe PE=1 SV=2	15	125	35.7	1582	→	0.36	↑	0.68
P05508	Mtnd4	NADH-ubiquinone oxidoreductase chain 4 OS=Rattus norvegicus OX=10116 GN=Mtnd4 PE=3 SV=3	1	5	51.7	144	→	0.35	↑	0.63
F1LQ70	Alox12	Arachidonate 12-lipoxygenase, 12S-type OS=Rattus norvegicus OX=10116 GN=Alox12 PE=1 SV=1	4	10	75.5	98	→	0.35	↑	0.47
Q9JI92	Sdcbp	Syntenin-1 OS=Rattus norvegicus OX=10116 GN=Sdcbp PE=1 SV=1	1	5	32.4	35	→	0.35	↑	0.48
Q91XU1	Qki	Protein quaking OS=Rattus norvegicus OX=10116 GN=Qki PE=1 SV=2	5	18	37.6	177	→	0.34	↑	0.45
O35786	Cmklr1	Chemokine-like receptor 1 OS=Rattus norvegicus OX=10116 GN=Cmklr1 PE=1 SV=1	1	7	41.7	30	→	0.34	↑	0.39
Q99P39	Nfs1	Cysteine desulfurase, mitochondrial OS=Rattus norvegicus OX=10116 GN=Nfs1 PE=2 SV=1	3	6	50	97	→	0.34	↑	0.38
P18614	Itga1	Integrin alpha-1 OS=Rattus norvegicus OX=10116 GN=Itga1 PE=1 SV=1	17	91	130.7	1386	→	0.33	↑	0.4
Q5PQM2	Klc4	Kinesin light chain 4 OS=Rattus norvegicus OX=10116 GN=Klc4 PE=1 SV=1	6	14	68.9	227	→	0.33	↑	0.38

Q63400	Cldn3	Claudin-3 OS=Rattus norvegicus OX=10116 GN=Cldn3 PE=1 SV=2	2	7	23.3	92	→	0.32	↑	0.52
Q4KLL0	Tcea1	Transcription elongation factor A protein 1 OS=Rattus norvegicus OX=10116 GN=Tcea1 PE=1 SV=1	3	4	33.9	78	→	0.32	↑	0.42
Q5PPN7	Ccdc51	Coiled-coil domain-containing protein 51 OS=Rattus norvegicus OX=10116 GN=Ccdc51 PE=2 SV=2	1	5	45.8	24	→	0.3	↑	0.64
Q6P9Z6	Tacstd2	Tumor-associated calcium signal transducer 2 OS=Rattus norvegicus OX=10116 GN=Tacstd2 PE=2 SV=1	3	6	35.5	38	→	0.3	↑	0.54
Q6GQP4	Rab31	Ras-related protein Rab-31 OS=Rattus norvegicus OX=10116 GN=Rab31 PE=1 SV=2	3	5	21.4	106	→	0.28	↑	0.41
Q6AXY3	Tex29	Testis-expressed protein 29 OS=Rattus norvegicus OX=10116 GN=Tex29 PE=2 SV=1	1	5	19.9	23	→	0.28	↑	0.43
Q6VEU1	Nob1	RNA-binding protein NOB1 OS=Rattus norvegicus OX=10116 GN=Nob1 PE=2 SV=1	1	1	46.4	16	→	0.28	↑	0.52
O88994	43526	Mitochondrial amidoxime reducing component 2 OS=Rattus norvegicus OX=10116 GN=Marc2 PE=2 SV=1	4	6	38.2	71	→	0.28	↑	0.45
Q5QE78	Aox2	Aldehyde oxidase 2 OS=Rattus norvegicus OX=10116 GN=Aox2 PE=2 SV=1	1	5	147.8	43	→	0.27	↑	0.4
Q6AXS5	Serbp1	Plasminogen activator inhibitor 1 RNA-binding protein OS=Rattus norvegicus OX=10116 GN=Serbp1 PE=1 SV=2	2	6	44.7	171	→	0.25	↑	0.5
F1LYQ8	Farp1	FERM, ARHGEF and pleckstrin domain-containing protein 1 OS=Rattus norvegicus OX=10116 GN=Farp1 PE=1 SV=2	5	13	118.8	171	→	0.24	↑	0.47
Q5FVQ8	Nlrx1	NLR family member X1 OS=Rattus norvegicus OX=10116 GN=Nlrx1 PE=2 SV=1	1	2	107.5	21	→	0.24	↑	0.49
O54889	Polr1a	DNA-directed RNA polymerase I subunit RPA1 OS=Rattus norvegicus OX=10116 GN=Polr1a PE=1 SV=1	1	2	194.1	0	→	0.24	↑	0.39
Q08464	Fzd2	Frizzled-2 OS=Rattus norvegicus OX=10116 GN=Fzd2 PE=1 SV=1	2	3	63.8	16	→	0.23	↑	0.64
P06341	RT1-B	Rano class II histocompatibility antigen, A beta chain (Fragment) OS=Rattus norvegicus OX=10116 GN=RT1-B PE=3 SV=1	2	16	26.8	174	→	0.22	↑	0.44
A1A5S1	Prpf6	Pre-mRNA-processing factor 6 OS=Rattus norvegicus OX=10116 GN=Prpf6 PE=1 SV=1	6	14	106.7	132	→	0.21	↑	0.38
Q80VJ4	Gpcpd1	Glycerophosphocholine phosphodiesterase GPCPD1 OS=Rattus norvegicus OX=10116 GN=Gpcpd1 PE=1 SV=1	3	4	76.2	19	→	0.2	↑	0.43
D3ZAR1	Ldlrap1	Low density lipoprotein receptor adapter protein 1 OS=Rattus norvegicus OX=10116 GN=Ldlrap1 PE=1 SV=2	1	2	33.8	15	→	0.2	↑	0.48
Q02353	Ndst1	Bifunctional heparan sulfate N-deacetylase/N-sulfotransferase 1 OS=Rattus norvegicus OX=10116 GN=Ndst1 PE=1 SV=1	1	1	100.7	28	→	0.18	↑	0.53
Q8VHK0	Acot8	Acyl-coenzyme A thioesterase 8 OS=Rattus norvegicus OX=10116 GN=Acot8 PE=1 SV=1	1	1	36	21	→	0.18	↓	-0.65
P62832	Rpl23	60S ribosomal protein L23 OS=Rattus norvegicus OX=10116 GN=Rpl23 PE=2 SV=1	6	35	14.9	659	→	0.16	↑	0.38
P60570	Panx1	Pannexin-1 OS=Rattus norvegicus OX=10116 GN=Panx1 PE=1 SV=1	1	2	48	0	→	0.13	↑	0.42
Q6AYI5	Shoc2	Leucine-rich repeat protein SHOC-2 OS=Rattus norvegicus OX=10116 GN=Shoc2 PE=2 SV=1	1	1	64.9	0	→	0.13	↑	0.47
P17988	Sult1a1	Sulfotransferase 1A1 OS=Rattus norvegicus OX=10116 GN=Sult1a1 PE=1 SV=1	7	21	33.9	147	→	0.12	↑	0.52
P27274	Cd59	CD59 glycoprotein OS=Rattus norvegicus OX=10116 GN=Cd59 PE=1 SV=2	2	3	13.8	24	→	0.09	↑	0.44

P18589	Mx2	Interferon-induced GTP-binding protein Mx2 OS=Rattus norvegicus OX=10116 GN=Mx2 PE=2 SV=1	1	1	75	25	→	0.09	↑	0.38
O88267	Acot1	Acyl-coenzyme A thioesterase 1 OS=Rattus norvegicus OX=10116 GN=Acot1 PE=1 SV=1	1	26	46	504	→	0.05	↑	0.53
Q499N3	Wdr18	WD repeat-containing protein 18 OS=Rattus norvegicus OX=10116 GN=Wdr18 PE=2 SV=1	2	3	47.2	53	→	0.04	↑	0.49
P21704	Dnase1	Deoxyribonuclease-1 OS=Rattus norvegicus OX=10116 GN=Dnase1 PE=2 SV=1	1	3	32	24	→	0.03	↑	0.45
Q6AYT0	Cryz	Quinone oxidoreductase OS=Rattus norvegicus OX=10116 GN=Cryz PE=2 SV=1	2	2	35	0	→	0.01	↑	0.44
P20717	Padi2	Protein-arginine deiminase type-2 OS=Rattus norvegicus OX=10116 GN=Padi2 PE=1 SV=1	1	1	75.3	22	→	0.01	↓	-0.51
Q03555	Gphn	Gephyrin OS=Rattus norvegicus OX=10116 GN=Gphn PE=1 SV=3	1	1	83.2	25	→	-0.01	↓	-0.43
Q64716	Insrr	Insulin receptor-related protein OS=Rattus norvegicus OX=10116 GN=Insrr PE=1 SV=3	1	2	144.8	0	→	-0.05	↑	0.73
Q63089	Slc22a1	Solute carrier family 22 member 1 OS=Rattus norvegicus OX=10116 GN=Slc22a1 PE=1 SV=1	1	1	61.5	0	→	-0.07	↑	0.43
Q9ES71	Gnpat	Dihydroxyacetone phosphate acyltransferase OS=Rattus norvegicus OX=10116 GN=Gnpat PE=1 SV=1	3	7	77	88	→	-0.09	↑	0.4
P62275	Rps29	40S ribosomal protein S29 OS=Rattus norvegicus OX=10116 GN=Rps29 PE=1 SV=2	1	1	6.7	22	→	-0.09	↑	0.38
Q9JK72	Ccs	Copper chaperone for superoxide dismutase OS=Rattus norvegicus OX=10116 GN=Ccs PE=1 SV=1	3	9	28.9	195	→	-0.11	↓	-0.46
Q9JKL7	Srek1	Splicing regulatory glutamine/lysine-rich protein 1 OS=Rattus norvegicus OX=10116 GN=Srek1 PE=1 SV=1	1	1	56.8	35	→	-0.12	↓	-0.5
Q6P6U0	Fgr	Tyrosine-protein kinase Fgr OS=Rattus norvegicus OX=10116 GN=Fgr PE=1 SV=1	2	18	58.8	145	→	-0.12	↓	-0.52
P86410	Ralgapb	Ral GTPase-activating protein subunit beta OS=Rattus norvegicus OX=10116 GN=Ralgapb PE=1 SV=1	3	4	165.3	40	→	-0.13	↓	-0.48
P49816	Tsc2	Tuberin OS=Rattus norvegicus OX=10116 GN=Tsc2 PE=1 SV=1	1	1	201.2	0	→	-0.13	↓	-0.43
P35467	S100a1	Protein S100-A1 OS=Rattus norvegicus OX=10116 GN=S100a1 PE=1 SV=3	1	3	10.6	48	→	-0.14	↓	-0.48
Q64294	Pdpn	Podoplanin OS=Rattus norvegicus OX=10116 GN=Pdpn PE=1 SV=1	1	2	17.6	0	→	-0.14	↓	-0.42
P15800	Lamb2	Laminin subunit beta-2 OS=Rattus norvegicus OX=10116 GN=Lamb2 PE=1 SV=1	8	14	196.3	65	→	-0.14	↓	-0.5
Q6IRI9	Fmo2	Dimethylaniline monooxygenase [N-oxide-forming] 2 OS=Rattus norvegicus OX=10116 GN=Fmo2 PE=2 SV=3	4	23	60.9	204	→	-0.15	↑	2.05
Q62640	Grid1	Glutamate receptor ionotropic, delta-1 OS=Rattus norvegicus OX=10116 GN=Grid1 PE=2 SV=1	1	1	112.1	18	→	-0.15	↑	0.42
P06762	Hmox1	Heme oxygenase 1 OS=Rattus norvegicus OX=10116 GN=Hmox1 PE=1 SV=1	1	1	33	0	→	-0.16	↓	-0.39
B1WC88		UPF0729 protein C18orf32 homolog OS=Rattus norvegicus OX=10116 PE=3 SV=1	1	2	8.2	62	→	-0.18	↓	-0.44
Q68FX7	Thoc5	THO complex subunit 5 homolog OS=Rattus norvegicus OX=10116 GN=Thoc5 PE=2 SV=1	1	1	78.6	30	→	-0.19	↑	0.39
P27139	Ca2	Carbonic anhydrase 2 OS=Rattus norvegicus OX=10116 GN=Ca2 PE=1 SV=2	10	148	29.1	2980	→	-0.19	↓	-0.41
Q5M823	Nudcd2	NudC domain-containing protein 2 OS=Rattus norvegicus OX=10116 GN=Nudcd2 PE=2 SV=1	1	1	17.7	0	→	-0.2	↓	-0.67
Q8R431	Mgll	Monoglyceride lipase OS=Rattus norvegicus OX=10116 GN=Mgll PE=1 SV=1	4	11	33.5	154	→	-0.2	↓	-0.43

Q7TNK6	Trmt11	tRNA (guanine(10)-N2)-methyltransferase homolog OS=Rattus norvegicus OX=10116 GN=Trmt11 PE=2 SV=1	1	3	53.1	77	→	-0.21	↓	-0.39
P18418	Calr	Calreticulin OS=Rattus norvegicus OX=10116 GN=Calr PE=1 SV=1	9	100	48	1043	→	-0.21	↓	-0.4
O08697	Arl2	ADP-ribosylation factor-like protein 2 OS=Rattus norvegicus OX=10116 GN=Arl2 PE=1 SV=1	3	10	20.8	220	→	-0.22	↓	-0.65
Q9Z2Q7	Stx8	Syntaxin-8 OS=Rattus norvegicus OX=10116 GN=Stx8 PE=1 SV=1	1	6	26.9	0	→	-0.23	↓	-0.44
P54275	Msh2	DNA mismatch repair protein Msh2 OS=Rattus norvegicus OX=10116 GN=Msh2 PE=2 SV=1	1	7	104	0	→	-0.24	↓	-0.62
B3DMA0	Tp53i11	Tumor protein p53-inducible protein 11 OS=Rattus norvegicus OX=10116 GN=Tp53i11 PE=1 SV=1	2	4	20.9	154	→	-0.24	↓	-0.38
P62329	Tmsb4x	Thymosin beta-4 OS=Rattus norvegicus OX=10116 GN=Tmsb4x PE=1 SV=2	3	35	5.1	287	→	-0.24	↓	-0.45
B1H268	Mcmbp	Mini-chromosome maintenance complex-binding protein OS=Rattus norvegicus OX=10116 GN=Mcmbp PE=2 SV=1	1	1	73	0	→	-0.25	↓	-0.5
P25809	Ckmt1	Creatine kinase U-type, mitochondrial OS=Rattus norvegicus OX=10116 GN=Ckmt1 PE=1 SV=1	4	22	47	174	→	-0.25	↓	-0.39
Q66HF9	Lrrfip1	Leucine-rich repeat flightless-interacting protein 1 OS=Rattus norvegicus OX=10116 GN=Lrrfip1 PE=1 SV=1	5	11	80	65	→	-0.26	↓	-0.43
Q6AYF1	Mapkap1	Target of rapamycin complex 2 subunit MAPKAP1 OS=Rattus norvegicus OX=10116 GN=Mapkap1 PE=2 SV=1	1	1	59	0	→	-0.27	↓	-0.53
Q5RKI3	Poll	DNA polymerase lambda OS=Rattus norvegicus OX=10116 GN=Poll PE=2 SV=1	1	5	62.4	33	→	-0.28	↓	-0.87
P09650	Mcpt1	Mast cell protease 1 OS=Rattus norvegicus OX=10116 GN=Mcpt1 PE=1 SV=3	3	8	28.6	62	→	-0.28	↓	-0.51
Q8R508	Fat3	Protocadherin Fat 3 OS=Rattus norvegicus OX=10116 GN=Fat3 PE=1 SV=1	1	1	501.8	0	→	-0.28	↓	-0.48
P82450	Siae	Sialate O-acetylesterase OS=Rattus norvegicus OX=10116 GN=Siae PE=1 SV=2	2	4	60.4	38	→	-0.3	↓	-0.52
Q8K4G6	Macrod1	ADP-ribose glycohydrolase MACROD1 (Fragment) OS=Rattus norvegicus OX=10116 GN=Macrod1 PE=2 SV=2	1	2	28.6	68	→	-0.31	↓	-0.42
P18437	Hmgn2	Non-histone chromosomal protein HMG-17 OS=Rattus norvegicus OX=10116 GN=Hmgn2 PE=1 SV=2	2	15	9.4	85	→	-0.32	↓	-0.58
Q7TNY6	Acbd3	Golgi resident protein GCP60 OS=Rattus norvegicus OX=10116 GN=Acbd3 PE=1 SV=3	4	8	60.4	70	→	-0.33	↓	-0.43
Q5BJK8	Golim4	Golgi integral membrane protein 4 OS=Rattus norvegicus OX=10116 GN=Golim4 PE=1 SV=2	1	1	76.6	0	→	-0.35	↓	-0.41
P09006	Serpina3n	Serine protease inhibitor A3N OS=Rattus norvegicus OX=10116 GN=Serpina3n PE=1 SV=3	11	60	46.6	971	→	-0.37	↑	0.5
B5DF91	Elavl1	ELAV-like protein 1 OS=Rattus norvegicus OX=10116 GN=Elavl1 PE=1 SV=1	7	31	36.1	501	→	-0.37	↓	-0.5
Q63610	Tpm3	Tropomyosin alpha-3 chain OS=Rattus norvegicus OX=10116 GN=Tpm3 PE=1 SV=2	9	92	29	1334	→	-0.37	↓	-0.38
Q6AY19	Coq8b	Atypical kinase COQ8B, mitochondrial OS=Rattus norvegicus OX=10116 GN=Coq8b PE=1 SV=1	2	5	58.9	33	→	-0.37	↓	-0.57
P18163	Acsl1	Long-chain-fatty-acid--CoA ligase 1 OS=Rattus norvegicus OX=10116 GN=Acsl1 PE=1 SV=1	10	58	78.1	612	→	-0.37	↓	-0.43
P14480	Fgb	Fibrinogen beta chain OS=Rattus norvegicus OX=10116 GN=Fgb PE=1 SV=4	17	142	54.2	1704	↓	-0.38	→	-0.08
Q8VHV7	Hnrnph1	Heterogeneous nuclear ribonucleoprotein H OS=Rattus norvegicus OX=10116 GN=Hnrnph1 PE=1 SV=2	4	36	49.2	784	↓	-0.38	→	-0.27

P14046	A1i3	Alpha-1-inhibitor 3 OS=Rattus norvegicus OX=10116 GN=A1i3 PE=1 SV=1	10	671	163.7	11625	↓	-0.38	→	-0.16
Q1AAU6	Asap1	Arf-GAP with SH3 domain, ANK repeat and PH domain-containing protein 1 OS=Rattus norvegicus OX=10116 GN=Asap1 PE=1 SV=2	3	4	127	117	↓	-0.38	→	-0.14
P27881	Hk2	Hexokinase-2 OS=Rattus norvegicus OX=10116 GN=Hk2 PE=1 SV=1	3	11	102.5	161	↓	-0.38	↓	-0.71
Q792H5	Celf2	CUGBP Elav-like family member 2 OS=Rattus norvegicus OX=10116 GN=Celf2 PE=2 SV=1	1	4	54.2	48	↓	-0.39	→	-0.13
Q6URK4	Hnrnpa3	Heterogeneous nuclear ribonucleoprotein A3 OS=Rattus norvegicus OX=10116 GN=Hnrnpa3 PE=1 SV=1	7	43	39.6	737	↓	-0.4	→	-0.22
Q6IMY8	Hnrnpu	Heterogeneous nuclear ribonucleoprotein U OS=Rattus norvegicus OX=10116 GN=Hnrnpu PE=1 SV=1	16	155	87.7	2179	↓	-0.4	→	-0.29
F1LQ48	Hnrnpl	Heterogeneous nuclear ribonucleoprotein L OS=Rattus norvegicus OX=10116 GN=Hnrnpl PE=1 SV=2	13	85	67.9	1283	↓	-0.4	→	-0.19
Q562C9	Adi1	1,2-dihydroxy-3-keto-5-methylthiopentene dioxygenase OS=Rattus norvegicus OX=10116 GN=Adi1 PE=2 SV=1	1	1	21.4	0	↓	-0.4	→	-0.24
D4A1J4	Bdh2	3-hydroxybutyrate dehydrogenase type 2 OS=Rattus norvegicus OX=10116 GN=Bdh2 PE=3 SV=2	1	5	26.6	95	↓	-0.41	→	0.02
B0BN49	Rbmx2	RNA-binding motif protein, X-linked 2 OS=Rattus norvegicus OX=10116 GN=Rbmx2 PE=2 SV=1	1	2	37.8	0	↓	-0.41	→	-0.13
Q5M9G1	Hexim1	Protein HEXIM1 OS=Rattus norvegicus OX=10116 GN=Hexim1 PE=1 SV=1	1	2	40.3	0	↓	-0.42	→	-0.22
P00762	Prss1	Anionic trypsin-1 OS=Rattus norvegicus OX=10116 GN=Prss1 PE=1 SV=1	1	15	25.9	140	↓	-0.43	→	-0.37
P15083	Pigr	Polymeric immunoglobulin receptor OS=Rattus norvegicus OX=10116 GN=Pigr PE=1 SV=1	8	27	84.7	319	↓	-0.43	→	-0.22
Q3SWU3	Hnrnpdl	Heterogeneous nuclear ribonucleoprotein D-like OS=Rattus norvegicus OX=10116 GN=Hnrnpdl PE=1 SV=1	3	7	35.3	114	↓	-0.43	→	-0.29
Q6AYK1	Rnps1	RNA-binding protein with serine-rich domain 1 OS=Rattus norvegicus OX=10116 GN=Rnps1 PE=2 SV=1	1	2	34.2	0	↓	-0.43	↓	-0.54
P36876	Ppp2r2a	Serine/threonine-protein phosphatase 2A 55 kDa regulatory subunit B alpha isoform OS=Rattus norvegicus OX=10116 GN=Ppp2r2a PE=2 SV=1	2	19	51.6	143	↓	-0.43	↓	-0.6
Q03626	Mug1	Murinoglobulin-1 OS=Rattus norvegicus OX=10116 GN=Mug1 PE=2 SV=1	8	561	165.2	9728	↓	-0.43	→	0.01
Q05175	Basp1	Brain acid soluble protein 1 OS=Rattus norvegicus OX=10116 GN=Basp1 PE=1 SV=2	3	9	21.8	60	↓	-0.44	↓	-0.72
Q63416	Itih3	Inter-alpha-trypsin inhibitor heavy chain H3 OS=Rattus norvegicus OX=10116 GN=Itih3 PE=2 SV=1	12	86	99	1229	↓	-0.44	→	0.02
P21263	Nes	Nestin OS=Rattus norvegicus OX=10116 GN=Nes PE=1 SV=2	1	2	208.7	0	↓	-0.44	→	-0.24
Q4V8K5	Brox	BRO1 domain-containing protein BROX OS=Rattus norvegicus OX=10116 GN=Brox PE=2 SV=1	3	6	46.2	123	↓	-0.45	↓	-0.43
P47967	Lgals5	Galectin-5 OS=Rattus norvegicus OX=10116 GN=Lgals5 PE=1 SV=2	4	23	16.2	350	↓	-0.45	↓	-1.13
Q63041	A1m	Alpha-1-macroglobulin OS=Rattus norvegicus OX=10116 GN=A1m PE=1 SV=1	41	529	167	9699	↓	-0.46	→	0.17

Q9WTV0	Preb	Prolactin regulatory element-binding protein OS=Rattus norvegicus OX=10116 GN=Preb PE=1 SV=2	2	10	45.3	112	↓	-0.46	→	-0.12
P10687	Plcb1	1-phosphatidylinositol 4,5-bisphosphate phosphodiesterase beta-1 OS=Rattus norvegicus OX=10116 GN=Plcb1 PE=1 SV=1	3	8	138.3	52	↓	-0.46	→	-0.25
O08651	Phgdh	D-3-phosphoglycerate dehydrogenase OS=Rattus norvegicus OX=10116 GN=Phgdh PE=1 SV=3	1	5	56.5	132	↓	-0.47	→	-0.15
G3V9R8	Hnrnpc	Heterogeneous nuclear ribonucleoprotein C OS=Rattus norvegicus OX=10116 GN=Hnrnpc PE=1 SV=2	13	64	32.8	824	↓	-0.48	→	-0.3
Q9WTV1	Chi3l1	Chitinase-3-like protein 1 OS=Rattus norvegicus OX=10116 GN=Chi3l1 PE=2 SV=3	4	7	42.4	48	↓	-0.5	→	-0.22
Q5XIM4	Dmac2l	ATP synthase subunit s, mitochondrial OS=Rattus norvegicus OX=10116 GN=Dmac2l PE=2 SV=1	1	1	23.3	0	↓	-0.51	↓	-0.77
Q9QVC8	Fkbp4	Peptidyl-prolyl cis-trans isomerase FKBP4 OS=Rattus norvegicus OX=10116 GN=Fkbp4 PE=1 SV=3	10	28	51.4	310	↓	-0.51	↓	-0.5
Q6AYJ1	Recql	ATP-dependent DNA helicase Q1 OS=Rattus norvegicus OX=10116 GN=Recql PE=1 SV=1	1	1	69.6	29	↓	-0.51	↓	-0.51
Q6AY80	Nqo2	Ribosyldihydronicotinamide dehydrogenase [quinone] OS=Rattus norvegicus OX=10116 GN=Nqo2 PE=1 SV=3	5	17	26.3	244	↓	-0.51	→	-0.25
P60123	Ruvbl1	RuvB-like 1 OS=Rattus norvegicus OX=10116 GN=Ruvbl1 PE=1 SV=1	9	30	50.2	575	↓	-0.52	↓	-0.38
Q9QZ76	Mb	Myoglobin OS=Rattus norvegicus OX=10116 GN=Mb PE=1 SV=3	3	15	17.1	287	↓	-0.52	↓	-0.51
P97924	Kalrn	Kalirin OS=Rattus norvegicus OX=10116 GN=Kalrn PE=1 SV=3	1	7	336.4	28	↓	-0.53	↓	-0.43
B0BNK9	Cracr2b	EF-hand calcium-binding domain-containing protein 4A OS=Rattus norvegicus OX=10116 GN=Cracr2b PE=2 SV=1	1	1	44.5	0	↓	-0.54	→	0.2
P06866	Hp	Haptoglobin OS=Rattus norvegicus OX=10116 GN=Hp PE=1 SV=3	14	70	38.5	1188	↓	-0.55	↓	-0.38
Q6IFW6	Krt10	Keratin, type I cytoskeletal 10 OS=Rattus norvegicus OX=10116 GN=Krt10 PE=3 SV=1	10	104	56.5	1258	↓	-0.55	↓	-0.69
P00770	Mcpt2	Mast cell protease 2 OS=Rattus norvegicus OX=10116 GN=Mcpt2 PE=1 SV=1	1	3	27.1	27	↓	-0.55	→	0.13
A7VJC2	Hnrnpa2b1	Heterogeneous nuclear ribonucleoproteins A2/B1 OS=Rattus norvegicus OX=10116 GN=Hnrnpa2b1 PE=1 SV=1	17	204	37.5	3544	↓	-0.56	→	-0.34
Q3KRC5	Dus3l	tRNA-dihydrouridine(47) synthase [NAD(P)(+)]-like OS=Rattus norvegicus OX=10116 GN=Dus3l PE=2 SV=1	1	2	71.5	37	↓	-0.56	→	-0.26
Q63189	Prg2	Bone marrow proteoglycan OS=Rattus norvegicus OX=10116 GN=Prg2 PE=2 SV=1	1	2	25.1	68	↓	-0.58	↓	-0.88
Q63515	C4bpb	C4b-binding protein beta chain OS=Rattus norvegicus OX=10116 GN=C4bpb PE=2 SV=1	1	1	28.6	23	↓	-0.58	→	-0.33
Q6IFU7	Krt42	Keratin, type I cytoskeletal 42 OS=Rattus norvegicus OX=10116 GN=Krt42 PE=3 SV=1	3	126	50.2	1447	↓	-0.59	→	0.15
Q8CHN6	Sgpl1	Sphingosine-1-phosphate lyase 1 OS=Rattus norvegicus OX=10116 GN=Sgpl1 PE=2 SV=1	2	9	63.7	125	↓	-0.61	→	-0.3
P04256	Hnrnpa1	Heterogeneous nuclear ribonucleoprotein A1 OS=Rattus norvegicus OX=10116 GN=Hnrnpa1 PE=1 SV=3	3	53	34.2	659	↓	-0.62	↓	-0.49
P08650	C5	Complement C5 (Fragment) OS=Rattus norvegicus OX=10116 GN=C5 PE=1 SV=2	1	4	9	51	↓	-0.63	→	-0.11
P16290	Pgam2	Phosphoglycerate mutase 2 OS=Rattus norvegicus OX=10116 GN=Pgam2 PE=1 SV=2	3	28	28.7	592	↓	-0.64	↓	-0.54

Q64578	Atp2a1	Sarcoplasmic/endoplasmic reticulum calcium ATPase 1 OS=Rattus norvegicus OX=10116 GN=Atp2a1 PE=1 SV=1	3	58	109.3	723	↓	-0.66	↓	-0.94
P08649	C4	Complement C4 OS=Rattus norvegicus OX=10116 GN=C4 PE=1 SV=3	44	220	192	3558	↓	-0.68	→	-0.12
P09117	Aldoc	Fructose-bisphosphate aldolase C OS=Rattus norvegicus OX=10116 GN=Aldoc PE=1 SV=3	3	31	39.3	189	↓	-0.68	↓	-0.52
Q32PX2	Aimp2	Aminoacyl tRNA synthase complex-interacting multifunctional protein 2 OS=Rattus norvegicus OX=10116 GN=Aimp2 PE=2 SV=1	3	13	35.4	115	↓	-0.71	↓	-0.56
Q04931	Ssrp1	FACT complex subunit SSRP1 OS=Rattus norvegicus OX=10116 GN=Ssrp1 PE=1 SV=2	1	1	80.9	0	↓	-0.71	→	0.03
P31721	C1qb	Complement C1q subcomponent subunit B OS=Rattus norvegicus OX=10116 GN=C1qb PE=1 SV=2	1	3	26.6	25	↓	-0.74	→	-0.08
P01026	C3	Complement C3 OS=Rattus norvegicus OX=10116 GN=C3 PE=1 SV=3	66	633	186.3	9254	↓	-0.75	→	-0.24
P97594	Mcpt8	Mast cell protease 8 OS=Rattus norvegicus OX=10116 GN=Mcpt8 PE=2 SV=1	1	6	27.5	90	↓	-0.76	→	-0.37
Q3SWS9	Jakmip1	Janus kinase and microtubule-interacting protein 1 OS=Rattus norvegicus OX=10116 GN=Jakmip1 PE=1 SV=1	1	2	73.1	0	↓	-0.78	↓	-0.95
Q9ERC5	Otof	Otoferlin OS=Rattus norvegicus OX=10116 GN=Otof PE=1 SV=2	1	1	226.2	28	↓	-0.82	→	-0.27
P15429	Eno3	Beta-enolase OS=Rattus norvegicus OX=10116 GN=Eno3 PE=1 SV=3	8	60	47	796	↓	-0.88	↓	-0.93
A0JPN3	Bpifb1	BPI fold-containing family B member 1 OS=Rattus norvegicus OX=10116 GN=Bpifb1 PE=2 SV=1	1	1	52.2	0	↓	-1.06	↓	-0.53
P00564	Ckm	Creatine kinase M-type OS=Rattus norvegicus OX=10116 GN=Ckm PE=1 SV=2	10	99	43	1347	↓	-1.07	↓	-1.07
P08932		T-kininogen 2 OS=Rattus norvegicus OX=10116 PE=1 SV=2	3	128	47.7	2470	↓	-1.39	→	-0.07
P70623	Fabp4	Fatty acid-binding protein, adipocyte OS=Rattus norvegicus OX=10116 GN=Fabp4 PE=1 SV=3	4	16	14.7	157	↓	-1.43	↓	-1.59
P01048	Map1	T-kininogen 1 OS=Rattus norvegicus OX=10116 GN=Map1 PE=1 SV=2	5	119	47.7	1816	↓	-1.68	↓	-0.45
P14141	Ca3	Carbonic anhydrase 3 OS=Rattus norvegicus OX=10116 GN=Ca3 PE=1 SV=3	10	68	29.4	1066	↓	-2.17	↓	-2.66

Table S2. The progression related DEPs in GAS- and PM1-exposed groups

Accession	Gnen Name	Description	#Unique Peptides	#PSMs	MW [kDa]	Mascot Score	GAS-6M/3M (log_2Ratio)	PM1-6M/3M (log_2Ratio)
P04466	Mylpf	Myosin regulatory light chain 2, skeletal muscle isoform OS=Rattus norvegicus OX=10116 GN=Mylpf PE=1 SV=2	3	15	19	41	↑ 3.06	→ 0.27
Q64578	Atp2a1	Sarcoplasmic/endoplasmic reticulum calcium ATPase 1 OS=Rattus norvegicus OX=10116 GN=Atp2a1 PE=1 SV=1	3	58	109.3	723	↑ 3.06	↑ 0.78
P14141	Ca3	Carbonic anhydrase 3 OS=Rattus norvegicus OX=10116 GN=Ca3 PE=1 SV=3	10	68	29.4	1066	↑ 2.89	→ 0.07
Q5RKH1	Prpf4b	Serine/threonine-protein kinase PRP4 homolog OS=Rattus norvegicus OX=10116 GN=Prpf4b PE=1 SV=1	1	1	116.9	25	↑ 2.85	↑ 0.65
P12847	Myh3	Myosin-3 OS=Rattus norvegicus OX=10116 GN=Myh3 PE=3 SV=1	6	19	223.7	79	↑ 2.38	→ 0.12
Q62640	Grid1	Glutamate receptor ionotropic, delta-1 OS=Rattus norvegicus OX=10116 GN=Grid1 PE=2 SV=1	1	1	112.1	18	↑ 2.29	→ 0.11
P00564	Ckm	Creatine kinase M-type OS=Rattus norvegicus OX=10116 GN=Ckm PE=1 SV=2	10	99	43	1347	↑ 2.18	↓ -0.79
P15429	Eno3	Beta-enolase OS=Rattus norvegicus OX=10116 GN=Eno3 PE=1 SV=3	8	60	47	796	↑ 2.07	↓ -0.52
P16409	Myl3	Myosin light chain 3 OS=Rattus norvegicus OX=10116 GN=Myl3 PE=1 SV=2	1	18	22.1	162	↑ 1.98	→ -0.02
B0BNK9	Cracr2b	EF-hand calcium-binding domain-containing protein 4A OS=Rattus norvegicus OX=10116 GN=Cracr2b PE=2 SV=1	1	1	44.5	0	↑ 1.96	↑ 1.42
Q63189	Prg2	Bone marrow proteoglycan OS=Rattus norvegicus OX=10116 GN=Prg2 PE=2 SV=1	1	2	25.1	68	↑ 1.89	↑ 1.52
P19633	Casq1	Calsequestrin-1 OS=Rattus norvegicus OX=10116 GN=Casq1 PE=1 SV=2	1	3	46.4	48	↑ 1.84	→ 0.18
P00763	Prss2	Anionic trypsin-2 OS=Rattus norvegicus OX=10116 GN=Prss2 PE=1 SV=2	1	45	26.2	646	↑ 1.82	↑ 2.11
Q0H8B9	Clec2d11	C-type lectin domain family 2 member D11 OS=Rattus norvegicus OX=10116 GN=Clec2d11 PE=1 SV=1	4	29	23.5	528	↑ 1.77	→ 0.3
P47853	Bgn	Biglycan OS=Rattus norvegicus OX=10116 GN=Bgn PE=1 SV=1	5	31	41.7	301	↑ 1.76	↑ 1.86
Q63518	Mybpc1	Myosin-binding protein C, slow-type (Fragment) OS=Rattus norvegicus OX=10116 GN=Mybpc1 PE=1 SV=1	2	3	68.7	33	↑ 1.72	→ -0.34
Q07652	Cacna1e	Voltage-dependent R-type calcium channel subunit alpha-1E OS=Rattus norvegicus OX=10116 GN=Cacna1e PE=1 SV=1	1	3	252	0	↑ 1.66	↑ 2.19
Q32PX2	Aimp2	Aminoacyl tRNA synthase complex-interacting multifunctional protein 2 OS=Rattus norvegicus OX=10116 GN=Aimp2 PE=2 SV=1	3	13	35.4	115	↑ 1.49	→ 0.01
Q4G074	Kbp	KIF1-binding protein OS=Rattus norvegicus OX=10116 GN=Kbp PE=2 SV=1	1	4	71.3	64	↑ 1.42	→ 0
Q9QZ76	Mb	Myoglobin OS=Rattus norvegicus OX=10116 GN=Mb PE=1 SV=3	3	15	17.1	287	↑ 1.39	↓ -0.38
Q920P6	Ada	Adenosine deaminase OS=Rattus norvegicus OX=10116 GN=Ada PE=1 SV=3	11	43	39.9	344	↑ 1.38	→ 0
P70615	Lmnb1	Lamin-B1 OS=Rattus norvegicus OX=10116 GN=Lmnb1 PE=1 SV=3	14	62	66.6	754	↑ 1.34	↑ 1.18
O08984	Lbr	Delta(14)-sterol reductase OS=Rattus norvegicus OX=10116 GN=Lbr PE=1 SV=1	2	4	70.7	72	↑ 1.3	↑ 1.18

P62804	Hist1h4b	Histone H4 OS=Rattus norvegicus OX=10116 GN=Hist1h4b PE=1 SV=2	5	33	11.4	632	↑	1.29	↑	1.49
Q569C0	Tmem100	Transmembrane protein 100 OS=Rattus norvegicus OX=10116 GN=Tmem100 PE=1 SV=1	1	6	14.3	150	↑	1.29	↑	1.1
Q4KLH9	Gpr4	G-protein coupled receptor 4 OS=Rattus norvegicus OX=10116 GN=Gpr4 PE=2 SV=1	1	1	41.2	0	↑	1.22	↑	1.71
P48679	Lmna	Prelamin-A/C OS=Rattus norvegicus OX=10116 GN=Lmna PE=1 SV=1	27	202	74.3	2911	↑	1.22	↑	0.71
P80254	Ddt	D-dopachrome decarboxylase OS=Rattus norvegicus OX=10116 GN=Ddt PE=1 SV=3	2	3	13.1	29	↑	1.16	↑	1.72
P41350	Cav1	Caveolin-1 OS=Rattus norvegicus OX=10116 GN=Cav1 PE=1 SV=3	9	121	20.5	1449	↑	1.15	↑	0.88
Q6LED0		Histone H3.1 OS=Rattus norvegicus OX=10116 PE=1 SV=3	1	26	15.4	145	↑	1.11	↑	1.26
P02262		Histone H2A type 1 OS=Rattus norvegicus OX=10116 PE=1 SV=2	2	25	14.1	347	↑	1.1	↑	1.19
P50115	S100a8	Protein S100-A8 OS=Rattus norvegicus OX=10116 GN=S100a8 PE=1 SV=3	4	41	10.2	334	↑	1.06	↑	0.63
P50116	S100a9	Protein S100-A9 OS=Rattus norvegicus OX=10116 GN=S100a9 PE=1 SV=3	6	59	13.1	319	↑	1.03	↑	0.74
P58775	Tpm2	Tropomyosin beta chain OS=Rattus norvegicus OX=10116 GN=Tpm2 PE=1 SV=1	3	52	32.8	718	↑	1.03	↑	0.41
Q99MA2	Xpnpep2	Xaa-Pro aminopeptidase 2 OS=Rattus norvegicus OX=10116 GN=Xpnpep2 PE=1 SV=1	16	94	76	1369	↑	0.99	↑	0.74
P48284	Ca4	Carbonic anhydrase 4 OS=Rattus norvegicus OX=10116 GN=Ca4 PE=1 SV=1	5	24	35.1	255	↑	0.98	↑	0.52
P31430	Dpep1	Dipeptidase 1 OS=Rattus norvegicus OX=10116 GN=Dpep1 PE=2 SV=2	10	97	45.5	1807	↑	0.97	↑	0.92
Q9WTQ2	Podxl	Podocalyxin OS=Rattus norvegicus OX=10116 GN=Podxl PE=1 SV=2	2	3	51.6	57	↑	0.96	↑	0.78
P62997	Tra2b	Transformer-2 protein homolog beta OS=Rattus norvegicus OX=10116 GN=Tra2b PE=1 SV=1	3	10	33.6	144	↑	0.95	↑	0.67
Q9EQP5	Prelp	Prolargin OS=Rattus norvegicus OX=10116 GN=Prelp PE=2 SV=1	14	89	43.2	1619	↑	0.94	↑	1.01
Q01129	Dcn	Decorin OS=Rattus norvegicus OX=10116 GN=Dcn PE=1 SV=1	7	20	39.8	158	↑	0.94	↑	1.14
Q8K3M6	Erc2	ERC protein 2 OS=Rattus norvegicus OX=10116 GN=Erc2 PE=1 SV=1	1	1	110.6	0	↑	0.94	↑	1.1
Q9WV78	Plvap	Plasmalemma vesicle-associated protein OS=Rattus norvegicus OX=10116 GN=Plvap PE=1 SV=1	8	45	50	780	↑	0.93	↑	0.91
P63155	Crnkl1	Crooked neck-like protein 1 OS=Rattus norvegicus OX=10116 GN=Crnkl1 PE=2 SV=1	1	2	83.4	0	↑	0.92	↑	1.18
Q4V885	Colec12	Collectin-12 OS=Rattus norvegicus OX=10116 GN=Colec12 PE=2 SV=1	5	8	81.5	27	↑	0.9	↑	0.82
P00762	Prss1	Anionic trypsin-1 OS=Rattus norvegicus OX=10116 GN=Prss1 PE=1 SV=1	1	15	25.9	140	↑	0.87	↑	1.05
Q811A2	Bst2	Bone marrow stromal antigen 2 OS=Rattus norvegicus OX=10116 GN=Bst2 PE=1 SV=1	2	14	19.7	182	↑	0.87	↑	0.88
B2RZ86	Ccdc89	Coiled-coil domain-containing protein 89 OS=Rattus norvegicus OX=10116 GN=Ccdc89 PE=2 SV=2	1	1	43.4	0	↑	0.86	↑	0.91
Q27W01	Rbm8a	RNA-binding protein 8A OS=Rattus norvegicus OX=10116 GN=Rbm8a PE=1 SV=1	4	10	19.9	103	↑	0.85	↑	0.85
P08932		T-kininogen 2 OS=Rattus norvegicus OX=10116 PE=1 SV=2	3	128	47.7	2470	↑	0.84	→	0.02
P09812	Pygm	Glycogen phosphorylase, muscle form OS=Rattus norvegicus OX=10116 GN=Pygm PE=1 SV=5	16	130	97.2	1341	↑	0.84	→	-0.29
P54290	Cacna2d1	Voltage-dependent calcium channel subunit alpha-2/delta-1 OS=Rattus norvegicus OX=10116 GN=Cacna2d1 PE=1 SV=1	5	19	123.7	98	↑	0.82	↑	0.51
Q00729	Hist1h2ba	Histone H2B type 1-A OS=Rattus norvegicus OX=10116 GN=Hist1h2ba PE=1 SV=2	1	40	14.2	476	↑	0.82	↑	0.77
Q5RKI3	Poll	DNA polymerase lambda OS=Rattus norvegicus OX=10116 GN=Poll PE=2 SV=1	1	5	62.4	33	↑	0.82	↑	1.57

P42893	Ece1	Endothelin-converting enzyme 1 OS=Rattus norvegicus OX=10116 GN=Ece1 PE=1 SV=2	7	16	86.1	139	↑	0.81	↑	0.63
Q6URK4	Hnrnpa3	Heterogeneous nuclear ribonucleoprotein A3 OS=Rattus norvegicus OX=10116 GN=Hnrnpa3 PE=1 SV=1	7	43	39.6	737	↑	0.8	↑	0.78
P97544	Plpp3	Phospholipid phosphatase 3 OS=Rattus norvegicus OX=10116 GN=Plpp3 PE=1 SV=1	3	9	35.3	140	↑	0.8	↑	0.75
Q6AYK6	Cacybp	Calcyclin-binding protein OS=Rattus norvegicus OX=10116 GN=Cacybp PE=1 SV=1	4	22	26.5	285	↑	0.79	↑	0.46
P29414	Gja3	Gap junction alpha-3 protein OS=Rattus norvegicus OX=10116 GN=Gja3 PE=2 SV=2	1	2	46	17	↑	0.79	↑	0.82
Q6AYJ1	Recql	ATP-dependent DNA helicase Q1 OS=Rattus norvegicus OX=10116 GN=Recql PE=1 SV=1	1	1	69.6	29	↑	0.77	↑	0.64
Q2EJA0	Yap1	Transcriptional coactivator YAP1 OS=Rattus norvegicus OX=10116 GN=Yap1 PE=1 SV=1	1	1	50.5	21	↑	0.76	↑	0.55
Q5PQX1	Tor1aip1	Torsin-1A-interacting protein 1 OS=Rattus norvegicus OX=10116 GN=Tor1aip1 PE=1 SV=1	3	8	65.6	137	↑	0.75	↑	0.65
P70587	Lrrc7	Leucine-rich repeat-containing protein 7 OS=Rattus norvegicus OX=10116 GN=Lrrc7 PE=1 SV=2	1	3	166.8	0	↑	0.75	↑	0.63
P14740	Dpp4	Dipeptidyl peptidase 4 OS=Rattus norvegicus OX=10116 GN=Dpp4 PE=1 SV=2	20	177	88	2696	↑	0.75	↑	0.47
P01048	Map1	T-kininogen 1 OS=Rattus norvegicus OX=10116 GN=Map1 PE=1 SV=2	5	119	47.7	1816	↑	0.75	→	0
Q63089	Slc22a1	Solute carrier family 22 member 1 OS=Rattus norvegicus OX=10116 GN=Slc22a1 PE=1 SV=1	1	1	61.5	0	↑	0.74	→	0.36
A7VJC2	Hnrnpa2b1	Heterogeneous nuclear ribonucleoproteins A2/B1 OS=Rattus norvegicus OX=10116 GN=Hnrnpa2b1 PE=1 SV=1	17	204	37.5	3544	↑	0.74	↑	0.66
Q9R1J8	P3h1	Prolyl 3-hydroxylase 1 OS=Rattus norvegicus OX=10116 GN=P3h1 PE=1 SV=1	1	2	82.3	16	↑	0.74	→	-0.08
P35467	S100a1	Protein S100-A1 OS=Rattus norvegicus OX=10116 GN=S100a1 PE=1 SV=3	1	3	10.6	48	↑	0.73	↑	1.07
Q9ERA7	Msln	Mesothelin OS=Rattus norvegicus OX=10116 GN=Msln PE=2 SV=2	1	2	68.8	34	↑	0.73	↑	0.58
P97829	Cd47	Leukocyte surface antigen CD47 OS=Rattus norvegicus OX=10116 GN=Cd47 PE=1 SV=1	2	8	33	164	↑	0.72	↑	0.59
Q00715		Histone H2B type 1 OS=Rattus norvegicus OX=10116 PE=1 SV=2	2	44	14	683	↑	0.72	↑	0.48
O08658	Nup88	Nuclear pore complex protein Nup88 OS=Rattus norvegicus OX=10116 GN=Nup88 PE=1 SV=1	2	3	83.5	51	↑	0.72	↑	0.64
Q9Z2J4	Nexn	Nexilin OS=Rattus norvegicus OX=10116 GN=Nexn PE=1 SV=1	2	30	78.3	179	↑	0.71	↑	0.48
P15083	Pigr	Polymeric immunoglobulin receptor OS=Rattus norvegicus OX=10116 GN=Pigr PE=1 SV=1	8	27	84.7	319	↑	0.71	↑	0.53
Q6AY80	Nqo2	Ribosyldihydronicotinamide dehydrogenase [quinone] OS=Rattus norvegicus OX=10116 GN=Nqo2 PE=1 SV=3	5	17	26.3	244	↑	0.71	→	0.06
Q6AY31		Uncharacterized protein C4orf36 homolog OS=Rattus norvegicus OX=10116 PE=4 SV=1	1	3	13.3	36	↑	0.69	→	0.27
P61203	Cops2	COP9 signalosome complex subunit 2 OS=Rattus norvegicus OX=10116 GN=Cops2 PE=1 SV=1	9	22	51.6	229	↑	0.69	→	0.15
Q5U2W4	Cenpn	Centromere protein N OS=Rattus norvegicus OX=10116 GN=Cenpn PE=2 SV=1	1	2	39.4	0	↑	0.68	↑	1.16
P97687	Entpd1	Ectonucleoside triphosphate diphosphohydrolase 1 OS=Rattus norvegicus OX=10116 GN=Entpd1 PE=1 SV=1	5	23	57.4	209	↑	0.68	↑	0.66
Q6IG03	Krt73	Keratin, type II cytoskeletal 73 OS=Rattus norvegicus OX=10116 GN=Krt73 PE=1 SV=1	1	12	60.3	98	↑	0.68	→	0.3
P51886	Lum	Lumican OS=Rattus norvegicus OX=10116 GN=Lum PE=1 SV=1	7	46	38.3	611	↑	0.66	↑	0.79

Q00910	Slco2a1	Solute carrier organic anion transporter family member 2A1 OS=Rattus norvegicus OX=10116 GN=Slco2a1 PE=1 SV=3	8	31	70.3	456	↑	0.66	↑	0.62
Q7TP54	Ripor2	Rho family-interacting cell polarization regulator 2 OS=Rattus norvegicus OX=10116 GN=Ripor2 PE=1 SV=1	4	9	144.6	239	↑	0.65	↑	0.72
O35353	Gnb4	Guanine nucleotide-binding protein subunit beta-4 OS=Rattus norvegicus OX=10116 GN=Gnb4 PE=2 SV=4	3	37	37.3	940	↑	0.65	↑	0.59
P16290	Pgam2	Phosphoglycerate mutase 2 OS=Rattus norvegicus OX=10116 GN=Pgam2 PE=1 SV=2	3	28	28.7	592	↑	0.65	↓	-0.89
Q2IBC5	Cav2	Caveolin-2 OS=Rattus norvegicus OX=10116 GN=Cav2 PE=1 SV=2	4	10	18.3	137	↑	0.64	↑	0.85
P19814	Ttgn1	Trans-Golgi network integral membrane protein TGN38 OS=Rattus norvegicus OX=10116 GN=Ttgn1 PE=1 SV=1	2	5	38.3	73	↑	0.64	↑	0.95
P09650	Mcpt1	Mast cell protease 1 OS=Rattus norvegicus OX=10116 GN=Mcpt1 PE=1 SV=3	3	8	28.6	62	↑	0.64	↑	0.78
Q63862	Myh11	Myosin-11 (Fragments) OS=Rattus norvegicus OX=10116 GN=Myh11 PE=1 SV=3	16	93	152.4	1123	↑	0.63	↑	1.01
P97608	Oplah	5-oxoprolinase OS=Rattus norvegicus OX=10116 GN=Oplah PE=1 SV=2	9	13	137.6	78	↑	0.63	↑	0.87
Q62862	Map2k5	Dual specificity mitogen-activated protein kinase kinase 5 OS=Rattus norvegicus OX=10116 GN=Map2k5 PE=1 SV=1	1	3	50.2	58	↑	0.62	↑	0.53
Q63691	Cd14	Monocyte differentiation antigen CD14 OS=Rattus norvegicus OX=10116 GN=Cd14 PE=2 SV=2	1	1	40	23	↑	0.61	→	0.26
P47820	Ace	Angiotensin-converting enzyme OS=Rattus norvegicus OX=10116 GN=Ace PE=1 SV=1	26	261	150.8	3668	↑	0.61	↑	0.42
G3V9R8	Hnrnpc	Heterogeneous nuclear ribonucleoprotein C OS=Rattus norvegicus OX=10116 GN=Hnrnpc PE=1 SV=2	13	64	32.8	824	↑	0.61	↑	0.61
Q9JLT0	Myh10	Myosin-10 OS=Rattus norvegicus OX=10116 GN=Myh10 PE=1 SV=1	7	58	228.8	613	↑	0.6	↑	0.43
Q5XXR3	Arhgef6	Rho guanine nucleotide exchange factor 6 OS=Rattus norvegicus OX=10116 GN=Arhgef6 PE=1 SV=1	3	10	87	44	↑	0.6	→	0.14
P47858	Pfkm	ATP-dependent 6-phosphofructokinase, muscle type OS=Rattus norvegicus OX=10116 GN=Pfkm PE=1 SV=3	5	22	85.5	280	↑	0.6	→	-0.07
Q63190	Emd	Emerin OS=Rattus norvegicus OX=10116 GN=Emd PE=1 SV=1	1	2	29.7	42	↑	0.59	↑	0.57
B1WC88		UPF0729 protein C18orf32 homolog OS=Rattus norvegicus OX=10116 PE=3 SV=1	1	2	8.2	62	↑	0.59	↑	0.79
Q9JJM7	Gp1bb	Platelet glycoprotein Ib beta chain OS=Rattus norvegicus OX=10116 GN=Gp1bb PE=1 SV=1	1	9	22.2	33	↑	0.59	→	0.28
Q8VI02	Ppp4r1	Serine/threonine-protein phosphatase 4 regulatory subunit 1 OS=Rattus norvegicus OX=10116 GN=Ppp4r1 PE=1 SV=1	1	1	105.5	0	↑	0.59	→	0.25
Q6AYK1	Rnps1	RNA-binding protein with serine-rich domain 1 OS=Rattus norvegicus OX=10116 GN=Rnps1 PE=2 SV=1	1	2	34.2	0	↑	0.59	↑	0.6
D4AE41	Rbmxl1	RNA binding motif protein, X-linked-like-1 OS=Rattus norvegicus OX=10116 GN=Rbmxl1 PE=3 SV=1	10	22	42.2	172	↑	0.58	↑	0.48
Q9Z118	Ptbp3	Polypyrimidine tract-binding protein 3 OS=Rattus norvegicus OX=10116 GN=Ptbp3 PE=2 SV=1	2	21	56.7	366	↑	0.58	↑	0.75

P01946	Hba1	Hemoglobin subunit alpha-1/2 OS=Rattus norvegicus OX=10116 GN=Hba1 PE=1 SV=3	13	1815	15.3	25631	↑	0.57	↑	0.89
P04897	Gnai2	Guanine nucleotide-binding protein G(i) subunit alpha-2 OS=Rattus norvegicus OX=10116 GN=Gnai2 PE=1 SV=3	2	42	40.5	711	↑	0.57	↑	0.56
Q66H58	Ints14	Integrator complex subunit 14 OS=Rattus norvegicus OX=10116 GN=Ints14 PE=2 SV=1	1	1	57.1	22	↑	0.57	⇨	0.21
Q5EB81	Cyb5r1	NADH-cytochrome b5 reductase 1 OS=Rattus norvegicus OX=10116 GN=Cyb5r1 PE=2 SV=1	3	4	34.2	17	↑	0.57	⇨	0.05
P19132	Fth1	Ferritin heavy chain OS=Rattus norvegicus OX=10116 GN=Fth1 PE=1 SV=3	1	2	21.1	46	↑	0.56	↑	0.48
Q91XU1	Qki	Protein quaking OS=Rattus norvegicus OX=10116 GN=Qki PE=1 SV=2	5	18	37.6	177	↑	0.56	↑	0.42
Q5BJK8	Golim4	Golgi integral membrane protein 4 OS=Rattus norvegicus OX=10116 GN=Golim4 PE=1 SV=2	1	1	76.6	0	↑	0.55	↑	0.71
Q5U204	Lamtor3	Ragulator complex protein LAMTOR3 OS=Rattus norvegicus OX=10116 GN=Lamtor3 PE=2 SV=1	1	6	13.6	93	↑	0.55	↑	0.39
Q62745	Cd81	CD81 antigen OS=Rattus norvegicus OX=10116 GN=Cd81 PE=1 SV=1	1	4	25.9	49	↑	0.54	↑	0.61
O55164	Mpdz	Multiple PDZ domain protein OS=Rattus norvegicus OX=10116 GN=Mpdz PE=1 SV=1	1	1	218.5	25	↑	0.54	↑	0.43
P02696	Rbp1	Retinol-binding protein 1 OS=Rattus norvegicus OX=10116 GN=Rbp1 PE=1 SV=2	7	25	15.8	409	↑	0.53	↑	0.44
F1LNJ2	Snrnp200	U5 small nuclear ribonucleoprotein 200 kDa helicase OS=Rattus norvegicus OX=10116 GN=Snrnp200 PE=1 SV=1	19	46	244.7	336	↑	0.53	⇨	0.36
P40241	Cd9	CD9 antigen OS=Rattus norvegicus OX=10116 GN=Cd9 PE=1 SV=2	3	44	25.2	418	↑	0.52	↑	0.49
Q63493	Cd1d	Antigen-presenting glycoprotein CD1d OS=Rattus norvegicus OX=10116 GN=Cd1d PE=2 SV=2	2	4	38.5	61	↑	0.52	↑	0.49
Q6MGD0	Cuta	Protein CutA OS=Rattus norvegicus OX=10116 GN=Cuta PE=1 SV=2	1	6	18.6	37	↑	0.52	⇨	0.12
Q6MGA9	Brd2	Bromodomain-containing protein 2 OS=Rattus norvegicus OX=10116 GN=Brd2 PE=1 SV=1	1	1	88	48	↑	0.52	⇨	-0.05
Q27W02	Magoh	Protein mago nashi homolog OS=Rattus norvegicus OX=10116 GN=Magoh PE=2 SV=1	3	11	17.2	254	↑	0.52	↑	0.46
Q4V8K5	Brox	BRO1 domain-containing protein BROX OS=Rattus norvegicus OX=10116 GN=Brox PE=2 SV=1	3	6	46.2	123	↑	0.52	↑	0.54
P11883	Aldh3a1	Aldehyde dehydrogenase, dimeric NADP-preferring OS=Rattus norvegicus OX=10116 GN=Aldh3a1 PE=1 SV=3	5	21	50.3	251	↑	0.52	⇨	-0.11
E9PTG8	Stk10	Serine/threonine-protein kinase 10 OS=Rattus norvegicus OX=10116 GN=Stk10 PE=1 SV=1	2	3	111.8	30	↑	0.51	↑	0.63
Q9ES71	Gnpat	Dihydroxyacetone phosphate acyltransferase OS=Rattus norvegicus OX=10116 GN=Gnpat PE=1 SV=1	3	7	77	88	↑	0.51	⇨	-0.22
Q63072	Bst1	ADP-ribosyl cyclase/cyclic ADP-ribose hydrolase 2 OS=Rattus norvegicus OX=10116 GN=Bst1 PE=2 SV=1	2	3	35.1	37	↑	0.5	↑	0.79
P50282	Mmp9	Matrix metalloproteinase-9 OS=Rattus norvegicus OX=10116 GN=Mmp9 PE=2 SV=1	1	4	78.6	86	↑	0.5	⇨	0.26
Q5RJY4	Dhrs7b	Dehydrogenase/reductase SDR family member 7B OS=Rattus norvegicus OX=10116 GN=Dhrs7b PE=1 SV=1	2	3	35.3	30	↑	0.5	↑	2.34
P85007	Ier3ip1	Immediate early response 3-interacting protein 1 OS=Rattus norvegicus OX=10116 GN=Ier3ip1 PE=3 SV=1	1	1	9	39	↑	0.5	⇨	0.24

O54698	Slc29a1	Equilibrative nucleoside transporter 1 OS=Rattus norvegicus OX=10116 GN=Slc29a1 PE=1 SV=3	2	33	50	554	↑	0.49	↑	0.55
P49134	Itgb1	Integrin beta-1 OS=Rattus norvegicus OX=10116 GN=Itgb1 PE=2 SV=1	11	109	88.4	2151	↑	0.49	↑	0.47
Q5M819	Psph	Phosphoserine phosphatase OS=Rattus norvegicus OX=10116 GN=Psph PE=2 SV=1	1	1	25	29	↑	0.49	→	0.33
Q8CHN6	Sgpl1	Sphingosine-1-phosphate lyase 1 OS=Rattus norvegicus OX=10116 GN=Sgpl1 PE=2 SV=1	2	9	63.7	125	↑	0.48	→	0.28
P18596	Atp2a3	Sarcoplasmic/endoplasmic reticulum calcium ATPase 3 OS=Rattus norvegicus OX=10116 GN=Atp2a3 PE=1 SV=2	8	41	116.2	324	↑	0.48	↑	0.57
Q9Z0U5	Aox1	Aldehyde oxidase 1 OS=Rattus norvegicus OX=10116 GN=Aox1 PE=1 SV=1	7	17	146.8	149	↑	0.48	→	0.05
O54772	Smarcd2	SWI/SNF-related matrix-associated actin-dependent regulator of chromatin subfamily D member 2 OS=Rattus norvegicus OX=10116 GN=Smarcd2 PE=1 SV=3	1	1	59.1	0	↑	0.48	→	0.18
O88984	Nxf1	Nuclear RNA export factor 1 OS=Rattus norvegicus OX=10116 GN=Nxf1 PE=2 SV=1	1	1	70.3	0	↑	0.47	↑	0.45
P29975	Aqp1	Aquaporin-1 OS=Rattus norvegicus OX=10116 GN=Aqp1 PE=1 SV=4	3	19	28.8	220	↑	0.47	↑	0.44
Q3T1I3	Ushbp1	Usher syndrome type-1C protein-binding protein 1 OS=Rattus norvegicus OX=10116 GN=Ushbp1 PE=2 SV=1	1	2	74.6	29	↑	0.47	→	0.18
O88453	Safb	Scaffold attachment factor B1 OS=Rattus norvegicus OX=10116 GN=Safb PE=1 SV=2	2	3	104.5	59	↑	0.47	→	0.1
P85125	Cavin1	Caveolae-associated protein 1 OS=Rattus norvegicus OX=10116 GN=Cavin1 PE=1 SV=1	16	178	43.9	3147	↑	0.47	→	0.27
P04256	Hnrnpa1	Heterogeneous nuclear ribonucleoprotein A1 OS=Rattus norvegicus OX=10116 GN=Hnrnpa1 PE=1 SV=3	3	53	34.2	659	↑	0.47	↑	0.42
Q6TRW4	Pds5b	Sister chromatid cohesion protein PDS5 homolog B OS=Rattus norvegicus OX=10116 GN=Pds5b PE=1 SV=2	2	8	164.4	90	↑	0.47	→	0.07
Q80X08	Washc2	WASH complex subunit 2 OS=Rattus norvegicus OX=10116 GN=Washc2 PE=1 SV=1	1	5	145.1	0	↑	0.47	→	0.01
Q63690	Bax	Apoptosis regulator BAX OS=Rattus norvegicus OX=10116 GN=Bax PE=1 SV=2	1	2	21.3	34	↑	0.46	→	0.29
P07861	Mme	Neprilysin OS=Rattus norvegicus OX=10116 GN=Mme PE=1 SV=2	8	27	85.7	106	↑	0.46	↑	0.54
Q9JKD6	Cldn5	Claudin-5 OS=Rattus norvegicus OX=10116 GN=Cldn5 PE=1 SV=2	2	12	23.1	128	↑	0.46	↑	0.84
Q6P791	Lamtor1	Ragulator complex protein LAMTOR1 OS=Rattus norvegicus OX=10116 GN=Lamtor1 PE=1 SV=1	1	3	17.7	66	↑	0.46	↑	0.46
P70478	Apc	Adenomatous polyposis coli protein OS=Rattus norvegicus OX=10116 GN=Apc PE=1 SV=1	1	1	310.3	0	↑	0.46	→	0.31
Q9JHJ1	Ramp2	Receptor activity-modifying protein 2 OS=Rattus norvegicus OX=10116 GN=Ramp2 PE=2 SV=1	1	7	20.3	72	↑	0.46	↑	0.73
P62738	Acta2	Actin, aortic smooth muscle OS=Rattus norvegicus OX=10116 GN=Acta2 PE=2 SV=1	7	925	42	16084	↑	0.46	→	0.27
Q6IMY8	Hnrnpu	Heterogeneous nuclear ribonucleoprotein U OS=Rattus norvegicus OX=10116 GN=Hnrnpu PE=1 SV=1	16	155	87.7	2179	↑	0.46	↑	0.47
P18589	Mx2	Interferon-induced GTP-binding protein Mx2 OS=Rattus norvegicus OX=10116 GN=Mx2 PE=2 SV=1	1	1	75	25	↑	0.46	→	0.22
Q920L7	Elovl5	Elongation of very long chain fatty acids protein 5 OS=Rattus norvegicus OX=10116 GN=Elovl5 PE=1 SV=1	1	4	35.2	59	↑	0.46	→	0.37

P61459	Pcbd1	Pterin-4-alpha-carbinolamine dehydratase OS=Rattus norvegicus OX=10116 GN=Pcbd1 PE=1 SV=2	1	2	12	37	↑	0.46	↑	0.38
P21396	Maoa	Amine oxidase [flavin-containing] A OS=Rattus norvegicus OX=10116 GN=Maoa PE=1 SV=1	4	23	59.5	185	↑	0.45	→	0.31
Q9Z2Q4	Mtr	Methionine synthase OS=Rattus norvegicus OX=10116 GN=Mtr PE=1 SV=1	1	3	139.1	0	↑	0.45	→	0.37
Q5U301	Akap2	A-kinase anchor protein 2 OS=Rattus norvegicus OX=10116 GN=Akap2 PE=1 SV=1	15	65	95.9	963	↑	0.45	↑	0.46
Q62835	Rabep2	Rab GTPase-binding effector protein 2 OS=Rattus norvegicus OX=10116 GN=Rabep2 PE=1 SV=1	4	7	61.9	51	↑	0.45	→	0.35
Q7TNY6	Acbd3	Golgi resident protein GCP60 OS=Rattus norvegicus OX=10116 GN=Acbd3 PE=1 SV=3	4	8	60.4	70	↑	0.45	↑	0.62
F1LQ48	Hnrnpl	Heterogeneous nuclear ribonucleoprotein L OS=Rattus norvegicus OX=10116 GN=Hnrnpl PE=1 SV=2	13	85	67.9	1283	↑	0.45	→	0.32
Q499V6	Zcrb1	Zinc finger CCHC-type and RNA-binding motif-containing protein 1 OS=Rattus norvegicus OX=10116 GN=Zcrb1 PE=2 SV=1	1	1	24.5	0	↑	0.45	→	0
B5DF91	Elavl1	ELAV-like protein 1 OS=Rattus norvegicus OX=10116 GN=Elavl1 PE=1 SV=1	7	31	36.1	501	↑	0.45	↑	0.61
Q56A27	Ncbp1	Nuclear cap-binding protein subunit 1 OS=Rattus norvegicus OX=10116 GN=Ncbp1 PE=1 SV=1	4	5	91.9	59	↑	0.45	↑	0.56
P11517		Hemoglobin subunit beta-2 OS=Rattus norvegicus OX=10116 PE=1 SV=2	2	2003	16	29023	↑	0.44	↑	0.74
Q9Z2S9	Flot2	Flotillin-2 OS=Rattus norvegicus OX=10116 GN=Flot2 PE=1 SV=1	9	26	47	372	↑	0.44	→	0.37
P47864	Aqp5	Aquaporin-5 OS=Rattus norvegicus OX=10116 GN=Aqp5 PE=2 SV=1	3	12	28.4	252	↑	0.44	↓	-0.45
Q8R508	Fat3	Protocadherin Fat 3 OS=Rattus norvegicus OX=10116 GN=Fat3 PE=1 SV=1	1	1	501.8	0	↑	0.44	→	0.37
A2RRT9	Cyp4v2	Cytochrome P450 4V2 OS=Rattus norvegicus OX=10116 GN=Cyp4v2 PE=2 SV=1	1	3	60.5	0	↑	0.43	→	-0.03
Q62826	Hnrnpm	Heterogeneous nuclear ribonucleoprotein M OS=Rattus norvegicus OX=10116 GN=Hnrnpm PE=1 SV=4	19	83	73.7	1317	↑	0.43	↑	0.43
Q68FY1	Nup35	Nucleoporin NUP35 OS=Rattus norvegicus OX=10116 GN=Nup35 PE=1 SV=1	2	2	34.8	0	↑	0.43	↑	0.7
O35795	Entpd2	Ectonucleoside triphosphate diphosphohydrolase 2 OS=Rattus norvegicus OX=10116 GN=Entpd2 PE=1 SV=1	1	1	54.4	0	↑	0.43	→	0.35
P26684	Ednra	Endothelin-1 receptor OS=Rattus norvegicus OX=10116 GN=Ednra PE=2 SV=2	1	2	48.2	57	↑	0.42	→	0.37
P70560	Col12a1	Collagen alpha-1(XII) chain (Fragment) OS=Rattus norvegicus OX=10116 GN=Col12a1 PE=2 SV=1	1	2	32	41	↑	0.42	↑	0.55
Q9QYW5	Emp3	Epithelial membrane protein 3 OS=Rattus norvegicus OX=10116 GN=Emp3 PE=2 SV=1	1	2	18.2	0	↑	0.42	↑	0.44
Q6AY87	Thoc6	THO complex subunit 6 homolog OS=Rattus norvegicus OX=10116 GN=Thoc6 PE=2 SV=1	2	4	37.4	40	↑	0.42	↑	0.63
P52796	Efnb1	Ephrin-B1 OS=Rattus norvegicus OX=10116 GN=Efnb1 PE=1 SV=1	3	4	37.9	26	↑	0.42	↑	0.46
Q4V8F9	Hsdl2	Hydroxysteroid dehydrogenase-like protein 2 OS=Rattus norvegicus OX=10116 GN=Hsdl2 PE=2 SV=1	5	13	58.3	205	↑	0.42	↑	0.65
P02764	Orm1	Alpha-1-acid glycoprotein OS=Rattus norvegicus OX=10116 GN=Orm1 PE=2 SV=1	4	13	23.6	86	↑	0.41	→	0.32

Q6UPR8	Ermp1	Endoplasmic reticulum metallopeptidase 1 OS=Rattus norvegicus OX=10116 GN=Ermp1 PE=1 SV=1	4	9	99.8	131	↑	0.41	↑	0.44
P36370	Tap1	Antigen peptide transporter 1 OS=Rattus norvegicus OX=10116 GN=Tap1 PE=1 SV=2	6	9	79.1	56	↑	0.41	↑	0.61
Q6P742	Plp2	Proteolipid protein 2 OS=Rattus norvegicus OX=10116 GN=Plp2 PE=2 SV=1	1	26	16.5	822	↑	0.41	→	0.11
Q66HF9	Lrrfip1	Leucine-rich repeat flightless-interacting protein 1 OS=Rattus norvegicus OX=10116 GN=Lrrfip1 PE=1 SV=1	5	11	80	65	↑	0.41	↑	0.82
B2RYW9	Fahd2	Fumarylacetoacetate hydrolase domain-containing protein 2 OS=Rattus norvegicus OX=10116 GN=Fahd2 PE=1 SV=1	5	11	34.6	154	↑	0.41	→	0.14
P24587	Akap5	A-kinase anchor protein 5 OS=Rattus norvegicus OX=10116 GN=Akap5 PE=1 SV=2	11	25	75.9	306	↑	0.4	↑	0.56
Q5BJQ2	Mindy1	Ubiquitin carboxyl-terminal hydrolase MINDY-1 OS=Rattus norvegicus OX=10116 GN=Mindy1 PE=2 SV=1	1	1	52.7	21	↑	0.4	↑	0.52
P21588	Nt5e	5'-nucleotidase OS=Rattus norvegicus OX=10116 GN=Nt5e PE=1 SV=1	8	16	63.9	140	↑	0.4	↑	0.63
Q3B8Q2	Eif4a3	Eukaryotic initiation factor 4A-III OS=Rattus norvegicus OX=10116 GN=Eif4a3 PE=1 SV=1	4	22	46.8	239	↑	0.4	→	0.3
P62944	Ap2b1	AP-2 complex subunit beta OS=Rattus norvegicus OX=10116 GN=Ap2b1 PE=1 SV=1	13	90	104.5	1532	↑	0.4	↑	0.5
Q7TPJ0	Ssr1	Translocon-associated protein subunit alpha OS=Rattus norvegicus OX=10116 GN=Ssr1 PE=1 SV=1	1	5	35.6	92	↑	0.4	↑	0.48
P80299	Ephx2	Bifunctional epoxide hydrolase 2 OS=Rattus norvegicus OX=10116 GN=Ephx2 PE=1 SV=1	2	5	62.3	69	↑	0.4	→	-0.05
Q5M840	G0s2	G0/G1 switch protein 2 OS=Rattus norvegicus OX=10116 GN=G0s2 PE=3 SV=1	1	2	11.2	0	↑	0.4	→	0.26
D3ZHR2	Abcd1	ATP-binding cassette sub-family D member 1 OS=Rattus norvegicus OX=10116 GN=Abcd1 PE=1 SV=1	1	1	81.9	0	↑	0.4	→	0.18
Q64716	Insrr	Insulin receptor-related protein OS=Rattus norvegicus OX=10116 GN=Insrr PE=1 SV=3	1	2	144.8	0	↑	0.39	→	-0.23
P11762	Lgals1	Galectin-1 OS=Rattus norvegicus OX=10116 GN=Lgals1 PE=1 SV=2	4	30	14.8	300	↑	0.39	→	0.36
P60570	Panx1	Pannexin-1 OS=Rattus norvegicus OX=10116 GN=Panx1 PE=1 SV=1	1	2	48	0	↑	0.39	→	0.17
O08557	Ddah1	N(G),N(G)-dimethylarginine dimethylaminohydrolase 1 OS=Rattus norvegicus OX=10116 GN=Ddah1 PE=1 SV=3	8	58	31.4	1091	↑	0.39	↑	0.48
Q9JIL3	Ilf3	Interleukin enhancer-binding factor 3 OS=Rattus norvegicus OX=10116 GN=Ilf3 PE=1 SV=2	8	22	95.9	244	↑	0.39	↑	0.42
Q66H98	Cavin2	Caveolae-associated protein 2 OS=Rattus norvegicus OX=10116 GN=Cavin2 PE=1 SV=3	15	147	46.4	2134	↑	0.39	→	0.27
P23562	Slc4a1	Band 3 anion transport protein OS=Rattus norvegicus OX=10116 GN=Slc4a1 PE=1 SV=3	19	129	103.1	2058	↑	0.39	↑	0.7
P25304	Agrn	Agrin OS=Rattus norvegicus OX=10116 GN=Agrn PE=1 SV=2	4	7	208.5	37	↑	0.39	↑	0.54
P08082	Cltb	Clathrin light chain B OS=Rattus norvegicus OX=10116 GN=Cltb PE=1 SV=1	7	24	25.1	260	↑	0.39	↑	0.4
P18437	Hmgn2	Non-histone chromosomal protein HMG-17 OS=Rattus norvegicus OX=10116 GN=Hmgn2 PE=1 SV=2	2	15	9.4	85	↑	0.39	→	0.34
Q5QD51	Akap12	A-kinase anchor protein 12 OS=Rattus norvegicus OX=10116 GN=Akap12 PE=1 SV=1	13	36	181	438	↑	0.38	↑	0.95
P30083	Vipr1	Vasoactive intestinal polypeptide receptor 1 OS=Rattus norvegicus OX=10116 GN=Vipr1 PE=2 SV=1	1	2	52	29	↑	0.38	↑	0.73

Q9QYV0	Adam15	Disintegrin and metalloproteinase domain-containing protein 15 OS=Rattus norvegicus OX=10116 GN=Adam15 PE=2 SV=2	1	1	93.2	17	↑	0.38	↑	0.52
P23347	Slc4a2	Anion exchange protein 2 OS=Rattus norvegicus OX=10116 GN=Slc4a2 PE=1 SV=1	2	4	136.6	76	↑	0.38	↑	0.54
Q99P74	Rab27b	Ras-related protein Rab-27B OS=Rattus norvegicus OX=10116 GN=Rab27b PE=2 SV=3	3	10	24.6	123	↑	0.38	→	0.19
Q66H86	Olfml1	Olfactomedin-like protein 1 OS=Rattus norvegicus OX=10116 GN=Olfml1 PE=2 SV=1	4	16	45.6	163	↑	0.38	↑	0.45
Q5S6T3	Crppa	D-ribitol-5-phosphate cytidylyltransferase OS=Rattus norvegicus OX=10116 GN=Ispd PE=2 SV=1	3	3	49.2	42	↑	0.38	→	0.21
P59215	Gnao1	Guanine nucleotide-binding protein G(o) subunit alpha OS=Rattus norvegicus OX=10116 GN=Gnao1 PE=1 SV=2	4	25	40	518	↑	0.38	→	0.18
Q6AYC2	Irgm	Immunity-related GTPase family M protein OS=Rattus norvegicus OX=10116 GN=Irgm PE=2 SV=1	5	12	46.3	92	↑	0.38	↑	0.42
Q9WVT0	Adgrf5	Adhesion G protein-coupled receptor F5 OS=Rattus norvegicus OX=10116 GN=Adgrf5 PE=1 SV=1	10	18	149.4	194	↑	0.38	↑	0.69
Q8CFN2	Cdc42	Cell division control protein 42 homolog OS=Rattus norvegicus OX=10116 GN=Cdc42 PE=1 SV=2	3	68	21.2	1041	↑	0.38	↑	0.48
Q5PPK9	Eipr1	EARP and GARP complex-interacting protein 1 OS=Rattus norvegicus OX=10116 GN=Eipr1 PE=1 SV=1	2	3	43.1	26	↑	0.38	→	0.14
P30009	Marcks	Myristoylated alanine-rich C-kinase substrate OS=Rattus norvegicus OX=10116 GN=Marcks PE=1 SV=2	6	40	29.8	677	→	0.37	↑	0.76
Q63425	Prx	Periaxin OS=Rattus norvegicus OX=10116 GN=Prx PE=1 SV=2	42	286	146.3	3181	→	0.37	↑	0.42
Q794F9	Slc3a2	4F2 cell-surface antigen heavy chain OS=Rattus norvegicus OX=10116 GN=Slc3a2 PE=1 SV=1	3	9	58	143	→	0.37	↑	0.58
Q920G2	Slc9a3r2	Na(+)/H(+) exchange regulatory cofactor NHE-RF2 OS=Rattus norvegicus OX=10116 GN=Slc9a3r2 PE=1 SV=1	10	42	37.3	870	→	0.36	↑	0.72
Q5RK30	Sbds	Ribosome maturation protein SBDS OS=Rattus norvegicus OX=10116 GN=Sbds PE=2 SV=1	9	34	28.7	589	→	0.36	↑	0.43
P50123	Enpep	Glutamyl aminopeptidase OS=Rattus norvegicus OX=10116 GN=Enpep PE=1 SV=2	18	71	107.9	743	→	0.36	↑	0.38
P17764	Acat1	Acetyl-CoA acetyltransferase, mitochondrial OS=Rattus norvegicus OX=10116 GN=Acat1 PE=1 SV=1	7	39	44.7	651	→	0.36	↑	0.39
Q3SWS9	Jakmip1	Janus kinase and microtubule-interacting protein 1 OS=Rattus norvegicus OX=10116 GN=Jakmip1 PE=1 SV=1	1	2	73.1	0	→	0.36	↑	1.08
Q9JKL7	Srek1	Splicing regulatory glutamine/lysine-rich protein 1 OS=Rattus norvegicus OX=10116 GN=Srek1 PE=1 SV=1	1	1	56.8	35	→	0.35	↑	0.92
Q920Q0	Palm	Paralemmin-1 OS=Rattus norvegicus OX=10116 GN=Palm PE=1 SV=1	6	13	41.9	87	→	0.35	↑	0.56
P36860	Ralb	Ras-related protein Ral-B OS=Rattus norvegicus OX=10116 GN=Ralb PE=2 SV=1	1	13	23.3	183	→	0.35	↑	0.43
Q05175	Basp1	Brain acid soluble protein 1 OS=Rattus norvegicus OX=10116 GN=Basp1 PE=1 SV=2	3	9	21.8	60	→	0.35	↑	0.84
P62632	Eef1a2	Elongation factor 1-alpha 2 OS=Rattus norvegicus OX=10116 GN=Eef1a2 PE=1 SV=1	1	123	50.4	1882	→	0.35	↓	-0.67

P19944	Rplp1	60S acidic ribosomal protein P1 OS=Rattus norvegicus OX=10116 GN=Rplp1 PE=3 SV=1	1	5	11.5	136	⇨	0.34	⇧	0.53
Q07647	Slc2a3	Solute carrier family 2, facilitated glucose transporter member 3 OS=Rattus norvegicus OX=10116 GN=Slc2a3 PE=1 SV=1	1	2	53.5	37	⇨	0.34	⇧	0.41
P62329	Tmsb4x	Thymosin beta-4 OS=Rattus norvegicus OX=10116 GN=Tmsb4x PE=1 SV=2	3	35	5.1	287	⇨	0.34	⇧	0.58
P52631	Stat3	Signal transducer and activator of transcription 3 OS=Rattus norvegicus OX=10116 GN=Stat3 PE=1 SV=1	10	30	88	407	⇨	0.33	⇧	0.68
Q3SWU3	Hnrnpdl	Heterogeneous nuclear ribonucleoprotein D-like OS=Rattus norvegicus OX=10116 GN=Hnrnpdl PE=1 SV=1	3	7	35.3	114	⇨	0.33	⇧	0.47
Q08463	Fzd1	Frizzled-1 OS=Rattus norvegicus OX=10116 GN=Fzd1 PE=1 SV=1	1	1	71	26	⇨	0.32	⇧	0.39
Q5XI60	Reep6	Receptor expression-enhancing protein 6 OS=Rattus norvegicus OX=10116 GN=Reep6 PE=2 SV=1	1	7	23.3	16	⇨	0.32	⇩	-0.49
P63164	Snrpn	Small nuclear ribonucleoprotein-associated protein N OS=Rattus norvegicus OX=10116 GN=Snrpn PE=2 SV=1	3	9	24.6	73	⇨	0.32	⇧	0.5
P19332	Mapt	Microtubule-associated protein tau OS=Rattus norvegicus OX=10116 GN=Mapt PE=1 SV=3	8	30	78.5	207	⇨	0.31	⇧	0.5
Q9EQX9	Ube2n	Ubiquitin-conjugating enzyme E2 N OS=Rattus norvegicus OX=10116 GN=Ube2n PE=1 SV=1	6	30	17.1	537	⇨	0.31	⇧	0.41
Q5PQP1	Rbms1	RNA-binding motif, single-stranded-interacting protein 1 OS=Rattus norvegicus OX=10116 GN=Rbms1 PE=2 SV=1	1	2	44	0	⇨	0.31	⇧	0.45
P30099	Cyp11b2	Cytochrome P450 11B2, mitochondrial OS=Rattus norvegicus OX=10116 GN=Cyp11b2 PE=1 SV=1	1	1	58.2	0	⇨	0.31	⇧	0.55
Q02765	Ctss	Cathepsin S OS=Rattus norvegicus OX=10116 GN=Ctss PE=2 SV=1	1	6	36.8	57	⇨	0.31	⇧	0.7
B2RZ37	Reep5	Receptor expression-enhancing protein 5 OS=Rattus norvegicus OX=10116 GN=Reep5 PE=1 SV=1	4	30	21.4	675	⇨	0.3	⇧	0.43
P97690	Smc3	Structural maintenance of chromosomes protein 3 OS=Rattus norvegicus OX=10116 GN=Smc3 PE=1 SV=1	5	14	138.4	122	⇨	0.3	⇧	0.49
P0C219	Slmap	Sarcolemmal membrane-associated protein OS=Rattus norvegicus OX=10116 GN=Slmap PE=1 SV=1	5	10	98.2	78	⇨	0.29	⇧	0.52
Q68FR2	Bin2	Bridging integrator 2 OS=Rattus norvegicus OX=10116 GN=Bin2 PE=1 SV=1	3	10	55.1	61	⇨	0.29	⇧	0.43
D3ZCL3	Snrpc	U1 small nuclear ribonucleoprotein C OS=Rattus norvegicus OX=10116 GN=Snrpc PE=3 SV=1	1	1	17.4	37	⇨	0.28	⇧	0.47
P02091	Hbb	Hemoglobin subunit beta-1 OS=Rattus norvegicus OX=10116 GN=Hbb PE=1 SV=3	7	3732	16	60607	⇨	0.28	⇧	0.63
P63025	Vamp3	Vesicle-associated membrane protein 3 OS=Rattus norvegicus OX=10116 GN=Vamp3 PE=1 SV=1	3	13	11.5	357	⇨	0.28	⇧	0.42
P62749	Hpcal1	Hippocalcin-like protein 1 OS=Rattus norvegicus OX=10116 GN=Hpcal1 PE=1 SV=2	3	39	22.3	336	⇨	0.28	⇧	0.43
P19139	Csnk2a1	Casein kinase II subunit alpha OS=Rattus norvegicus OX=10116 GN=Csnk2a1 PE=1 SV=2	3	10	45	146	⇨	0.28	⇧	0.45

Q9Z269	Vapb	Vesicle-associated membrane protein-associated protein B OS=Rattus norvegicus OX=10116 GN=Vapb PE=1 SV=3	3	11	26.9	199	→	0.28	↑	0.39
Q6AYD6	Pdlim2	PDZ and LIM domain protein 2 OS=Rattus norvegicus OX=10116 GN=Pdlim2 PE=1 SV=1	5	7	37.6	65	→	0.27	↑	0.47
D3ZAP3	Map10	Microtubule-associated protein 10 OS=Rattus norvegicus OX=10116 GN=Map10 PE=3 SV=1	1	2	96.2	26	→	0.27	↑	0.77
Q5M7V8	Thrap3	Thyroid hormone receptor-associated protein 3 OS=Rattus norvegicus OX=10116 GN=Thrap3 PE=1 SV=1	7	16	108.2	38	→	0.27	↑	0.43
P49793	Nup98	Nuclear pore complex protein Nup98-Nup96 OS=Rattus norvegicus OX=10116 GN=Nup98 PE=1 SV=2	3	11	197.2	76	→	0.27	↑	0.51
P05943	S100a10	Protein S100-A10 OS=Rattus norvegicus OX=10116 GN=S100a10 PE=1 SV=2	3	23	11.1	223	→	0.27	↑	0.52
Q9EPF2	Mcam	Cell surface glycoprotein MUC18 OS=Rattus norvegicus OX=10116 GN=Mcam PE=1 SV=2	9	55	71.3	743	→	0.27	↑	0.44
Q80WE1	Fmr1	Synaptic functional regulator FMR1 OS=Rattus norvegicus OX=10116 GN=Fmr1 PE=1 SV=2	3	7	66.7	78	→	0.27	↑	0.43
O70535	Lifr	Leukemia inhibitory factor receptor OS=Rattus norvegicus OX=10116 GN=Lifr PE=2 SV=1	4	6	122.3	104	→	0.26	↑	0.41
Q9EPH2	Marcksl1	MARCKS-related protein OS=Rattus norvegicus OX=10116 GN=Marcksl1 PE=2 SV=3	2	9	19.8	123	→	0.26	↑	0.73
Q99MM4	Trafd1	TRAF-type zinc finger domain-containing protein 1 OS=Rattus norvegicus OX=10116 GN=Trafd1 PE=1 SV=2	1	1	63.8	0	→	0.26	↑	0.42
P62775	Mtpn	Myotrophin OS=Rattus norvegicus OX=10116 GN=Mtpn PE=1 SV=2	3	7	12.9	78	→	0.26	↑	0.67
P61621	Sec61a1	Protein transport protein Sec61 subunit alpha isoform 1 OS=Rattus norvegicus OX=10116 GN=Sec61a1 PE=2 SV=2	3	23	52.2	299	→	0.25	↑	0.42
Q02759	Alox15	Arachidonate 15-lipoxygenase OS=Rattus norvegicus OX=10116 GN=Alox15 PE=1 SV=3	2	2	75.3	29	→	0.25	↑	1.4
Q9ER30	Klhl41	Kelch-like protein 41 OS=Rattus norvegicus OX=10116 GN=Klhl41 PE=1 SV=1	1	1	68.2	0	→	0.25	↓	-0.74
P47875	Csrp1	Cysteine and glycine-rich protein 1 OS=Rattus norvegicus OX=10116 GN=Csrp1 PE=1 SV=2	8	88	20.6	1802	→	0.24	↑	0.55
B4F777	Hmgn5	High mobility group nucleosome-binding domain-containing protein 5 OS=Rattus norvegicus OX=10116 GN=Hmgn5 PE=2 SV=1	1	3	48.6	115	→	0.24	↑	0.69
B4F795	Slc44a2	Choline transporter-like protein 2 OS=Rattus norvegicus OX=10116 GN=Slc44a2 PE=2 SV=1	5	10	79.8	95	→	0.24	↑	0.44
P52590	Nup107	Nuclear pore complex protein Nup107 OS=Rattus norvegicus OX=10116 GN=Nup107 PE=1 SV=1	1	4	107.1	0	→	0.24	↑	0.53
Q5QE78	Aox2	Aldehyde oxidase 2 OS=Rattus norvegicus OX=10116 GN=Aox2 PE=2 SV=1	1	5	147.8	43	→	0.23	↑	0.44
Q4KMA2	Rad23b	UV excision repair protein RAD23 homolog B OS=Rattus norvegicus OX=10116 GN=Rad23b PE=1 SV=1	7	54	43.5	675	→	0.23	↑	0.51
P51870	Cyp4f5	Cytochrome P450 4F5 OS=Rattus norvegicus OX=10116 GN=Cyp4f5 PE=2 SV=1	2	4	60.6	50	→	0.23	↑	0.54
P97943	Scarb1	Scavenger receptor class B member 1 OS=Rattus norvegicus OX=10116 GN=Scarb1 PE=1 SV=1	2	8	56.9	34	→	0.23	↑	0.4
Q5FVQ9	Tbce	Tubulin-specific chaperone E OS=Rattus norvegicus OX=10116 GN=Tbce PE=2 SV=1	2	4	59	51	→	0.22	↑	0.79
Q62751	Ireb2	Iron-responsive element-binding protein 2 OS=Rattus norvegicus OX=10116 GN=Ireb2 PE=1 SV=2	1	2	104.7	48	→	0.22	↑	0.63

Q66HR2	Mapre1	Microtubule-associated protein RP/EB family member 1 OS=Rattus norvegicus OX=10116 GN=Mapre1 PE=1 SV=3	5	40	30	631	→	0.22	↑	0.48
Q9QY16	Ddx25	ATP-dependent RNA helicase DDX25 OS=Rattus norvegicus OX=10116 GN=Ddx25 PE=1 SV=2	1	2	54.8	55	→	0.22	↑	0.44
O35397	Casp6	Caspase-6 OS=Rattus norvegicus OX=10116 GN=Casp6 PE=2 SV=2	3	7	31.5	52	→	0.22	↓	-0.42
P20070	Cyb5r3	NADH-cytochrome b5 reductase 3 OS=Rattus norvegicus OX=10116 GN=Cyb5r3 PE=1 SV=2	8	83	34.2	1269	→	0.21	↑	0.67
Q6TUD4	Yipf3	Protein YIPF3 OS=Rattus norvegicus OX=10116 GN=Yipf3 PE=2 SV=1	1	2	37.9	29	→	0.21	↑	0.38
B0BN85	Sugt1	Protein SGT1 homolog OS=Rattus norvegicus OX=10116 GN=Sugt1 PE=2 SV=1	5	11	38.1	136	→	0.21	↑	0.43
P27435	Tpsab1	Tryptase OS=Rattus norvegicus OX=10116 GN=Tpsab1 PE=1 SV=2	2	6	30.4	201	→	0.21	↑	0.41
Q5PPH0	Enoph1	Enolase-phosphatase E1 OS=Rattus norvegicus OX=10116 GN=Enoph1 PE=2 SV=1	2	8	28.9	40	→	0.21	↓	-0.4
Q62739	Rab3ip	Rab-3A-interacting protein OS=Rattus norvegicus OX=10116 GN=Rab3ip PE=1 SV=1	1	1	50.9	20	→	0.2	↑	0.6
P70475	Myt1l	Myelin transcription factor 1-like protein OS=Rattus norvegicus OX=10116 GN=Myt1l PE=1 SV=3	1	1	132.8	25	→	0.2	↑	0.7
Q6IRI9	Fmo2	Dimethylaniline monooxygenase [N-oxide-forming] 2 OS=Rattus norvegicus OX=10116 GN=Fmo2 PE=2 SV=3	4	23	60.9	204	→	0.2	↓	-1.93
Q3MIE4	Vat1	Synaptic vesicle membrane protein VAT-1 homolog OS=Rattus norvegicus OX=10116 GN=Vat1 PE=1 SV=1	11	110	43.1	2965	→	0.19	↑	0.43
Q7TP47	Syncrip	Heterogeneous nuclear ribonucleoprotein Q OS=Rattus norvegicus OX=10116 GN=Syncrip PE=2 SV=1	11	39	59.7	303	→	0.19	↑	0.44
P00884	Aldob	Fructose-bisphosphate aldolase B OS=Rattus norvegicus OX=10116 GN=Aldob PE=1 SV=2	1	8	39.6	59	→	0.19	↑	0.79
O70257	Stx7	Syntaxin-7 OS=Rattus norvegicus OX=10116 GN=Stx7 PE=1 SV=4	4	14	29.8	90	→	0.18	↑	0.48
Q9JJP9	Ubqln1	Ubiquilin-1 OS=Rattus norvegicus OX=10116 GN=Ubqln1 PE=1 SV=1	1	1	62	0	→	0.18	↓	-0.44
Q9Z1E1	Flot1	Flotillin-1 OS=Rattus norvegicus OX=10116 GN=Flot1 PE=2 SV=2	8	20	47.5	191	→	0.17	↑	0.54
Q9ESH6	Glrx	Glutaredoxin-1 OS=Rattus norvegicus OX=10116 GN=Glrx PE=3 SV=3	1	13	11.9	187	→	0.17	↑	0.45
Q9QY78	Ikbkb	Inhibitor of nuclear factor kappa-B kinase subunit beta OS=Rattus norvegicus OX=10116 GN=Ikbkb PE=1 SV=1	1	2	86.8	32	→	0.17	↑	0.43
O35964	Sh3gl1	Endophilin-A2 OS=Rattus norvegicus OX=10116 GN=Sh3gl1 PE=1 SV=1	7	26	41.5	291	→	0.17	↑	0.41
O88869	Rassf9	Ras association domain-containing protein 9 OS=Rattus norvegicus OX=10116 GN=Rassf9 PE=1 SV=1	1	1	49.5	0	→	0.17	↑	0.57
P84245	H3f3b	Histone H3.3 OS=Rattus norvegicus OX=10116 GN=H3f3b PE=1 SV=2	1	25	15.3	127	→	0.17	↑	0.38
Q9JHL4	Dbnl	Drebrin-like protein OS=Rattus norvegicus OX=10116 GN=Dbnl PE=1 SV=1	5	20	48.6	251	→	0.16	↑	0.62
Q5I0H4	Tmco1	Calcium load-activated calcium channel OS=Rattus norvegicus OX=10116 GN=Tmco1 PE=2 SV=1	1	2	21.2	42	→	0.16	↑	0.41
Q5HZY0	Ubxn4	UBX domain-containing protein 4 OS=Rattus norvegicus OX=10116 GN=Ubxn4 PE=1 SV=1	3	5	56.4	42	→	0.16	↑	0.52
Q63118	Calcrl	Calcitonin gene-related peptide type 1 receptor OS=Rattus norvegicus OX=10116 GN=Calcrl PE=2 SV=1	1	4	53.3	25	→	0.16	↑	0.53

P11730	Camk2g	Calcium/calmodulin-dependent protein kinase type II subunit gamma OS=Rattus norvegicus OX=10116 GN=Camk2g PE=1 SV=1	5	28	59	380	→	0.16	↑	0.4
Q9QXY4	Ogfr	Opioid growth factor receptor OS=Rattus norvegicus OX=10116 GN=Ogfr PE=1 SV=1	1	1	64.7	35	→	0.16	↑	0.68
P06907	Mpz	Myelin protein P0 OS=Rattus norvegicus OX=10116 GN=Mpz PE=1 SV=1	1	1	27.6	0	→	0.16	↑	1.16
P11654	Nup210	Nuclear pore membrane glycoprotein 210 OS=Rattus norvegicus OX=10116 GN=Nup210 PE=1 SV=1	1	1	204	0	→	0.15	↑	0.7
P15978	RT1-Aw2	Class I histocompatibility antigen, Non-RT1.A alpha-1 chain OS=Rattus norvegicus OX=10116 GN=RT1-Aw2 PE=1 SV=1	1	35	36.5	631	→	0.15	↑	0.57
Q6VEU1	Nob1	RNA-binding protein NOB1 OS=Rattus norvegicus OX=10116 GN=Nob1 PE=2 SV=1	1	1	46.4	16	→	0.15	↓	-0.41
Q5M7W5	Map4	Microtubule-associated protein 4 OS=Rattus norvegicus OX=10116 GN=Map4 PE=1 SV=1	28	148	110.2	1785	→	0.14	↑	0.69
Q5M9G3	Caprin1	Caprin-1 OS=Rattus norvegicus OX=10116 GN=Caprin1 PE=1 SV=2	2	3	78.1	0	→	0.14	↑	0.51
O35162	Hspa13	Heat shock 70 kDa protein 13 OS=Rattus norvegicus OX=10116 GN=Hspa13 PE=1 SV=2	2	3	51.8	83	→	0.14	↑	0.39
D3ZTX0	Tmed7	Transmembrane emp24 domain-containing protein 7 OS=Rattus norvegicus OX=10116 GN=Tmed7 PE=1 SV=1	2	4	25.5	86	→	0.14	↑	0.38
P52632	Stat5b	Signal transducer and activator of transcription 5B OS=Rattus norvegicus OX=10116 GN=Stat5b PE=1 SV=1	4	6	90.2	37	→	0.13	↑	0.4
Q80ZG5	Slu7	Pre-mRNA-splicing factor SLU7 OS=Rattus norvegicus OX=10116 GN=Slu7 PE=1 SV=2	1	1	68.3	0	→	0.13	↑	0.47
P51868	Casq2	Calsequestrin-2 OS=Rattus norvegicus OX=10116 GN=Casq2 PE=1 SV=2	3	4	47.8	21	→	0.13	↓	-0.53
Q9WTR8	Phlpp1	PH domain leucine-rich repeat protein phosphatase 1 OS=Rattus norvegicus OX=10116 GN=Phlpp1 PE=1 SV=1	1	1	183.2	0	→	0.12	↑	0.61
Q6AYI5	Shoc2	Leucine-rich repeat protein SHOC-2 OS=Rattus norvegicus OX=10116 GN=Shoc2 PE=2 SV=1	1	1	64.9	0	→	0.12	↑	1.87
Q91Y81	Septin2	Septin-2 OS=Rattus norvegicus OX=10116 GN=Sept2 PE=1 SV=1	12	74	41.6	1203	→	0.12	↑	0.47
P23711	Hmox2	Heme oxygenase 2 OS=Rattus norvegicus OX=10116 GN=Hmox2 PE=1 SV=1	3	4	35.7	89	→	0.12	↑	0.52
Q03555	Gphn	Gephyrin OS=Rattus norvegicus OX=10116 GN=Gphn PE=1 SV=3	1	1	83.2	25	→	0.11	↑	0.52
P08934	Kng1	Kininogen-1 OS=Rattus norvegicus OX=10116 GN=Kng1 PE=2 SV=1	6	71	70.9	1723	→	0.11	↓	-0.42
Q6IFU7	Krt42	Keratin, type I cytoskeletal 42 OS=Rattus norvegicus OX=10116 GN=Krt42 PE=3 SV=1	3	126	50.2	1447	→	0.11	↓	-0.64
Q641X9	Mrpl9	39S ribosomal protein L9, mitochondrial OS=Rattus norvegicus OX=10116 GN=Mrpl9 PE=2 SV=1	1	1	30.1	0	→	0.1	↑	0.39
Q6AYP5	Cadm1	Cell adhesion molecule 1 OS=Rattus norvegicus OX=10116 GN=Cadm1 PE=1 SV=1	1	4	51.8	61	→	0.09	↑	0.8
P27274	Cd59	CD59 glycoprotein OS=Rattus norvegicus OX=10116 GN=Cd59 PE=1 SV=2	2	3	13.8	24	→	0.09	↓	-0.38
Q5M823	Nudcd2	NudC domain-containing protein 2 OS=Rattus norvegicus OX=10116 GN=Nudcd2 PE=2 SV=1	1	1	17.7	0	→	0.08	↑	0.7
P69060	Cmas	N-acylneuraminate cytidylyltransferase OS=Rattus norvegicus OX=10116 GN=Cmas PE=2 SV=1	4	15	48.1	186	→	0.07	↓	-0.44
P54275	Msh2	DNA mismatch repair protein Msh2 OS=Rattus norvegicus OX=10116 GN=Msh2 PE=2 SV=1	1	7	104	0	→	0.07	↑	0.57
P20717	Padi2	Protein-arginine deiminase type-2 OS=Rattus norvegicus OX=10116 GN=Padi2 PE=1 SV=1	1	1	75.3	22	→	0.06	↑	0.54

P0C644	Ppip5k1	Inositol hexakisphosphate and diphosphoinositol-pentakisphosphate kinase 1 OS=Rattus norvegicus OX=10116 GN=Ppip5k1 PE=1 SV=1	1	2	159.5	50	→	0.06	↓	-0.39
P27321	Cast	Calpastatin OS=Rattus norvegicus OX=10116 GN=Cast PE=1 SV=3	9	43	77.3	316	→	0.05	↑	0.44
P53812	Pitpnb	Phosphatidylinositol transfer protein beta isoform OS=Rattus norvegicus OX=10116 GN=Pitpnb PE=1 SV=2	3	14	31.4	146	→	0.05	↑	0.46
Q06606	Mcpt10	Granzyme-like protein 2 OS=Rattus norvegicus OX=10116 GN=Mcpt10 PE=2 SV=1	1	5	27.4	54	→	0.05	↓	-0.63
Q00972	Bckdk	[3-methyl-2-oxobutanoate dehydrogenase [lipoamide]] kinase, mitochondrial OS=Rattus norvegicus OX=10116 GN=Bckdk PE=1 SV=2	1	2	46.4	43	→	0.04	↑	0.4
P70483	Strn	Striatin OS=Rattus norvegicus OX=10116 GN=Strn PE=1 SV=1	4	7	86.2	71	→	0.03	↑	0.42
Q4KM65	Nudt21	Cleavage and polyadenylation specificity factor subunit 5 OS=Rattus norvegicus OX=10116 GN=Nudt21 PE=2 SV=1	4	7	26.2	116	→	0.03	↑	0.42
Q5KTC7	Naaa	N-acylethanolamine-hydrolyzing acid amidase OS=Rattus norvegicus OX=10116 GN=Naaa PE=1 SV=1	4	18	40.3	188	→	0.03	↓	-0.47
Q5PQL5	Ptdss1	Phosphatidylserine synthase 1 OS=Rattus norvegicus OX=10116 GN=Ptdss1 PE=2 SV=1	1	1	55.6	0	→	0.02	↑	0.55
Q923I8	Cry2	Cryptochrome-2 OS=Rattus norvegicus OX=10116 GN=Cry2 PE=1 SV=1	1	2	67.2	24	→	0.01	↑	0.54
P18265	Gsk3a	Glycogen synthase kinase-3 alpha OS=Rattus norvegicus OX=10116 GN=Gsk3a PE=1 SV=1	1	2	51	19	→	0.01	↑	0.38
O89040	Plcb2	1-phosphatidylinositol 4,5-bisphosphate phosphodiesterase beta-2 OS=Rattus norvegicus OX=10116 GN=Plcb2 PE=2 SV=1	3	5	134.8	36	→	0	↑	0.44
P01026	C3	Complement C3 OS=Rattus norvegicus OX=10116 GN=C3 PE=1 SV=3	66	633	186.3	9254	→	0	↓	-0.5
P86411	Ralgapa2	Ral GTPase-activating protein subunit alpha-2 OS=Rattus norvegicus OX=10116 GN=Ralgapa2 PE=1 SV=1	1	2	210.2	23	→	-0.01	↑	0.38
O54889	Polr1a	DNA-directed RNA polymerase I subunit RPA1 OS=Rattus norvegicus OX=10116 GN=Polr1a PE=1 SV=1	1	2	194.1	0	→	-0.01	↓	-0.47
Q9ET20	Scamp4	Secretory carrier-associated membrane protein 4 OS=Rattus norvegicus OX=10116 GN=Scamp4 PE=2 SV=1	1	5	25.5	63	→	-0.02	↑	0.38
Q6IMF1	Krt80	Keratin, type II cytoskeletal 80 OS=Rattus norvegicus OX=10116 GN=Krt80 PE=3 SV=1	1	2	50.5	67	→	-0.02	↓	-0.38
Q6IE52	Mug2	Murinoglobulin-2 OS=Rattus norvegicus OX=10116 GN=Mug2 PE=1 SV=1	3	388	161.5	7150	→	-0.02	↓	-0.52
Q4V8E9	Nrif1	Neurotrophin receptor-interacting factor 1 OS=Rattus norvegicus OX=10116 GN=Nrif1 PE=2 SV=1	1	1	93.6	0	→	-0.02	↓	-0.65
O88588	Pacs1	Phosphofurin acidic cluster sorting protein 1 OS=Rattus norvegicus OX=10116 GN=Pacs1 PE=1 SV=1	5	20	104.6	97	→	-0.03	↑	0.47
Q5PQL2	Cnot9	CCR4-NOT transcription complex subunit 9 OS=Rattus norvegicus OX=10116 GN=Cnot9 PE=1 SV=1	3	4	33.6	52	→	-0.04	↑	0.42
O35815	Atxn3	Ataxin-3 OS=Rattus norvegicus OX=10116 GN=Atxn3 PE=1 SV=1	3	4	40.4	21	→	-0.04	↑	0.52
Q5RJR8	Lrrc59	Leucine-rich repeat-containing protein 59 OS=Rattus norvegicus OX=10116 GN=Lrrc59 PE=1 SV=1	4	22	34.8	507	→	-0.05	↑	0.43

P33124	Acsl6	Long-chain-fatty-acid--CoA ligase 6 OS=Rattus norvegicus OX=10116 GN=Acsl6 PE=1 SV=1	1	7	78.1	47	⇨	-0.05	⇧	0.4
Q5M9G1	Hexim1	Protein HEXIM1 OS=Rattus norvegicus OX=10116 GN=Hexim1 PE=1 SV=1	1	2	40.3	0	⇨	-0.05	⇩	-0.55
Q5XI96	Rnaseh2b	Ribonuclease H2 subunit B OS=Rattus norvegicus OX=10116 GN=Rnaseh2b PE=2 SV=1	1	1	34.6	31	⇨	-0.06	⇩	-0.44
P97594	Mcpt8	Mast cell protease 8 OS=Rattus norvegicus OX=10116 GN=Mcpt8 PE=2 SV=1	1	6	27.5	90	⇨	-0.06	⇩	-1.16
P86410	Ralgapb	Ral GTPase-activating protein subunit beta OS=Rattus norvegicus OX=10116 GN=Ralgapb PE=1 SV=1	3	4	165.3	40	⇨	-0.07	⇧	0.39
Q64591	Decr1	2,4-dienoyl-CoA reductase, mitochondrial OS=Rattus norvegicus OX=10116 GN=Decr1 PE=1 SV=2	6	26	36.1	443	⇨	-0.07	⇩	-0.39
P15589	Sts	Steryl-sulfatase OS=Rattus norvegicus OX=10116 GN=Sts PE=1 SV=2	2	3	62.6	32	⇨	-0.08	⇧	0.39
Q68FP9	Cog6	Conserved oligomeric Golgi complex subunit 6 OS=Rattus norvegicus OX=10116 GN=Cog6 PE=2 SV=1	3	5	72.9	57	⇨	-0.08	⇩	-0.42
P37199	Nup155	Nuclear pore complex protein Nup155 OS=Rattus norvegicus OX=10116 GN=Nup155 PE=1 SV=1	6	12	154.9	90	⇨	-0.09	⇧	0.51
Q8R2H5	Gpld1	Phosphatidylinositol-glycan-specific phospholipase D OS=Rattus norvegicus OX=10116 GN=Gpld1 PE=2 SV=1	5	16	93.7	51	⇨	-0.09	⇩	-0.39
Q5U4E6	Golga4	Golgin subfamily A member 4 OS=Rattus norvegicus OX=10116 GN=Golga4 PE=1 SV=2	6	12	260	70	⇨	-0.1	⇩	-0.6
Q4KLN6	Rrm2	Ribonucleoside-diphosphate reductase subunit M2 OS=Rattus norvegicus OX=10116 GN=Rrm2 PE=2 SV=1	1	1	45	0	⇨	-0.1	⇩	-0.45
Q4FZY0	Efhd2	EF-hand domain-containing protein D2 OS=Rattus norvegicus OX=10116 GN=Efhd2 PE=1 SV=1	5	10	26.7	194	⇨	-0.1	⇩	-0.4
Q6P6Q2	Krt5	Keratin, type II cytoskeletal 5 OS=Rattus norvegicus OX=10116 GN=Krt5 PE=1 SV=1	1	21	61.8	373	⇨	-0.11	⇧	0.76
Q6P7A9	Gaa	Lysosomal alpha-glucosidase OS=Rattus norvegicus OX=10116 GN=Gaa PE=2 SV=1	3	4	106.1	0	⇨	-0.12	⇧	0.45
Q91Y78	Uchl3	Ubiquitin carboxyl-terminal hydrolase isozyme L3 OS=Rattus norvegicus OX=10116 GN=Uchl3 PE=1 SV=1	4	12	26.1	194	⇨	-0.12	⇩	-0.4
P21704	Dnase1	Deoxyribonuclease-1 OS=Rattus norvegicus OX=10116 GN=Dnase1 PE=2 SV=1	1	3	32	24	⇨	-0.13	⇩	-0.55
P97584	Ptgr1	Prostaglandin reductase 1 OS=Rattus norvegicus OX=10116 GN=Ptgr1 PE=2 SV=3	4	18	35.7	333	⇨	-0.14	⇩	-0.44
Q68FT1	Coq9	Ubiquinone biosynthesis protein COQ9, mitochondrial OS=Rattus norvegicus OX=10116 GN=Coq9 PE=1 SV=2	1	2	35.1	36	⇨	-0.15	⇩	-0.41
Q9Z2L0	Vdac1	Voltage-dependent anion-selective channel protein 1 OS=Rattus norvegicus OX=10116 GN=Vdac1 PE=1 SV=4	8	58	30.7	1925	⇨	-0.17	⇩	-0.42
O08697	Arl2	ADP-ribosylation factor-like protein 2 OS=Rattus norvegicus OX=10116 GN=Arl2 PE=1 SV=1	3	10	20.8	220	⇨	-0.17	⇧	0.52
P97924	Kalrn	Kalirin OS=Rattus norvegicus OX=10116 GN=Kalrn PE=1 SV=3	1	7	336.4	28	⇨	-0.17	⇩	-0.74
Q4QR85	Wdr77	Methylosome protein 50 OS=Rattus norvegicus OX=10116 GN=Wdr77 PE=1 SV=1	2	7	37.1	155	⇨	-0.2	⇧	0.43
Q6UPE0	Chdh	Choline dehydrogenase, mitochondrial OS=Rattus norvegicus OX=10116 GN=Chdh PE=1 SV=1	2	6	66.3	63	⇨	-0.2	⇩	-0.45

Q5JCS6	Sipa1l2	Signal-induced proliferation-associated 1-like protein 2 OS=Rattus norvegicus OX=10116 GN=Sipa1l2 PE=1 SV=1	1	5	189.5	26	⇨	-0.21	⇩	-0.4
Q99J86	Atrn	Attractin OS=Rattus norvegicus OX=10116 GN=Atrn PE=2 SV=1	1	3	158.6	26	⇨	-0.21	⇩	-0.56
P20059	Hpx	Hemopexin OS=Rattus norvegicus OX=10116 GN=Hpx PE=1 SV=3	21	537	51.3	11888	⇨	-0.21	⇩	-0.5
P70470	Lypla1	Acyl-protein thioesterase 1 OS=Rattus norvegicus OX=10116 GN=Lypla1 PE=1 SV=1	4	22	24.7	368	⇨	-0.22	⇩	-0.4
Q9WUW3	Cfi	Complement factor I OS=Rattus norvegicus OX=10116 GN=Cfi PE=2 SV=1	3	5	67.3	42	⇨	-0.22	⇩	-0.38
O54735	Pde5a	cGMP-specific 3',5'-cyclic phosphodiesterase OS=Rattus norvegicus OX=10116 GN=Pde5a PE=2 SV=1	13	32	94.5	317	⇨	-0.22	⇩	-0.39
P97852	Hsd17b4	Peroxisomal multifunctional enzyme type 2 OS=Rattus norvegicus OX=10116 GN=Hsd17b4 PE=1 SV=3	19	62	79.4	976	⇨	-0.22	⇩	-0.45
P35559	Ide	Insulin-degrading enzyme OS=Rattus norvegicus OX=10116 GN=Ide PE=1 SV=1	10	31	117.6	332	⇨	-0.22	⇩	-0.43
F1LQ70	Alox12	Arachidonate 12-lipoxygenase, 12S-type OS=Rattus norvegicus OX=10116 GN=Alox12 PE=1 SV=1	4	10	75.5	98	⇨	-0.23	⇩	-0.58
Q63159	Coq3	Ubiquinone biosynthesis O-methyltransferase, mitochondrial OS=Rattus norvegicus OX=10116 GN=Coq3 PE=2 SV=2	1	1	38.7	29	⇨	-0.23	⇩	-0.47
P28494	Man2a1	Alpha-mannosidase 2 OS=Rattus norvegicus OX=10116 GN=Man2a1 PE=1 SV=2	7	16	131.2	140	⇨	-0.26	⇧	0.47
P05544	Serpina3l	Serine protease inhibitor A3L OS=Rattus norvegicus OX=10116 GN=Serpina3l PE=1 SV=3	9	151	46.2	2410	⇨	-0.26	⇩	-0.42
P08426	Try3	Cationic trypsin-3 OS=Rattus norvegicus OX=10116 GN=Try3 PE=2 SV=1	3	9	26.3	44	⇨	-0.26	⇩	-0.91
D3ZAR1	Ldlrap1	Low density lipoprotein receptor adapter protein 1 OS=Rattus norvegicus OX=10116 GN=Ldlrap1 PE=1 SV=2	1	2	33.8	15	⇨	-0.28	⇩	-0.63
P51647	Aldh1a1	Retinal dehydrogenase 1 OS=Rattus norvegicus OX=10116 GN=Aldh1a1 PE=1 SV=3	12	310	54.4	6315	⇨	-0.28	⇩	-0.41
Q63400	Cldn3	Claudin-3 OS=Rattus norvegicus OX=10116 GN=Cldn3 PE=1 SV=2	2	7	23.3	92	⇨	-0.29	⇩	-0.44
P08430	Ugt1a6	UDP-glucuronosyltransferase 1-6 OS=Rattus norvegicus OX=10116 GN=Ugt1a6 PE=1 SV=1	3	9	60.1	39	⇨	-0.29	⇩	-0.42
Q5U2Z3	Nap1l4	Nucleosome assembly protein 1-like 4 OS=Rattus norvegicus OX=10116 GN=Nap1l4 PE=1 SV=1	5	35	43.9	471	⇨	-0.29	⇩	-0.44
Q63041	A1m	Alpha-1-macroglobulin OS=Rattus norvegicus OX=10116 GN=A1m PE=1 SV=1	41	529	167	9699	⇨	-0.29	⇩	-0.79
P39032	Rpl36	60S ribosomal protein L36 OS=Rattus norvegicus OX=10116 GN=Rpl36 PE=1 SV=2	2	5	12.3	121	⇨	-0.3	⇩	-0.44
P18297	Spr	Sepiapterin reductase OS=Rattus norvegicus OX=10116 GN=Spr PE=1 SV=1	5	22	28.1	526	⇨	-0.31	⇩	-0.52
P30349	Lta4h	Leukotriene A-4 hydrolase OS=Rattus norvegicus OX=10116 GN=Lta4h PE=2 SV=2	20	135	69.1	1896	⇨	-0.32	⇩	-0.38
P17988	Sult1a1	Sulfotransferase 1A1 OS=Rattus norvegicus OX=10116 GN=Sult1a1 PE=1 SV=1	7	21	33.9	147	⇨	-0.33	⇩	-0.51
P47967	Lgals5	Galectin-5 OS=Rattus norvegicus OX=10116 GN=Lgals5 PE=1 SV=2	4	23	16.2	350	⇨	-0.33	⇧	0.52
Q6AYT0	Cryz	Quinone oxidoreductase OS=Rattus norvegicus OX=10116 GN=Cryz PE=2 SV=1	2	2	35	0	⇨	-0.34	⇩	-0.49
P55213	Casp3	Caspase-3 OS=Rattus norvegicus OX=10116 GN=Casp3 PE=2 SV=2	1	3	31.5	88	⇨	-0.34	⇩	-0.48
P62832	Rpl23	60S ribosomal protein L23 OS=Rattus norvegicus OX=10116 GN=Rpl23 PE=2 SV=1	6	35	14.9	659	⇨	-0.34	⇩	-0.69
O35293	Cyp2f2	Cytochrome P450 2F2 OS=Rattus norvegicus OX=10116 GN=Cyp2f2 PE=2 SV=1	5	9	55.9	58	⇨	-0.34	⇩	-0.54
Q03626	Mug1	Murinoglobulin-1 OS=Rattus norvegicus OX=10116 GN=Mug1 PE=2 SV=1	8	561	165.2	9728	⇨	-0.35	⇩	-0.85

Q63767	Bcar1	Breast cancer anti-estrogen resistance protein 1 OS=Rattus norvegicus OX=10116 GN=Bcar1 PE=1 SV=1	2	5	104.2	33	→ -0.37	↓ -0.51
P06766	Polb	DNA polymerase beta OS=Rattus norvegicus OX=10116 GN=Polb PE=1 SV=4	1	2	38.3	0	→ -0.37	↓ -0.47
Q6AYS4	Fuca2	Plasma alpha-L-fucosidase OS=Rattus norvegicus OX=10116 GN=Fuca2 PE=2 SV=1	1	1	53.2	0	→ -0.37	↓ -0.63
P35859	Igfals	Insulin-like growth factor-binding protein complex acid labile subunit OS=Rattus norvegicus OX=10116 GN=Igfals PE=1 SV=1	4	20	66.8	509	→ -0.37	↓ -0.51
Q5XIM4	Dmac2l	ATP synthase subunit s, mitochondrial OS=Rattus norvegicus OX=10116 GN=Dmac2l PE=2 SV=1	1	1	23.3	0	→ -0.37	↑ 0.64
P62982	Rps27a	Ubiquitin-40S ribosomal protein S27a OS=Rattus norvegicus OX=10116 GN=Rps27a PE=1 SV=2	4	26	17.9	416	↓ -0.38	→ 0.06
P62752	Rpl23a	60S ribosomal protein L23a OS=Rattus norvegicus OX=10116 GN=Rpl23a PE=2 SV=1	7	40	17.7	535	↓ -0.38	→ -0.13
C9WPN6	Eif2s3y	Eukaryotic translation initiation factor 2 subunit 3, Y-linked OS=Rattus norvegicus OX=10116 GN=Eif2s3y PE=2 SV=2	1	22	51.1	475	↓ -0.38	→ -0.25
Q8K1P9	Fads3	Fatty acid desaturase 3 OS=Rattus norvegicus OX=10116 GN=Fads3 PE=1 SV=1	2	5	51.4	90	↓ -0.38	→ -0.1
P18445	Rpl27a	60S ribosomal protein L27a OS=Rattus norvegicus OX=10116 GN=Rpl27a PE=1 SV=3	3	40	16.6	741	↓ -0.38	→ -0.2
Q99P39	Nfs1	Cysteine desulfurase, mitochondrial OS=Rattus norvegicus OX=10116 GN=Nfs1 PE=2 SV=1	3	6	50	97	↓ -0.38	→ -0.34
O55171	Acot2	Acyl-coenzyme A thioesterase 2, mitochondrial OS=Rattus norvegicus OX=10116 GN=Acot2 PE=1 SV=1	4	49	49.7	917	↓ -0.38	↓ -0.47
P62078	Timm8b	Mitochondrial import inner membrane translocase subunit Tim8 B OS=Rattus norvegicus OX=10116 GN=Timm8b PE=3 SV=1	1	1	9.3	32	↓ -0.38	↓ -0.48
Q6P7B0	Wars	Tryptophan--tRNA ligase, cytoplasmic OS=Rattus norvegicus OX=10116 GN=Wars PE=1 SV=2	12	65	54.1	829	↓ -0.38	→ -0.29
Q9Z1A5	Nae1	NEDD8-activating enzyme E1 regulatory subunit OS=Rattus norvegicus OX=10116 GN=Nae1 PE=1 SV=1	2	4	60.3	60	↓ -0.38	→ -0.33
P63255	Crip1	Cysteine-rich protein 1 OS=Rattus norvegicus OX=10116 GN=Crip1 PE=1 SV=2	1	5	8.5	21	↓ -0.39	→ 0.2
Q794E4	Hnrnpf	Heterogeneous nuclear ribonucleoprotein F OS=Rattus norvegicus OX=10116 GN=Hnrnpf PE=1 SV=3	5	96	45.7	1643	↓ -0.39	→ -0.11
Q4KM62	Palmd	Palmdelphin OS=Rattus norvegicus OX=10116 GN=Palmd PE=1 SV=1	4	8	62.4	49	↓ -0.39	→ 0.22
Q8CHN3	Wfdc2	WAP four-disulfide core domain protein 2 OS=Rattus norvegicus OX=10116 GN=Wfdc2 PE=2 SV=1	1	1	17.4	41	↓ -0.39	→ -0.22
Q9R064	Gorasp2	Golgi reassembly-stacking protein 2 OS=Rattus norvegicus OX=10116 GN=Gorasp2 PE=1 SV=3	1	2	47.2	42	↓ -0.39	→ 0.03
P17164	Fuca1	Tissue alpha-L-fucosidase OS=Rattus norvegicus OX=10116 GN=Fuca1 PE=1 SV=1	4	7	53.5	79	↓ -0.39	→ -0.29
Q63495	Ager	Advanced glycosylation end product-specific receptor OS=Rattus norvegicus OX=10116 GN=Ager PE=1 SV=1	9	182	42.6	2915	↓ -0.39	↓ -0.48
Q5HZX7	Ldah	Lipid droplet-associated hydrolase OS=Rattus norvegicus OX=10116 GN=Ldah PE=2 SV=1	1	1	36.9	20	↓ -0.39	→ -0.27

Q9R085	Usp15	Ubiquitin carboxyl-terminal hydrolase 15 OS=Rattus norvegicus OX=10116 GN=Usp15 PE=1 SV=1	7	14	109.2	76	↓	-0.39	→	0.18
P34058	Hsp90ab1	Heat shock protein HSP 90-beta OS=Rattus norvegicus OX=10116 GN=Hsp90ab1 PE=1 SV=4	20	471	83.2	9441	↓	-0.39	→	-0.03
B0BNN3	Ca1	Carbonic anhydrase 1 OS=Rattus norvegicus OX=10116 GN=Ca1 PE=1 SV=1	11	211	28.3	3950	↓	-0.39	→	-0.25
Q6PEC0	Nudt2	Bis(5'-nucleosyl)-tetraphosphatase [asymmetrical] OS=Rattus norvegicus OX=10116 GN=Nudt2 PE=2 SV=3	3	9	16.9	209	↓	-0.39	→	-0.21
P16228	Ctse	Cathepsin E OS=Rattus norvegicus OX=10116 GN=Ctse PE=1 SV=3	2	4	43	29	↓	-0.4	→	-0.28
P13803	Etfa	Electron transfer flavoprotein subunit alpha, mitochondrial OS=Rattus norvegicus OX=10116 GN=Etfa PE=1 SV=4	8	51	34.9	1112	↓	-0.4	→	-0.25
B0BNL6	Arrdc1	Arrestin domain-containing protein 1 OS=Rattus norvegicus OX=10116 GN=Arrdc1 PE=1 SV=1	3	7	46.1	57	↓	-0.4	→	0.11
O35783	Calu	Calumenin OS=Rattus norvegicus OX=10116 GN=Calu PE=1 SV=1	2	12	37	99	↓	-0.4	→	-0.28
P36970	Gpx4	Phospholipid hydroperoxide glutathione peroxidase OS=Rattus norvegicus OX=10116 GN=Gpx4 PE=1 SV=3	3	10	22.2	172	↓	-0.4	→	-0.24
D4A4T9	Chordc1	Cysteine and histidine-rich domain-containing protein 1 OS=Rattus norvegicus OX=10116 GN=Chordc1 PE=3 SV=1	4	8	37.3	170	↓	-0.4	→	-0.31
Q8CF97	Vcpip1	Deubiquitinating protein VCIP135 OS=Rattus norvegicus OX=10116 GN=Vcpip1 PE=1 SV=2	1	1	134.5	26	↓	-0.4	↓	-0.4
Q566Q8	Bles03	UPF0696 protein C11orf68 homolog OS=Rattus norvegicus OX=10116 GN=Bles03 PE=1 SV=2	3	11	31.4	145	↓	-0.4	→	0.01
Q5XIS1	Ssh3	Protein phosphatase Slingshot homolog 3 OS=Rattus norvegicus OX=10116 GN=Ssh3 PE=1 SV=1	1	4	72	55	↓	-0.4	→	-0.12
P62250	Rps16	40S ribosomal protein S16 OS=Rattus norvegicus OX=10116 GN=Rps16 PE=1 SV=2	13	83	16.4	820	↓	-0.41	→	-0.11
O88377	Pip4k2b	Phosphatidylinositol 5-phosphate 4-kinase type-2 beta OS=Rattus norvegicus OX=10116 GN=Pip4k2b PE=1 SV=1	2	4	47.2	23	↓	-0.41	→	0.02
Q6MG61	Clic1	Chloride intracellular channel protein 1 OS=Rattus norvegicus OX=10116 GN=Clic1 PE=1 SV=1	8	56	27	929	↓	-0.41	→	-0.29
Q8VHZ8	Dscam	Down syndrome cell adhesion molecule homolog OS=Rattus norvegicus OX=10116 GN=Dscam PE=1 SV=1	1	4	222.1	0	↓	-0.41	→	-0.29
P27867	Sord	Sorbitol dehydrogenase OS=Rattus norvegicus OX=10116 GN=Sord PE=1 SV=4	4	13	38.2	207	↓	-0.41	→	-0.28
P0C2X9	Aldh4a1	Delta-1-pyrroline-5-carboxylate dehydrogenase, mitochondrial OS=Rattus norvegicus OX=10116 GN=Aldh4a1 PE=1 SV=1	11	51	61.8	860	↓	-0.41	↓	-0.47
P50393	Pla2g4a	Cytosolic phospholipase A2 OS=Rattus norvegicus OX=10116 GN=Pla2g4a PE=1 SV=1	2	7	85.7	34	↓	-0.41	→	-0.16
P51593	Huwe1	E3 ubiquitin-protein ligase HUWE1 (Fragment) OS=Rattus norvegicus OX=10116 GN=Huwe1 PE=1 SV=2	1	2	37.3	24	↓	-0.41	↓	-0.43
P06757	Adh1	Alcohol dehydrogenase 1 OS=Rattus norvegicus OX=10116 GN=Adh1 PE=1 SV=3	4	29	39.6	449	↓	-0.41	↓	-0.39

B2GV38	Ubl4a	Ubiquitin-like protein 4A OS=Rattus norvegicus OX=10116 GN=Ubl4a PE=2 SV=1	1	1	17.8	31	↓	-0.42	→	0.08
P63174	Rpl38	60S ribosomal protein L38 OS=Rattus norvegicus OX=10116 GN=Rpl38 PE=1 SV=2	2	4	8.2	78	↓	-0.42	↓	-0.56
D3ZFJ3	Sh3bp1	SH3 domain-binding protein 1 OS=Rattus norvegicus OX=10116 GN=Sh3bp1 PE=1 SV=1	3	5	74.8	43	↓	-0.42	↑	0.6
Q9JJ09	Slc34a2	Sodium-dependent phosphate transport protein 2B OS=Rattus norvegicus OX=10116 GN=Slc34a2 PE=1 SV=1	4	20	75.9	501	↓	-0.42	→	-0.12
P04550	Ptms	Parathymosin OS=Rattus norvegicus OX=10116 GN=Ptms PE=1 SV=2	2	14	11.6	531	↓	-0.42	→	0.06
P84850	D2hgdh	D-2-hydroxyglutarate dehydrogenase, mitochondrial OS=Rattus norvegicus OX=10116 GN=D2hgdh PE=3 SV=1	4	10	58.8	20	↓	-0.42	→	-0.32
F1LQY6	Necab2	N-terminal EF-hand calcium-binding protein 2 OS=Rattus norvegicus OX=10116 GN=Necab2 PE=1 SV=3	1	1	43.5	0	↓	-0.43	→	0.16
P47198	Rpl22	60S ribosomal protein L22 OS=Rattus norvegicus OX=10116 GN=Rpl22 PE=2 SV=2	1	12	14.8	419	↓	-0.43	↓	-0.4
P62271	Rps18	40S ribosomal protein S18 OS=Rattus norvegicus OX=10116 GN=Rps18 PE=1 SV=3	10	51	17.7	738	↓	-0.43	→	-0.1
P85834	Tufm	Elongation factor Tu, mitochondrial OS=Rattus norvegicus OX=10116 GN=Tufm PE=1 SV=1	13	66	49.5	1012	↓	-0.43	→	-0.24
P62914	Rpl11	60S ribosomal protein L11 OS=Rattus norvegicus OX=10116 GN=Rpl11 PE=1 SV=2	5	31	20.2	409	↓	-0.43	→	-0.08
B2RYD6	Ttc30b	Tetratricopeptide repeat protein 30B OS=Rattus norvegicus OX=10116 GN=Ttc30b PE=2 SV=1	3	4	76	87	↓	-0.43	→	-0.25
Q9EPJ0	Nucks1	Nuclear ubiquitous casein and cyclin-dependent kinase substrate 1 OS=Rattus norvegicus OX=10116 GN=Nucks1 PE=1 SV=1	2	7	27.1	131	↓	-0.44	↓	-0.4
Q5FVJ0	Rufy3	Protein RUFY3 OS=Rattus norvegicus OX=10116 GN=Rufy3 PE=1 SV=1	3	6	52.9	38	↓	-0.44	→	0.1
P62893	Rpl39	60S ribosomal protein L39 OS=Rattus norvegicus OX=10116 GN=Rpl39 PE=1 SV=2	1	2	6.4	21	↓	-0.44	↓	-0.49
P35763	Prf1	Perforin-1 OS=Rattus norvegicus OX=10116 GN=Prf1 PE=2 SV=1	3	5	61.5	76	↓	-0.44	→	0.13
O88370	Pip4k2c	Phosphatidylinositol 5-phosphate 4-kinase type-2 gamma OS=Rattus norvegicus OX=10116 GN=Pip4k2c PE=1 SV=1	2	3	47	35	↓	-0.44	↓	-0.45
Q9EPB1	Dpp7	Dipeptidyl peptidase 2 OS=Rattus norvegicus OX=10116 GN=Dpp7 PE=1 SV=1	4	12	55.1	94	↓	-0.44	↓	-0.39
Q5PPN4	Ca8	Carbonic anhydrase-related protein OS=Rattus norvegicus OX=10116 GN=Ca8 PE=1 SV=3	1	10	32.9	92	↓	-0.44	→	-0.25
Q499U2	Elmo3	Engulfment and cell motility protein 3 OS=Rattus norvegicus OX=10116 GN=Elmo3 PE=2 SV=1	1	2	81.6	39	↓	-0.44	→	-0.23
P01805		Ig heavy chain V region IR2 OS=Rattus norvegicus OX=10116 PE=4 SV=1	2	7	16	41	↓	-0.44	→	-0.32
Q8VHF5	Cs	Citrate synthase, mitochondrial OS=Rattus norvegicus OX=10116 GN=Cs PE=1 SV=1	10	84	51.8	1862	↓	-0.44	↓	-0.42
P07483	Fabp3	Fatty acid-binding protein, heart OS=Rattus norvegicus OX=10116 GN=Fabp3 PE=1 SV=2	1	6	14.8	94	↓	-0.45	→	0.07
P17077	Rpl9	60S ribosomal protein L9 OS=Rattus norvegicus OX=10116 GN=Rpl9 PE=1 SV=1	3	17	21.9	346	↓	-0.45	→	-0.29
Q5NDL0	Eogt	EGF domain-specific O-linked N-acetylglucosamine transferase OS=Rattus norvegicus OX=10116 GN=Eogt PE=2 SV=1	4	9	61.5	65	↓	-0.45	→	-0.3
P04916	Rbp4	Retinol-binding protein 4 OS=Rattus norvegicus OX=10116 GN=Rbp4 PE=1 SV=1	3	23	23.2	410	↓	-0.45	→	-0.16
Q91XS8	Stk17b	Serine/threonine-protein kinase 17B OS=Rattus norvegicus OX=10116 GN=Stk17b PE=1 SV=1	1	3	42.1	41	↓	-0.45	↓	-0.49

Q6IG02	Krt2	Keratin, type II cytoskeletal 2 epidermal OS=Rattus norvegicus OX=10116 GN=Krt2 PE=3 SV=1	3	13	69.1	222	↓	-0.45	↓	-0.4
Q6IFW6	Krt10	Keratin, type I cytoskeletal 10 OS=Rattus norvegicus OX=10116 GN=Krt10 PE=3 SV=1	10	104	56.5	1258	↓	-0.45	↓	-0.47
O08949	Gtf2a1	Transcription initiation factor IIA subunit 1 OS=Rattus norvegicus OX=10116 GN=Gtf2a1 PE=1 SV=1	1	1	41.5	22	↓	-0.46	→	0.24
Q498U4	Sarnp	SAP domain-containing ribonucleoprotein OS=Rattus norvegicus OX=10116 GN=Sarnp PE=1 SV=3	1	6	23.6	189	↓	-0.46	→	-0.35
Q8VD52	Pdxp	Pyridoxal phosphate phosphatase OS=Rattus norvegicus OX=10116 GN=Pdxp PE=1 SV=2	5	21	33.1	238	↓	-0.46	→	0.1
Q63507	Rpl14	60S ribosomal protein L14 OS=Rattus norvegicus OX=10116 GN=Rpl14 PE=1 SV=3	5	23	23.3	451	↓	-0.46	→	-0.17
P55159	Pon1	Serum paraoxonase/arylesterase 1 OS=Rattus norvegicus OX=10116 GN=Pon1 PE=1 SV=3	5	16	39.3	150	↓	-0.46	↓	-0.51
P35704	Prdx2	Peroxiredoxin-2 OS=Rattus norvegicus OX=10116 GN=Prdx2 PE=1 SV=3	7	144	21.8	2725	↓	-0.46	→	-0.19
Q5U2N0	Ctps2	CTP synthase 2 OS=Rattus norvegicus OX=10116 GN=Ctps2 PE=1 SV=1	2	8	65.6	34	↓	-0.46	→	-0.07
B1H234	Flrt3	Leucine-rich repeat transmembrane protein FLRT3 OS=Rattus norvegicus OX=10116 GN=Flrt3 PE=1 SV=1	2	3	72.9	55	↓	-0.47	→	0.08
Q4KLL0	Tcea1	Transcription elongation factor A protein 1 OS=Rattus norvegicus OX=10116 GN=Tcea1 PE=1 SV=1	3	4	33.9	78	↓	-0.47	↓	-0.64
D3ZF92	Tnfrsf21	Tumor necrosis factor receptor superfamily member 21 OS=Rattus norvegicus OX=10116 GN=Tnfrsf21 PE=1 SV=1	1	2	71.8	0	↓	-0.47	→	0.09
P70531	Eef2k	Eukaryotic elongation factor 2 kinase OS=Rattus norvegicus OX=10116 GN=Eef2k PE=1 SV=1	3	8	81.4	74	↓	-0.47	→	-0.1
P62912	Rpl32	60S ribosomal protein L32 OS=Rattus norvegicus OX=10116 GN=Rpl32 PE=1 SV=2	4	14	15.9	215	↓	-0.47	→	-0.16
B2RYG6	Otub1	Ubiquitin thioesterase OTUB1 OS=Rattus norvegicus OX=10116 GN=Otub1 PE=1 SV=1	4	10	31.3	240	↓	-0.47	→	-0.03
P70541	Eif2b3	Translation initiation factor eIF-2B subunit gamma OS=Rattus norvegicus OX=10116 GN=Eif2b3 PE=2 SV=2	4	14	50.4	152	↓	-0.47	→	-0.21
Q62639	Rheb	GTP-binding protein Rheb OS=Rattus norvegicus OX=10116 GN=Rheb PE=1 SV=1	4	16	20.5	200	↓	-0.47	→	-0.35
Q9WVK3	Pecr	Peroxisomal trans-2-enoyl-CoA reductase OS=Rattus norvegicus OX=10116 GN=Pecr PE=2 SV=1	6	27	32.4	543	↓	-0.47	↓	-0.57
P62282	Rps11	40S ribosomal protein S11 OS=Rattus norvegicus OX=10116 GN=Rps11 PE=1 SV=3	5	35	18.4	487	↓	-0.48	→	-0.18
P09006	Serpina3n	Serine protease inhibitor A3N OS=Rattus norvegicus OX=10116 GN=Serpina3n PE=1 SV=3	11	60	46.6	971	↓	-0.48	↓	-0.96
P63159	Hmgb1	High mobility group protein B1 OS=Rattus norvegicus OX=10116 GN=Hmgb1 PE=1 SV=2	11	46	24.9	539	↓	-0.48	↓	-0.47
Q8K4D8	Aldh1a3	Aldehyde dehydrogenase family 1 member A3 OS=Rattus norvegicus OX=10116 GN=Aldh1a3 PE=2 SV=1	4	23	56.1	283	↓	-0.48	→	-0.25
Q6IRK9	Cpq	Carboxypeptidase Q OS=Rattus norvegicus OX=10116 GN=Cpq PE=1 SV=1	5	16	52	390	↓	-0.48	↓	-0.72
P62902	Rpl31	60S ribosomal protein L31 OS=Rattus norvegicus OX=10116 GN=Rpl31 PE=2 SV=1	7	33	14.5	580	↓	-0.49	→	-0.06
P62919	Rpl8	60S ribosomal protein L8 OS=Rattus norvegicus OX=10116 GN=Rpl8 PE=2 SV=2	11	48	28	475	↓	-0.49	→	-0.34
P62703	Rps4x	40S ribosomal protein S4, X isoform OS=Rattus norvegicus OX=10116 GN=Rps4x PE=2 SV=2	12	86	29.6	1183	↓	-0.49	→	-0.21

P48199	Crp	C-reactive protein OS=Rattus norvegicus OX=10116 GN=Crp PE=1 SV=1	2	7	25.5	193	↓	-0.49	↓	-0.56
Q499T7	Cfap20	Cilia- and flagella-associated protein 20 OS=Rattus norvegicus OX=10116 GN=Cfap20 PE=2 SV=1	1	1	19.1	17	↓	-0.49	→	0.01
P83645	Gpx2	Glutathione peroxidase 2 OS=Rattus norvegicus OX=10116 GN=Gpx2 PE=1 SV=3	9	43	22	462	↓	-0.49	↓	-0.47
Q9QXL7	Nme7	Nucleoside diphosphate kinase 7 OS=Rattus norvegicus OX=10116 GN=Nme7 PE=1 SV=1	1	2	44.5	39	↓	-0.49	→	0.11
P62828	Ran	GTP-binding nuclear protein Ran OS=Rattus norvegicus OX=10116 GN=Ran PE=1 SV=3	7	60	24.4	849	↓	-0.49	→	-0.29
Q6AYA1	Gar1	H/ACA ribonucleoprotein complex subunit 1 OS=Rattus norvegicus OX=10116 GN=Gar1 PE=2 SV=1	1	1	23	21	↓	-0.49	→	0.2
Q5BJP5	Tmem230	Transmembrane protein 230 OS=Rattus norvegicus OX=10116 GN=Tmem230 PE=1 SV=1	1	3	13.2	18	↓	-0.49	→	-0.33
P21263	Nes	Nestin OS=Rattus norvegicus OX=10116 GN=Nes PE=1 SV=2	1	2	208.7	0	↓	-0.49	→	-0.28
P62856	Rps26	40S ribosomal protein S26 OS=Rattus norvegicus OX=10116 GN=Rps26 PE=3 SV=3	2	13	13	404	↓	-0.5	↓	-0.43
F1LMY4	Ryr1	Ryanodine receptor 1 OS=Rattus norvegicus OX=10116 GN=Ryr1 PE=1 SV=1	1	4	565.1	48	↓	-0.5	↓	-0.42
Q08290	Cnn1	Calponin-1 OS=Rattus norvegicus OX=10116 GN=Cnn1 PE=1 SV=1	4	44	33.3	876	↓	-0.5	→	0.01
Q6P734	Serping1	Plasma protease C1 inhibitor OS=Rattus norvegicus OX=10116 GN=Serping1 PE=2 SV=1	11	39	55.6	761	↓	-0.5	↓	-0.57
P49911	Anp32a	Acidic leucine-rich nuclear phosphoprotein 32 family member A OS=Rattus norvegicus OX=10116 GN=Anp32a PE=2 SV=1	2	28	28.5	383	↓	-0.5	↓	-0.49
Q68FX9	Sirt5	NAD-dependent protein deacylase sirtuin-5, mitochondrial OS=Rattus norvegicus OX=10116 GN=Sirt5 PE=2 SV=1	1	1	34.1	0	↓	-0.5	→	0.02
Q99P55	Sgpp1	Sphingosine-1-phosphate phosphatase 1 OS=Rattus norvegicus OX=10116 GN=Sgpp1 PE=1 SV=2	1	1	47.6	0	↓	-0.51	→	0.32
P17702	Rpl28	60S ribosomal protein L28 OS=Rattus norvegicus OX=10116 GN=Rpl28 PE=1 SV=4	7	40	15.8	433	↓	-0.51	→	-0.13
Q99MC0	Ppp1r14a	Protein phosphatase 1 regulatory subunit 14A OS=Rattus norvegicus OX=10116 GN=Ppp1r14a PE=1 SV=1	3	14	16.7	236	↓	-0.52	↓	-0.38
Q6TXG9	Sfr1	Swi5-dependent recombination DNA repair protein 1 homolog OS=Rattus norvegicus OX=10116 GN=Sfr1 PE=2 SV=1	2	5	28.7	51	↓	-0.52	↓	-0.65
P62142	Ppp1cb	Serine/threonine-protein phosphatase PP1-beta catalytic subunit OS=Rattus norvegicus OX=10116 GN=Ppp1cb PE=1 SV=3	2	34	37.2	409	↓	-0.52	→	-0.05
Q8VHK0	Acot8	Acyl-coenzyme A thioesterase 8 OS=Rattus norvegicus OX=10116 GN=Acot8 PE=1 SV=1	1	1	36	21	↓	-0.52	↑	0.57
Q6PDV7	Rpl10	60S ribosomal protein L10 OS=Rattus norvegicus OX=10116 GN=Rpl10 PE=1 SV=3	2	8	24.6	45	↓	-0.52	↓	-0.43
P29826	RT1-Bb	Rano class II histocompatibility antigen, B-1 beta chain OS=Rattus norvegicus OX=10116 GN=RT1-Bb PE=2 SV=1	4	11	29.9	67	↓	-0.52	↓	-0.46
Q80W83	Ppp2r5b	Serine/threonine-protein phosphatase 2A 56 kDa regulatory subunit beta isoform OS=Rattus norvegicus OX=10116 GN=Ppp2r5b PE=2 SV=1	1	5	57.3	42	↓	-0.52	→	-0.01
Q3KRE0	Atad3	ATPase family AAA domain-containing protein 3 OS=Rattus norvegicus OX=10116 GN=Atad3 PE=1 SV=1	3	5	66.7	54	↓	-0.52	→	-0.22
P27008	Parp1	Poly [ADP-ribose] polymerase 1 OS=Rattus norvegicus OX=10116 GN=Parp1 PE=1 SV=4	5	8	112.6	95	↓	-0.53	→	-0.26

P62850	Rps24	40S ribosomal protein S24 OS=Rattus norvegicus OX=10116 GN=Rps24 PE=2 SV=1	6	24	15.4	282	↓	-0.55	→	-0.24
P62268	Rps23	40S ribosomal protein S23 OS=Rattus norvegicus OX=10116 GN=Rps23 PE=1 SV=3	5	17	15.8	451	↓	-0.55	↓	-0.47
P52925	Hmgb2	High mobility group protein B2 OS=Rattus norvegicus OX=10116 GN=Hmgb2 PE=2 SV=2	3	4	24.1	0	↓	-0.55	→	-0.35
P14046	A1i3	Alpha-1-inhibitor 3 OS=Rattus norvegicus OX=10116 GN=A1i3 PE=1 SV=1	10	671	163.7	11625	↓	-0.55	↓	-0.81
P62755	Rps6	40S ribosomal protein S6 OS=Rattus norvegicus OX=10116 GN=Rps6 PE=1 SV=1	7	50	28.7	821	↓	-0.55	→	-0.35
Q68FW7	Tars2	Threonine--tRNA ligase, mitochondrial OS=Rattus norvegicus OX=10116 GN=Tars2 PE=2 SV=1	2	8	81.6	128	↓	-0.56	→	-0.01
P62246	Rps15a	40S ribosomal protein S15a OS=Rattus norvegicus OX=10116 GN=Rps15a PE=1 SV=2	6	36	14.8	342	↓	-0.56	↓	-0.38
Q66H15	Rmdn3	Regulator of microtubule dynamics protein 3 OS=Rattus norvegicus OX=10116 GN=Rmdn3 PE=1 SV=1	1	2	52.3	0	↓	-0.56	↓	-0.56
P04644	Rps17	40S ribosomal protein S17 OS=Rattus norvegicus OX=10116 GN=Rps17 PE=1 SV=3	1	2	15.5	77	↓	-0.56	↓	-0.49
Q9WVH8	Fbln5	Fibulin-5 OS=Rattus norvegicus OX=10116 GN=Fbln5 PE=2 SV=1	4	39	50.1	578	↓	-0.56	↓	-0.48
P02767	Ttr	Transthyretin OS=Rattus norvegicus OX=10116 GN=Ttr PE=1 SV=1	4	36	15.7	726	↓	-0.57	↓	-0.51
O35094	Timm44	Mitochondrial import inner membrane translocase subunit TIM44 OS=Rattus norvegicus OX=10116 GN=Timm44 PE=1 SV=1	2	5	51	68	↓	-0.57	→	-0.04
P61751	Arf4	ADP-ribosylation factor 4 OS=Rattus norvegicus OX=10116 GN=Arf4 PE=2 SV=2	2	26	20.4	724	↓	-0.57	→	-0.36
Q68FP2	Pon3	Serum paraoxonase/lactonase 3 OS=Rattus norvegicus OX=10116 GN=Pon3 PE=2 SV=1	6	54	39.4	849	↓	-0.58	→	-0.3
Q62703	Rcn2	Reticulocalbin-2 OS=Rattus norvegicus OX=10116 GN=Rcn2 PE=1 SV=2	1	2	37.4	18	↓	-0.58	↓	-0.4
P62243	Rps8	40S ribosomal protein S8 OS=Rattus norvegicus OX=10116 GN=Rps8 PE=1 SV=2	4	45	24.2	574	↓	-0.58	→	-0.24
P04041	Gpx1	Glutathione peroxidase 1 OS=Rattus norvegicus OX=10116 GN=Gpx1 PE=1 SV=4	10	91	22.3	1502	↓	-0.6	↓	-0.42
P48675	Des	Desmin OS=Rattus norvegicus OX=10116 GN=Des PE=1 SV=2	14	117	53.4	1746	↓	-0.6	↓	-1.02
Q03238	Gzmm	Granzyme M (Fragment) OS=Rattus norvegicus OX=10116 GN=Gzmm PE=1 SV=1	2	6	28.3	32	↓	-0.61	→	-0.17
P25886	Rpl29	60S ribosomal protein L29 OS=Rattus norvegicus OX=10116 GN=Rpl29 PE=1 SV=3	4	24	17.3	269	↓	-0.62	→	-0.24
Q63616	Vps33b	Vacuolar protein sorting-associated protein 33B OS=Rattus norvegicus OX=10116 GN=Vps33b PE=2 SV=1	6	14	70.6	93	↓	-0.62	↓	-0.41
P49242	Rps3a	40S ribosomal protein S3a OS=Rattus norvegicus OX=10116 GN=Rps3a PE=1 SV=2	10	89	29.9	831	↓	-0.62	→	-0.29
I6L9G5	Rcn3	Reticulocalbin-3 OS=Rattus norvegicus OX=10116 GN=Rcn3 PE=1 SV=1	1	2	37.9	29	↓	-0.62	↓	-0.45
Q9JKA8	Hcn3	Potassium/sodium hyperpolarization-activated cyclic nucleotide-gated channel 3 OS=Rattus norvegicus OX=10116 GN=Hcn3 PE=1 SV=1	1	2	86.8	0	↓	-0.62	↓	-0.43
O35986	Zranb2	Zinc finger Ran-binding domain-containing protein 2 OS=Rattus norvegicus OX=10116 GN=Zranb2 PE=1 SV=2	1	1	37.3	39	↓	-0.63	→	-0.32
P24049	Rpl17	60S ribosomal protein L17 OS=Rattus norvegicus OX=10116 GN=Rpl17 PE=2 SV=3	6	45	21.4	580	↓	-0.63	↓	-0.38
P84083	Arf5	ADP-ribosylation factor 5 OS=Rattus norvegicus OX=10116 GN=Arf5 PE=1 SV=2	3	49	20.5	1127	↓	-0.64	→	-0.31
Q32Q05	Yod1	Ubiquitin thioesterase OTU1 OS=Rattus norvegicus OX=10116 GN=Yod1 PE=2 SV=1	3	13	37.4	200	↓	-0.64	→	-0.31
P24050	Rps5	40S ribosomal protein S5 OS=Rattus norvegicus OX=10116 GN=Rps5 PE=1 SV=3	4	28	22.9	343	↓	-0.65	→	-0.2

O88637	Pcyt2	Ethanolamine-phosphate cytidylyltransferase OS=Rattus norvegicus OX=10116 GN=Pcyt2 PE=1 SV=1	2	6	45.2	106	↓	-0.65	→	-0.18
Q9Z1T4	Cnksr2	Connector enhancer of kinase suppressor of ras 2 OS=Rattus norvegicus OX=10116 GN=Cnksr2 PE=1 SV=1	1	2	117.3	0	↓	-0.66	→	-0.29
D4A3K5	Hist1h1a	Histone H1.1 OS=Rattus norvegicus OX=10116 GN=Hist1h1a PE=1 SV=1	2	20	22	415	↓	-0.67	↓	-0.39
Q6AXS3	Dek	Protein DEK OS=Rattus norvegicus OX=10116 GN=Dek PE=1 SV=1	1	6	42.9	99	↓	-0.68	↓	-0.51
O55165	Kif3c	Kinesin-like protein KIF3C OS=Rattus norvegicus OX=10116 GN=Kif3c PE=2 SV=1	1	2	89.8	44	↓	-0.68	→	-0.06
P57093	Phyh	Phytanoyl-CoA dioxygenase, peroxisomal OS=Rattus norvegicus OX=10116 GN=Phyh PE=1 SV=2	1	1	38.6	25	↓	-0.68	→	-0.12
P09117	Aldoc	Fructose-bisphosphate aldolase C OS=Rattus norvegicus OX=10116 GN=Aldoc PE=1 SV=3	3	31	39.3	189	↓	-0.7	↓	-1.19
P41123	Rpl13	60S ribosomal protein L13 OS=Rattus norvegicus OX=10116 GN=Rpl13 PE=1 SV=2	8	55	24.3	894	↓	-0.71	→	-0.31
P62845	Rps15	40S ribosomal protein S15 OS=Rattus norvegicus OX=10116 GN=Rps15 PE=1 SV=2	3	7	17	30	↓	-0.71	→	-0.32
Q64294	Pdpn	Podoplanin OS=Rattus norvegicus OX=10116 GN=Pdpn PE=1 SV=1	1	2	17.6	0	↓	-0.71	→	0.2
Q9JK71	Magi3	Membrane-associated guanylate kinase, WW and PDZ domain-containing protein 3 OS=Rattus norvegicus OX=10116 GN=Magi3 PE=1 SV=2	7	15	160.5	188	↓	-0.72	→	0.25
Q9JK72	Ccs	Copper chaperone for superoxide dismutase OS=Rattus norvegicus OX=10116 GN=Ccs PE=1 SV=1	3	9	28.9	195	↓	-0.72	→	-0.23
P61928	Rpl37	60S ribosomal protein L37 OS=Rattus norvegicus OX=10116 GN=Rpl37 PE=1 SV=2	1	2	11.1	0	↓	-0.73	↓	-0.53
P11250	Rpl34	60S ribosomal protein L34 OS=Rattus norvegicus OX=10116 GN=Rpl34 PE=1 SV=3	5	31	13.5	325	↓	-0.73	↓	-0.53
P63326	Rps10	40S ribosomal protein S10 OS=Rattus norvegicus OX=10116 GN=Rps10 PE=2 SV=1	6	45	18.9	570	↓	-0.73	↓	-0.4
Q6AY19	Coq8b	Atypical kinase COQ8B, mitochondrial OS=Rattus norvegicus OX=10116 GN=Coq8b PE=1 SV=1	2	5	58.9	33	↓	-0.74	→	-0.21
Q5U211	Snx3	Sorting nexin-3 OS=Rattus norvegicus OX=10116 GN=Snx3 PE=1 SV=1	6	34	18.8	401	↓	-0.75	↓	-0.54
P20812	Cyp2a3	Cytochrome P450 2A3 OS=Rattus norvegicus OX=10116 GN=Cyp2a3 PE=2 SV=1	4	6	56.5	86	↓	-0.75	↓	-0.57
P83883	Rpl36a	60S ribosomal protein L36a OS=Rattus norvegicus OX=10116 GN=Rpl36a PE=1 SV=2	3	12	12.4	127	↓	-0.77	↓	-0.6
P61212	Arl1	ADP-ribosylation factor-like protein 1 OS=Rattus norvegicus OX=10116 GN=Arl1 PE=1 SV=1	1	4	20.4	130	↓	-0.78	→	-0.28
O08700	Vps45	Vacuolar protein sorting-associated protein 45 OS=Rattus norvegicus OX=10116 GN=Vps45 PE=1 SV=1	6	12	64.9	96	↓	-0.78	↓	-0.4
Q9JI92	Sdcbp	Syntenin-1 OS=Rattus norvegicus OX=10116 GN=Sdcbp PE=1 SV=1	1	5	32.4	35	↓	-0.78	↓	-0.86
Q6VV72	Eif1a	Eukaryotic translation initiation factor 1A OS=Rattus norvegicus OX=10116 GN=Eif1a PE=2 SV=3	2	3	16.5	23	↓	-0.79	→	-0.37
O35263	Pafah1b3	Platelet-activating factor acetylhydrolase IB subunit gamma OS=Rattus norvegicus OX=10116 GN=Pafah1b3 PE=2 SV=1	2	4	25.8	52	↓	-0.8	→	-0.14
Q4FZU2	Krt6a	Keratin, type II cytoskeletal 6A OS=Rattus norvegicus OX=10116 GN=Krt6a PE=1 SV=1	1	19	59.2	462	↓	-0.8	↓	-0.44
Q07803	Gfm1	Elongation factor G, mitochondrial OS=Rattus norvegicus OX=10116 GN=Gfm1 PE=1 SV=2	5	10	83.4	85	↓	-0.82	↓	-0.52
P50463	Csrp3	Cysteine and glycine-rich protein 3 OS=Rattus norvegicus OX=10116 GN=Csrp3 PE=1 SV=1	2	7	20.8	87	↓	-0.82	↓	-1.01

P62083	Rps7	40S ribosomal protein S7 OS=Rattus norvegicus OX=10116 GN=Rps7 PE=1 SV=1	5	26	22.1	417	↓	-0.83	↓	-0.73
P83732	Rpl24	60S ribosomal protein L24 OS=Rattus norvegicus OX=10116 GN=Rpl24 PE=2 SV=1	6	28	17.8	438	↓	-0.83	↓	-0.85
P17074	Rps19	40S ribosomal protein S19 OS=Rattus norvegicus OX=10116 GN=Rps19 PE=2 SV=3	8	61	16.1	1227	↓	-0.86	→	-0.36
P02634	S100g	Protein S100-G OS=Rattus norvegicus OX=10116 GN=S100g PE=1 SV=3	2	3	9	122	↓	-0.87	↑	0.65
Q5PQJ7	Tbcel	Tubulin-specific chaperone cofactor E-like protein OS=Rattus norvegicus OX=10116 GN=Tbcel PE=1 SV=1	1	2	48	21	↓	-0.87	→	-0.03
P00770	Mcpt2	Mast cell protease 2 OS=Rattus norvegicus OX=10116 GN=Mcpt2 PE=1 SV=1	1	3	27.1	27	↓	-0.89	↓	-1.47
D3ZBN0	Hist1h1b	Histone H1.5 OS=Rattus norvegicus OX=10116 GN=Hist1h1b PE=1 SV=1	4	25	22.6	466	↓	-0.91	→	-0.36
P31016	Dlg4	Disks large homolog 4 OS=Rattus norvegicus OX=10116 GN=Dlg4 PE=1 SV=1	1	3	80.4	31	↓	-0.92	↓	-0.82
G3V801	Prss12	Neurotrypsin OS=Rattus norvegicus OX=10116 GN=Prss12 PE=1 SV=1	2	3	84.2	0	↓	-0.95	↓	-0.51
P13471	Rps14	40S ribosomal protein S14 OS=Rattus norvegicus OX=10116 GN=Rps14 PE=2 SV=3	3	40	16.2	871	↓	-0.98	↓	-0.74
F1M3G7	Akap13	A-kinase anchor protein 13 OS=Rattus norvegicus OX=10116 GN=Akap13 PE=1 SV=2	1	1	301.2	0	↓	-1.03	↓	-0.47
Q923V4	Fbxo6	F-box only protein 6 OS=Rattus norvegicus OX=10116 GN=Fbxo6 PE=1 SV=1	5	11	32.8	93	↓	-1.07	→	0.12
P62853	Rps25	40S ribosomal protein S25 OS=Rattus norvegicus OX=10116 GN=Rps25 PE=2 SV=1	6	45	13.7	759	↓	-1.13	↓	-0.78
Q6AXS5	Serbp1	Plasminogen activator inhibitor 1 RNA-binding protein OS=Rattus norvegicus OX=10116 GN=Serbp1 PE=1 SV=2	2	6	44.7	171	↓	-1.31	↓	-0.87
P62864	Fau	40S ribosomal protein S30 OS=Rattus norvegicus OX=10116 GN=Fau PE=1 SV=1	1	4	6.6	96	↓	-1.35	↓	-1.39
P15865	Hist1h1e	Histone H1.4 OS=Rattus norvegicus OX=10116 GN=Hist1h1e PE=1 SV=3	11	145	22	3464	↓	-1.4	↓	-0.39
P62275	Rps29	40S ribosomal protein S29 OS=Rattus norvegicus OX=10116 GN=Rps29 PE=1 SV=2	1	1	6.7	22	↓	-1.95	↓	-1.84
P01836		Ig kappa chain C region, A allele OS=Rattus norvegicus OX=10116 PE=1 SV=1	2	91	11.7	1389	↓	-2.94	↓	-3.57

Table S3. The PM-specific DEPs in 3M- and 6M-exposed groups

Accession	Gnen name	Description	#Unique Peptides	#PSMs	MW [kDa]	Mascot Score	3M-PM1/GAS (log_2Ratio)	6M-PM1/GAS (log_2Ratio)
Q6IRI9	Fmo2	Dimethylaniline monooxygenase [N-oxide-forming] 2 OS=Rattus norvegicus OX=10116 GN=	4	23	60.9	204	↑ 2.2	→ 0.07
P08932		T-kininogen 2 OS=Rattus norvegicus OX=10116 PE=1 SV=2	3	128	47.7	2470	↑ 1.32	↑ 0.51
P01048	Map1	T-kininogen 1 OS=Rattus norvegicus OX=10116 GN=Map1 PE=1 SV=2	5	119	47.7	1816	↑ 1.23	↑ 0.49
P09006	Serpina3n	Serine protease inhibitor A3N OS=Rattus norvegicus OX=10116 GN=Serpina3n PE=1 SV=3	11	60	46.6	971	↑ 0.87	↑ 0.38
P01836		Ig kappa chain C region, A allele OS=Rattus norvegicus OX=10116 PE=1 SV=1	2	91	11.7	1389	↑ 0.86	→ 0.23
Q64716	Insrr	Insulin receptor-related protein OS=Rattus norvegicus OX=10116 GN=Insrr PE=1 SV=3	1	2	144.8	0	↑ 0.78	→ 0.15
B0BNK9	Cracr2b	EF-hand calcium-binding domain-containing protein 4A OS=Rattus norvegicus OX=10116 GN	1	1	44.5	0	↑ 0.75	→ 0.2
Q6IFU7	Krt42	Keratin, type I cytoskeletal 42 OS=Rattus norvegicus OX=10116 GN=Krt42 PE=3 SV=1	3	126	50.2	1447	↑ 0.74	→ -0.01
Q04931	Ssrp1	FACT complex subunit SSRP1 OS=Rattus norvegicus OX=10116 GN=Ssrp1 PE=1 SV=2	1	1	80.9	0	↑ 0.73	→ 0.06
P00770	Mcpt2	Mast cell protease 2 OS=Rattus norvegicus OX=10116 GN=Mcpt2 PE=1 SV=1	1	3	27.1	27	↑ 0.68	→ 0.1
Q5XI60	Reep6	Receptor expression-enhancing protein 6 OS=Rattus norvegicus OX=10116 GN=Reep6 PE=2	1	7	23.3	16	↑ 0.66	→ -0.15
P31721	C1qb	Complement C1q subcomponent subunit B OS=Rattus norvegicus OX=10116 GN=C1qb PE=1	1	3	26.6	25	↑ 0.66	↑ 0.42
Q63041	A1m	Alpha-1-macroglobulin OS=Rattus norvegicus OX=10116 GN=A1m PE=1 SV=1	41	529	167	9699	↑ 0.64	→ 0.14
Q68FX7	Thoc5	THO complex subunit 5 homolog OS=Rattus norvegicus OX=10116 GN=Thoc5 PE=2 SV=1	1	1	78.6	30	↑ 0.58	→ 0.07
Q62640	Grid1	Glutamate receptor ionotropic, delta-1 OS=Rattus norvegicus OX=10116 GN=Grid1 PE=2 SV=	1	1	112.1	18	↑ 0.58	↓ -1.61
Q6TRW4	Pds5b	Sister chromatid cohesion protein PDS5 homolog B OS=Rattus norvegicus OX=10116 GN=Pds	2	8	164.4	90	↑ 0.57	→ 0.17
P08649	C4	Complement C4 OS=Rattus norvegicus OX=10116 GN=C4 PE=1 SV=3	44	220	192	3558	↑ 0.56	→ 0.15
Q9ERC5	Otof	Otoferlin OS=Rattus norvegicus OX=10116 GN=Otof PE=1 SV=2	1	1	226.2	28	↑ 0.54	→ 0.35
A0JPN3	Bpifb1	BPI fold-containing family B member 1 OS=Rattus norvegicus OX=10116 GN=Bpifb1 PE=2 SV	1	1	52.2	0	↑ 0.53	↑ 0.47
Q6IE52	Mug2	Murinoglobulin-2 OS=Rattus norvegicus OX=10116 GN=Mug2 PE=1 SV=1	3	388	161.5	7150	↑ 0.52	→ 0.02
P08650	C5	Complement C5 (Fragment) OS=Rattus norvegicus OX=10116 GN=C5 PE=1 SV=2	1	4	9	51	↑ 0.52	→ 0.21
P01026	C3	Complement C3 OS=Rattus norvegicus OX=10116 GN=C3 PE=1 SV=3	66	633	186.3	9254	↑ 0.51	→ 0.02
Q63089	Slc22a1	Solute carrier family 22 member 1 OS=Rattus norvegicus OX=10116 GN=Slc22a1 PE=1 SV=1	1	1	61.5	0	↑ 0.5	→ 0.11
Q9ES71	Gnpat	Dihydroxyacetone phosphate acyltransferase OS=Rattus norvegicus OX=10116 GN=Gnpat P	3	7	77	88	↑ 0.49	→ -0.24
O88267	Acot1	Acyl-coenzyme A thioesterase 1 OS=Rattus norvegicus OX=10116 GN=Acot1 PE=1 SV=1	1	26	46	504	↑ 0.48	→ 0.25
P62275	Rps29	40S ribosomal protein S29 OS=Rattus norvegicus OX=10116 GN=Rps29 PE=1 SV=2	1	1	6.7	22	↑ 0.47	↑ 0.58
P20059	Hpx	Hemopexin OS=Rattus norvegicus OX=10116 GN=Hpx PE=1 SV=3	21	537	51.3	11888	↑ 0.46	→ 0.16
Q63416	Itih3	Inter-alpha-trypsin inhibitor heavy chain H3 OS=Rattus norvegicus OX=10116 GN=Itih3 PE=2	12	86	99	1229	↑ 0.46	→ -0.01
Q499N3	Wdr18	WD repeat-containing protein 18 OS=Rattus norvegicus OX=10116 GN=Wdr18 PE=2 SV=1	2	3	47.2	53	↑ 0.45	↑ 0.39
Q62975	Serpina10	Protein Z-dependent protease inhibitor OS=Rattus norvegicus OX=10116 GN=Serpina10 PE=	4	16	50.2	117	↑ 0.45	→ 0.13
Q9R1J8	P3h1	Prolyl 3-hydroxylase 1 OS=Rattus norvegicus OX=10116 GN=P3h1 PE=1 SV=1	1	2	82.3	16	↑ 0.44	↓ -0.38
Q06606	Mcpt10	Granzyme-like protein 2 OS=Rattus norvegicus OX=10116 GN=Mcpt10 PE=2 SV=1	1	5	27.4	54	↑ 0.44	→ -0.24
Q03626	Mug1	Murinoglobulin-1 OS=Rattus norvegicus OX=10116 GN=Mug1 PE=2 SV=1	8	561	165.2	9728	↑ 0.44	→ -0.06
P20761	Igh-1a	Ig gamma-2B chain C region OS=Rattus norvegicus OX=10116 GN=Igh-1a PE=1 SV=1	7	107	36.5	1759	↑ 0.44	→ 0.13
Q6AYT0	Cryz	Quinone oxidoreductase OS=Rattus norvegicus OX=10116 GN=Cryz PE=2 SV=1	2	2	35	0	↑ 0.43	→ 0.28

D4A1J4	Bdh2	3-hydroxybutyrate dehydrogenase type 2 OS=Rattus norvegicus OX=10116 GN=Bdh2 PE=3 S	1	5	26.6	95	↑	0.43	→	0.14
O35293	Cyp2f2	Cytochrome P450 2F2 OS=Rattus norvegicus OX=10116 GN=Cyp2f2 PE=2 SV=1	5	9	55.9	58	↑	0.43	→	0.23
Q64240	Ambp	Protein AMBP OS=Rattus norvegicus OX=10116 GN=Ambp PE=1 SV=1	3	14	38.8	153	↑	0.42	→	-0.03
Q99PS8	Hrg	Histidine-rich glycoprotein OS=Rattus norvegicus OX=10116 GN=Hrg PE=1 SV=1	11	67	59	1111	↑	0.42	→	0.09
P21704	Dnase1	Deoxyribonuclease-1 OS=Rattus norvegicus OX=10116 GN=Dnase1 PE=2 SV=1	1	3	32	24	↑	0.41	→	0
P17988	Sult1a1	Sulfotransferase 1A1 OS=Rattus norvegicus OX=10116 GN=Sult1a1 PE=1 SV=1	7	21	33.9	147	↑	0.4	→	0.22
Q9JLT0	Myh10	Myosin-10 OS=Rattus norvegicus OX=10116 GN=Myh10 PE=1 SV=1	7	58	228.8	613	↑	0.4	→	0.24
Q08464	Fzd2	Frizzled-2 OS=Rattus norvegicus OX=10116 GN=Fzd2 PE=1 SV=1	2	3	63.8	16	↑	0.4	→	0.09
Q6P689	Wls	Protein wntless homolog OS=Rattus norvegicus OX=10116 GN=Wls PE=1 SV=1	1	3	62.2	17	↑	0.4	→	0.19
P20767		Ig lambda-2 chain C region OS=Rattus norvegicus OX=10116 PE=4 SV=1	5	45	11.3	1199	↑	0.4	→	-0.02
P06399	Fga	Fibrinogen alpha chain OS=Rattus norvegicus OX=10116 GN=Fga PE=1 SV=3	21	224	86.6	3301	↑	0.39	→	0.29
O88453	Safb	Scaffold attachment factor B1 OS=Rattus norvegicus OX=10116 GN=Safb PE=1 SV=2	2	3	104.5	59	↑	0.39	→	0.03
P13635	Cp	Ceruloplasmin OS=Rattus norvegicus OX=10116 GN=Cp PE=1 SV=3	25	255	120.8	4560	↑	0.39	→	0.13
Q5PPH0	Enoph1	Enolase-phosphatase E1 OS=Rattus norvegicus OX=10116 GN=Enoph1 PE=2 SV=1	2	8	28.9	40	↑	0.39	→	-0.22
Q63518	Mybpc1	Myosin-binding protein C, slow-type (Fragment) OS=Rattus norvegicus OX=10116 GN=Mybp	2	3	68.7	33	↑	0.39	↓	-1.66
Q6P734	Serping1	Plasma protease C1 inhibitor OS=Rattus norvegicus OX=10116 GN=Serping1 PE=2 SV=1	11	39	55.6	761	↑	0.38	→	0.3
P97594	Mcpt8	Mast cell protease 8 OS=Rattus norvegicus OX=10116 GN=Mcpt8 PE=2 SV=1	1	6	27.5	90	↑	0.38	↓	-0.72
Q6AYI5	Shoc2	Leucine-rich repeat protein SHOC-2 OS=Rattus norvegicus OX=10116 GN=Shoc2 PE=2 SV=1	1	1	64.9	0	→	0.35	↑	2.1
P50609	Fmod	Fibromodulin OS=Rattus norvegicus OX=10116 GN=Fmod PE=2 SV=1	2	10	43.2	224	→	0.3	↑	0.39
Q6AXS5	Serbp1	Plasminogen activator inhibitor 1 RNA-binding protein OS=Rattus norvegicus OX=10116 GN=	2	6	44.7	171	→	0.25	↑	0.69
Q6AY80	Nqo2	Ribosyldihydronicotinamide dehydrogenase [quinone] OS=Rattus norvegicus OX=10116 GN=	5	17	26.3	244	→	0.25	↓	-0.4
Q6AY87	Thoc6	THO complex subunit 6 homolog OS=Rattus norvegicus OX=10116 GN=Thoc6 PE=2 SV=1	2	4	37.4	40	→	0.21	↑	0.42
P21263	Nes	Nestin OS=Rattus norvegicus OX=10116 GN=Nes PE=1 SV=2	1	2	208.7	0	→	0.2	↑	0.41
P51556	Dgka	Diacylglycerol kinase alpha OS=Rattus norvegicus OX=10116 GN=Dgka PE=2 SV=1	3	8	82.1	80	→	0.18	↑	0.42
Q6MGA9	Brd2	Bromodomain-containing protein 2 OS=Rattus norvegicus OX=10116 GN=Brd2 PE=1 SV=1	1	1	88	48	→	0.17	↓	-0.4
Q6IMF3	Krt1	Keratin, type II cytoskeletal 1 OS=Rattus norvegicus OX=10116 GN=Krt1 PE=2 SV=1	3	22	64.8	305	→	0.17	↑	0.46
Q9JJP9	Ubqln1	Ubiquilin-1 OS=Rattus norvegicus OX=10116 GN=Ubqln1 PE=1 SV=1	1	1	62	0	→	0.17	↓	-0.46
Q9ER30	Klhl41	Kelch-like protein 41 OS=Rattus norvegicus OX=10116 GN=Klhl41 PE=1 SV=1	1	1	68.2	0	→	0.17	↓	-0.82
P04466	Mylpf	Myosin regulatory light chain 2, skeletal muscle isoform OS=Rattus norvegicus OX=10116 GI	3	15	19	41	→	0.16	↓	-2.63
Q5RJY4	Dhrs7b	Dehydrogenase/reductase SDR family member 7B OS=Rattus norvegicus OX=10116 GN=Dh	2	3	35.3	30	→	0.16	↑	1.99
O35986	Zranb2	Zinc finger Ran-binding domain-containing protein 2 OS=Rattus norvegicus OX=10116 GN=Zr	1	1	37.3	39	→	0.15	↑	0.46
P07756	Cps1	Carbamoyl-phosphate synthase [ammonia], mitochondrial OS=Rattus norvegicus OX=10116	1	5	164.5	72	→	0.15	↑	0.52
Q5RKH1	Prpf4b	Serine/threonine-protein kinase PRP4 homolog OS=Rattus norvegicus OX=10116 GN=Prpf4b	1	1	116.9	25	→	0.15	↓	-2.05
Q32PX2	Aimp2	Aminoacyl tRNA synthase complex-interacting multifunctional protein 2 OS=Rattus norvegicu	3	13	35.4	115	→	0.15	↓	-1.32
P36201	Crip2	Cysteine-rich protein 2 OS=Rattus norvegicus OX=10116 GN=Crip2 PE=2 SV=1	3	19	22.7	166	→	0.14	↑	0.55
Q4G074	Kbp	KIF1-binding protein OS=Rattus norvegicus OX=10116 GN=Kbp PE=2 SV=1	1	4	71.3	64	→	0.13	↓	-1.28
P47864	Aqp5	Aquaporin-5 OS=Rattus norvegicus OX=10116 GN=Aqp5 PE=2 SV=1	3	12	28.4	252	→	0.12	↓	-0.77
P97924	Kalrn	Kalirin OS=Rattus norvegicus OX=10116 GN=Kalrn PE=1 SV=3	1	7	336.4	28	→	0.1	↓	-0.48
P16290	Pgam2	Phosphoglycerate mutase 2 OS=Rattus norvegicus OX=10116 GN=Pgam2 PE=1 SV=2	3	28	28.7	592	→	0.1	↓	-1.44

P21571	Atp5pf	ATP synthase-coupling factor 6, mitochondrial OS=Rattus norvegicus OX=10116 GN=Atp5pf I	1	2	12.5	31	→	0.09	↑	0.41
Q5KTC7	Naaa	N-acylethanolamine-hydrolyzing acid amidase OS=Rattus norvegicus OX=10116 GN=Naaa P	4	18	40.3	188	→	0.09	↓	-0.41
Q5U4E6	Golga4	Golgin subfamily A member 4 OS=Rattus norvegicus OX=10116 GN=Golga4 PE=1 SV=2	6	12	260	70	→	0.08	↓	-0.42
Q63862	Myh11	Myosin-11 (Fragments) OS=Rattus norvegicus OX=10116 GN=Myh11 PE=1 SV=3	16	93	152.4	1123	→	0.07	↑	0.45
P12847	Myh3	Myosin-3 OS=Rattus norvegicus OX=10116 GN=Myh3 PE=3 SV=1	6	19	223.7	79	→	0.07	↓	-2.19
P11883	Aldh3a1	Aldehyde dehydrogenase, dimeric NADP-preferring OS=Rattus norvegicus OX=10116 GN=Al	5	21	50.3	251	→	0.06	↓	-0.58
Q0H8B9	Clec2d11	C-type lectin domain family 2 member D11 OS=Rattus norvegicus OX=10116 GN=Clec2d11 F	4	29	23.5	528	→	0.05	↓	-1.42
P51868	Casq2	Calsequestrin-2 OS=Rattus norvegicus OX=10116 GN=Casq2 PE=1 SV=2	3	4	47.8	21	→	0.05	↓	-0.61
Q4V8E9	Nrif1	Neurotrophin receptor-interacting factor 1 OS=Rattus norvegicus OX=10116 GN=Nrif1 PE=2 S	1	1	93.6	0	→	0.03	↓	-0.61
Q5EB81	Cyb5r1	NADH-cytochrome b5 reductase 1 OS=Rattus norvegicus OX=10116 GN=Cyb5r1 PE=2 SV=1	3	4	34.2	17	→	0.02	↓	-0.5
Q4QQS3	Oscp1	Protein OSCP1 OS=Rattus norvegicus OX=10116 GN=Oscp1 PE=2 SV=1	2	4	43.3	23	→	0.02	↑	0.58
Q9QZ76	Mb	Myoglobin OS=Rattus norvegicus OX=10116 GN=Mb PE=1 SV=3	3	15	17.1	287	→	0.02	↓	-1.75
Q6AY31		Uncharacterized protein C4orf36 homolog OS=Rattus norvegicus OX=10116 PE=4 SV=1	1	3	13.3	36	→	0.01	↓	-0.4
Q63118	Calcrl	Calcitonin gene-related peptide type 1 receptor OS=Rattus norvegicus OX=10116 GN=Calcrl F	1	4	53.3	25	→	0.01	↑	0.38
P06907	Mpz	Myelin protein P0 OS=Rattus norvegicus OX=10116 GN=Mpz PE=1 SV=1	1	1	27.6	0	→	0.01	↑	1.02
Q8K4K5	Llgl1	Lethal(2) giant larvae protein homolog 1 OS=Rattus norvegicus OX=10116 GN=Llgl1 PE=1 SV	4	7	112.4	137	→	0.01	↓	-0.4
P25886	Rpl29	60S ribosomal protein L29 OS=Rattus norvegicus OX=10116 GN=Rpl29 PE=1 SV=3	4	24	17.3	269	→	0	↑	0.39
Q9R064	Gorasp2	Golgi reassembly-stacking protein 2 OS=Rattus norvegicus OX=10116 GN=Gorasp2 PE=1 SV=	1	2	47.2	42	→	0	↑	0.42
P19633	Casq1	Calsequestrin-1 OS=Rattus norvegicus OX=10116 GN=Casq1 PE=1 SV=2	1	3	46.4	48	→	0	↓	-1.66
P00564	Ckm	Creatine kinase M-type OS=Rattus norvegicus OX=10116 GN=Ckm PE=1 SV=2	10	99	43	1347	→	0	↓	-2.97
P41123	Rpl13	60S ribosomal protein L13 OS=Rattus norvegicus OX=10116 GN=Rpl13 PE=1 SV=2	8	55	24.3	894	→	-0.01	↑	0.39
P09812	Pygm	Glycogen phosphorylase, muscle form OS=Rattus norvegicus OX=10116 GN=Pygm PE=1 SV=	16	130	97.2	1341	→	-0.01	↓	-1.14
P00884	Aldob	Fructose-bisphosphate aldolase B OS=Rattus norvegicus OX=10116 GN=Aldob PE=1 SV=2	1	8	39.6	59	→	-0.04	↑	0.56
P47858	Pfkm	ATP-dependent 6-phosphofructokinase, muscle type OS=Rattus norvegicus OX=10116 GN=F	5	22	85.5	280	→	-0.04	↓	-0.7
Q920P6	Ada	Adenosine deaminase OS=Rattus norvegicus OX=10116 GN=Ada PE=1 SV=3	11	43	39.9	344	→	-0.04	↓	-1.42
P62632	Eef1a2	Elongation factor 1-alpha 2 OS=Rattus norvegicus OX=10116 GN=Eef1a2 PE=1 SV=1	1	123	50.4	1882	→	-0.04	↓	-1.06
P15429	Eno3	Beta-enolase OS=Rattus norvegicus OX=10116 GN=Eno3 PE=1 SV=3	8	60	47	796	→	-0.04	↓	-2.64
Q4KLN6	Rrm2	Ribonucleoside-diphosphate reductase subunit M2 OS=Rattus norvegicus OX=10116 GN=Rrr	1	1	45	0	→	-0.05	↓	-0.4
P20070	Cyb5r3	NADH-cytochrome b5 reductase 3 OS=Rattus norvegicus OX=10116 GN=Cyb5r3 PE=1 SV=2	8	83	34.2	1269	→	-0.06	↑	0.4
Q6P6Q2	Krt5	Keratin, type II cytoskeletal 5 OS=Rattus norvegicus OX=10116 GN=Krt5 PE=1 SV=1	1	21	61.8	373	→	-0.06	↑	0.81
P05065	Aldoa	Fructose-bisphosphate aldolase A OS=Rattus norvegicus OX=10116 GN=Aldoa PE=1 SV=2	15	225	39.3	4706	→	-0.06	↓	-0.44
P17074	Rps19	40S ribosomal protein S19 OS=Rattus norvegicus OX=10116 GN=Rps19 PE=2 SV=3	8	61	16.1	1227	→	-0.06	↑	0.43
P08426	Try3	Cationic trypsin-3 OS=Rattus norvegicus OX=10116 GN=Try3 PE=2 SV=1	3	9	26.3	44	→	-0.07	↓	-0.72
B1H234	Flrt3	Leucine-rich repeat transmembrane protein FLRT3 OS=Rattus norvegicus OX=10116 GN=Flrt	2	3	72.9	55	→	-0.08	↑	0.47
P97541	Hspb6	Heat shock protein beta-6 OS=Rattus norvegicus OX=10116 GN=Hspb6 PE=1 SV=1	3	6	17.5	47	→	-0.08	↓	-0.39
Q5XXR3	Arhgef6	Rho guanine nucleotide exchange factor 6 OS=Rattus norvegicus OX=10116 GN=Arhgef6 PE=	3	10	87	44	→	-0.08	↓	-0.53
P28494	Man2a1	Alpha-mannosidase 2 OS=Rattus norvegicus OX=10116 GN=Man2a1 PE=1 SV=2	7	16	131.2	140	→	-0.09	↑	0.64
O35550	Rabep1	Rab GTPase-binding effector protein 1 OS=Rattus norvegicus OX=10116 GN=Rabep1 PE=1 S\	5	10	99.4	40	→	-0.09	↓	-0.39
A2RRT9	Cyp4v2	Cytochrome P450 4V2 OS=Rattus norvegicus OX=10116 GN=Cyp4v2 PE=2 SV=1	1	3	60.5	0	→	-0.1	↓	-0.56

P61203	Cops2	COP9 signalosome complex subunit 2 OS=Rattus norvegicus OX=10116 GN=Cops2 PE=1 SV=	9	22	51.6	229	→	-0.1	↓	-0.64
P58775	Tpm2	Tropomyosin beta chain OS=Rattus norvegicus OX=10116 GN=Tpm2 PE=1 SV=1	3	52	32.8	718	→	-0.11	↓	-0.73
P35763	Prf1	Perforin-1 OS=Rattus norvegicus OX=10116 GN=Prf1 PE=2 SV=1	3	5	61.5	76	→	-0.12	↑	0.44
Q6P7A9	Gaa	Lysosomal alpha-glucosidase OS=Rattus norvegicus OX=10116 GN=Gaa PE=2 SV=1	3	4	106.1	0	→	-0.13	↑	0.44
Q8VI02	Ppp4r1	Serine/threonine-protein phosphatase 4 regulatory subunit 1 OS=Rattus norvegicus OX=101	1	1	105.5	0	→	-0.14	↓	-0.49
Q5M7W5	Map4	Microtubule-associated protein 4 OS=Rattus norvegicus OX=10116 GN=Map4 PE=1 SV=1	28	148	110.2	1785	→	-0.15	↑	0.4
Q6AYA1	Gar1	H/ACA ribonucleoprotein complex subunit 1 OS=Rattus norvegicus OX=10116 GN=Gar1 PE=2	1	1	23	21	→	-0.15	↑	0.53
Q02759	Alox15	Arachidonate 15-lipoxygenase OS=Rattus norvegicus OX=10116 GN=Alox15 PE=1 SV=3	2	2	75.3	29	→	-0.16	↑	0.99
Q3SWS9	Jakmip1	Janus kinase and microtubule-interacting protein 1 OS=Rattus norvegicus OX=10116 GN=Jak	1	2	73.1	0	→	-0.17	↑	0.55
Q6AYK6	Cacybp	Calcyclin-binding protein OS=Rattus norvegicus OX=10116 GN=Cacybp PE=1 SV=1	4	22	26.5	285	→	-0.19	↓	-0.52
Q4KM62	Palmd	Palmdelphin OS=Rattus norvegicus OX=10116 GN=Palmd PE=1 SV=1	4	8	62.4	49	→	-0.19	↑	0.42
Q6AXU7	Mif4gd	MIF4G domain-containing protein OS=Rattus norvegicus OX=10116 GN=Mif4gd PE=2 SV=1	2	3	25.5	29	→	-0.2	↓	-0.43
P23928	Cryab	Alpha-crystallin B chain OS=Rattus norvegicus OX=10116 GN=Cryab PE=1 SV=1	10	52	20.1	796	→	-0.2	↓	-0.47
Q8K3P7	Hint3	Histidine triad nucleotide-binding protein 3 OS=Rattus norvegicus OX=10116 GN=Hint3 PE=2	3	16	19.7	151	→	-0.21	↑	0.38
Q5RKJ1	Maea	E3 ubiquitin-protein transferase MAEA OS=Rattus norvegicus OX=10116 GN=Maea PE=2 SV	1	1	45.3	0	→	-0.23	↓	-0.46
P15865	Hist1h1e	Histone H1.4 OS=Rattus norvegicus OX=10116 GN=Hist1h1e PE=1 SV=3	11	145	22	3464	→	-0.23	↑	0.78
Q5XIM4	Dmac2l	ATP synthase subunit s, mitochondrial OS=Rattus norvegicus OX=10116 GN=Dmac2l PE=2 S\	1	1	23.3	0	→	-0.27	↑	0.74
Q66HG3	Cndp1	Beta-Ala-His dipeptidase OS=Rattus norvegicus OX=10116 GN=Cndp1 PE=1 SV=1	2	7	54.9	49	→	-0.28	↓	-0.42
P16409	Myl3	Myosin light chain 3 OS=Rattus norvegicus OX=10116 GN=Myl3 PE=1 SV=2	1	18	22.1	162	→	-0.28	↓	-2.28
Q7M6Z5	Kif27	Kinesin-like protein KIF27 OS=Rattus norvegicus OX=10116 GN=Kif27 PE=1 SV=1	2	3	158.8	0	→	-0.28	↓	-0.39
Q64294	Pdpn	Podoplanin OS=Rattus norvegicus OX=10116 GN=Pdpn PE=1 SV=1	1	2	17.6	0	→	-0.28	↑	0.62
Q64578	Atp2a1	Sarcoplasmic/endoplasmic reticulum calcium ATPase 1 OS=Rattus norvegicus OX=10116 GN	3	58	109.3	723	→	-0.28	↓	-2.56
Q63189	Prg2	Bone marrow proteoglycan OS=Rattus norvegicus OX=10116 GN=Prg2 PE=2 SV=1	1	2	25.1	68	→	-0.31	↓	-0.67
Q6AYP5	Cadm1	Cell adhesion molecule 1 OS=Rattus norvegicus OX=10116 GN=Cadm1 PE=1 SV=1	1	4	51.8	61	→	-0.31	↑	0.39
P61459	Pcbd1	Pterin-4-alpha-carbinolamine dehydratase OS=Rattus norvegicus OX=10116 GN=Pcbd1 PE=1	1	2	12	37	→	-0.31	↓	-0.39
Q9JKL7	Srek1	Splicing regulatory glutamine/lysine-rich protein 1 OS=Rattus norvegicus OX=10116 GN=Srel	1	1	56.8	35	↓	-0.38	→	0.19
P41682	Adh7	Alcohol dehydrogenase class 4 mu/sigma chain OS=Rattus norvegicus OX=10116 GN=Adh7	4	17	40.1	123	↓	-0.38	↓	-0.4
Q9ET20	Scamp4	Secretory carrier-associated membrane protein 4 OS=Rattus norvegicus OX=10116 GN=Scar	1	5	25.5	63	↓	-0.38	→	0.03
Q5FVJ0	Rufy3	Protein RUFY3 OS=Rattus norvegicus OX=10116 GN=Rufy3 PE=1 SV=1	3	6	52.9	38	↓	-0.38	→	0.16
Q9QXY4	Ogfr	Opioid growth factor receptor OS=Rattus norvegicus OX=10116 GN=Ogfr PE=1 SV=1	1	1	64.7	35	↓	-0.38	→	0.14
Q5FVH2	Pld3	Phospholipase D3 OS=Rattus norvegicus OX=10116 GN=Pld3 PE=1 SV=1	5	19	54.4	253	↓	-0.38	→	-0.02
P54275	Msh2	DNA mismatch repair protein Msh2 OS=Rattus norvegicus OX=10116 GN=Msh2 PE=2 SV=1	1	7	104	0	↓	-0.38	→	0.13
P63255	Crip1	Cysteine-rich protein 1 OS=Rattus norvegicus OX=10116 GN=Crip1 PE=1 SV=2	1	5	8.5	21	↓	-0.39	→	0.2
Q91Y81	Septin2	Septin-2 OS=Rattus norvegicus OX=10116 GN=Sept2 PE=1 SV=1	12	74	41.6	1203	↓	-0.39	→	-0.05
Q499T7	Cfap20	Cilia- and flagella-associated protein 20 OS=Rattus norvegicus OX=10116 GN=Cfap20 PE=2 S	1	1	19.1	17	↓	-0.39	→	0.11
Q4L1J4	Magi1	Membrane-associated guanylate kinase, WW and PDZ domain-containing protein 1 OS=Rat	4	7	136.2	45	↓	-0.39	→	-0.12
Q5U2N0	Ctps2	CTP synthase 2 OS=Rattus norvegicus OX=10116 GN=Ctps2 PE=1 SV=1	2	8	65.6	34	↓	-0.39	→	0
Q9WUJ3	Pde4dip	Myomegalin OS=Rattus norvegicus OX=10116 GN=Pde4dip PE=1 SV=1	1	1	261.9	0	↓	-0.4	→	0.15
Q6P6U0	Fgr	Tyrosine-protein kinase Fgr OS=Rattus norvegicus OX=10116 GN=Fgr PE=1 SV=1	2	18	58.8	145	↓	-0.4	→	0.2

Q9QXL7	Nme7	Nucleoside diphosphate kinase 7 OS=Rattus norvegicus OX=10116 GN=Nme7 PE=1 SV=1	1	2	44.5	39	↓	-0.4	⇨	0.2
P19234	Ndufv2	NADH dehydrogenase [ubiquinone] flavoprotein 2, mitochondrial OS=Rattus norvegicus OX=	3	12	27.4	98	↓	-0.41	⇨	-0.03
P01041	Cstb	Cystatin-B OS=Rattus norvegicus OX=10116 GN=Cstb PE=1 SV=1	3	26	11.2	427	↓	-0.41	⇨	-0.19
Q68FY1	Nup35	Nucleoporin NUP35 OS=Rattus norvegicus OX=10116 GN=Nup35 PE=1 SV=1	2	2	34.8	0	↓	-0.41	⇨	-0.15
Q63186	Eif2b4	Translation initiation factor eIF-2B subunit delta OS=Rattus norvegicus OX=10116 GN=Eif2b4	4	7	57.8	128	↓	-0.41	⇨	-0.26
P04550	Ptms	Parathymosin OS=Rattus norvegicus OX=10116 GN=Ptms PE=1 SV=2	2	14	11.6	531	↓	-0.41	⇨	0.07
Q4AE70	Carm1	Histone-arginine methyltransferase CARM1 OS=Rattus norvegicus OX=10116 GN=Carm1 PE	1	2	70.3	58	↓	-0.41	⇨	0.03
Q03555	Gphn	Gephyrin OS=Rattus norvegicus OX=10116 GN=Gphn PE=1 SV=3	1	1	83.2	25	↓	-0.42	⇨	0
P23711	Hmox2	Heme oxygenase 2 OS=Rattus norvegicus OX=10116 GN=Hmox2 PE=1 SV=1	3	4	35.7	89	↓	-0.42	⇨	-0.02
P57093	Phyh	Phytanoyl-CoA dioxygenase, peroxisomal OS=Rattus norvegicus OX=10116 GN=Phyh PE=1 S	1	1	38.6	25	↓	-0.42	⇨	0.13
P09875	Ugt2b1	UDP-glucuronosyltransferase 2B1 OS=Rattus norvegicus OX=10116 GN=Ugt2b1 PE=2 SV=1	2	5	60.4	67	↓	-0.43	⇨	-0.13
F1LQY6	Necab2	N-terminal EF-hand calcium-binding protein 2 OS=Rattus norvegicus OX=10116 GN=Necab2	1	1	43.5	0	↓	-0.43	⇨	0.16
O89040	Plcb2	1-phosphatidylinositol 4,5-bisphosphate phosphodiesterase beta-2 OS=Rattus norvegicus O)	3	5	134.8	36	↓	-0.43	⇨	0.02
D4A770		Protein C12orf4 homolog OS=Rattus norvegicus OX=10116 PE=1 SV=1	2	5	63.6	32	↓	-0.43	⇨	-0.16
O08697	Arl2	ADP-ribosylation factor-like protein 2 OS=Rattus norvegicus OX=10116 GN=Arl2 PE=1 SV=1	3	10	20.8	220	↓	-0.43	⇨	0.26
Q80W83	Ppp2r5b	Serine/threonine-protein phosphatase 2A 56 kDa regulatory subunit beta isoform OS=Rattus	1	5	57.3	42	↓	-0.43	⇨	0.07
Q811U3	Erc1	ELKS/Rab6-interacting/CAST family member 1 OS=Rattus norvegicus OX=10116 GN=Erc1 PE	2	3	108.8	76	↓	-0.44	⇨	-0.08
O88637	Pcyt2	Ethanolamine-phosphate cytidylyltransferase OS=Rattus norvegicus OX=10116 GN=Pcyt2 PI	2	6	45.2	106	↓	-0.44	⇨	0.03
Q07652	Cacna1e	Voltage-dependent R-type calcium channel subunit alpha-1E OS=Rattus norvegicus OX=101	1	3	252	0	↓	-0.45	⇨	0.08
P15589	Sts	Steryl-sulfatase OS=Rattus norvegicus OX=10116 GN=Sts PE=1 SV=2	2	3	62.6	32	↓	-0.45	⇨	0.01
O35795	Entpd2	Ectonucleoside triphosphate diphosphohydrolase 2 OS=Rattus norvegicus OX=10116 GN=En	1	1	54.4	0	↓	-0.45	↓	-0.53
Q5M7T2	Spryd7	SPRY domain-containing protein 7 OS=Rattus norvegicus OX=10116 GN=Spryd7 PE=2 SV=1	2	3	21.7	76	↓	-0.46	⇨	-0.13
Q9JIL8	Rad50	DNA repair protein RAD50 OS=Rattus norvegicus OX=10116 GN=Rad50 PE=1 SV=1	2	3	153.7	24	↓	-0.46	⇨	0.06
Q5FVQ9	Tbce	Tubulin-specific chaperone E OS=Rattus norvegicus OX=10116 GN=Tbce PE=2 SV=1	2	4	59	51	↓	-0.47	⇨	0.11
P62510	Esrrg	Estrogen-related receptor gamma OS=Rattus norvegicus OX=10116 GN=Esrrg PE=2 SV=1	1	3	51.3	0	↓	-0.47	⇨	-0.02
Q5M823	Nudcd2	NudC domain-containing protein 2 OS=Rattus norvegicus OX=10116 GN=Nudcd2 PE=2 SV=1	1	1	17.7	0	↓	-0.47	⇨	0.15
P83829	Ift81	Intraflagellar transport protein 81 homolog OS=Rattus norvegicus OX=10116 GN=Ift81 PE=2	1	1	79	0	↓	-0.47	⇨	-0.03
P37199	Nup155	Nuclear pore complex protein Nup155 OS=Rattus norvegicus OX=10116 GN=Nup155 PE=1 S\	6	12	154.9	90	↓	-0.49	⇨	0.11
F1LTR1	Wdr26	WD repeat-containing protein 26 OS=Rattus norvegicus OX=10116 GN=Wdr26 PE=3 SV=2	1	3	58.5	92	↓	-0.49	↓	-0.43
Q8CG07	Wrnip1	ATPase WRNIP1 OS=Rattus norvegicus OX=10116 GN=Wrnip1 PE=1 SV=1	1	2	71.9	24	↓	-0.49	⇨	0.02
P14141	Ca3	Carbonic anhydrase 3 OS=Rattus norvegicus OX=10116 GN=Ca3 PE=1 SV=3	10	68	29.4	1066	↓	-0.49	↓	-3.3
P11654	Nup210	Nuclear pore membrane glycoprotein 210 OS=Rattus norvegicus OX=10116 GN=Nup210 PE=	1	1	204	0	↓	-0.5	⇨	0.04
P70478	Apc	Adenomatous polyposis coli protein OS=Rattus norvegicus OX=10116 GN=Apc PE=1 SV=1	1	1	310.3	0	↓	-0.5	↓	-0.65
B2GV38	Ubl4a	Ubiquitin-like protein 4A OS=Rattus norvegicus OX=10116 GN=Ubl4a PE=2 SV=1	1	1	17.8	31	↓	-0.5	⇨	-0.01
P80254	Ddt	D-dopachrome decarboxylase OS=Rattus norvegicus OX=10116 GN=Ddt PE=1 SV=3	2	3	13.1	29	↓	-0.51	⇨	0.05
Q99P55	Sgpp1	Sphingosine-1-phosphate phosphatase 1 OS=Rattus norvegicus OX=10116 GN=Sgpp1 PE=1	1	1	47.6	0	↓	-0.51	⇨	0.32
Q68FX9	Sirt5	NAD-dependent protein deacylase sirtuin-5, mitochondrial OS=Rattus norvegicus OX=10116	1	1	34.1	0	↓	-0.51	⇨	0
O55000	Ppp1r10	Serine/threonine-protein phosphatase 1 regulatory subunit 10 OS=Rattus norvegicus OX=10	1	1	92.8	22	↓	-0.52	⇨	-0.08
P20717	Padi2	Protein-arginine deiminase type-2 OS=Rattus norvegicus OX=10116 GN=Padi2 PE=1 SV=1	1	1	75.3	22	↓	-0.52	⇨	-0.04

Q63072	Bst1	ADP-ribosyl cyclase/cyclic ADP-ribose hydrolase 2 OS=Rattus norvegicus OX=10116 GN=Bst1	2	3	35.1	37	↓	-0.53	→	-0.24
O08700	Vps45	Vacuolar protein sorting-associated protein 45 OS=Rattus norvegicus OX=10116 GN=Vps45 P	6	12	64.9	96	↓	-0.54	→	-0.16
Q5RKI3	Poll	DNA polymerase lambda OS=Rattus norvegicus OX=10116 GN=Poll PE=2 SV=1	1	5	62.4	33	↓	-0.59	→	0.17
P70475	Myt1l	Myelin transcription factor 1-like protein OS=Rattus norvegicus OX=10116 GN=Myt1l PE=1 S	1	1	132.8	25	↓	-0.59	→	-0.09
O08949	Gtf2a1	Transcription initiation factor IIA subunit 1 OS=Rattus norvegicus OX=10116 GN=Gtf2a1 PE=1	1	1	41.5	22	↓	-0.62	→	0.08
O35263	Pafah1b3	Platelet-activating factor acetylhydrolase IB subunit gamma OS=Rattus norvegicus OX=1011	2	4	25.8	52	↓	-0.62	→	0.04
P47967	Lgals5	Galectin-5 OS=Rattus norvegicus OX=10116 GN=Lgals5 PE=1 SV=2	4	23	16.2	350	↓	-0.68	→	0.17
P02634	S100g	Protein S100-G OS=Rattus norvegicus OX=10116 GN=S100g PE=1 SV=3	2	3	9	122	↓	-0.69	↑	0.83
D3ZFJ3	Sh3bp1	SH3 domain-binding protein 1 OS=Rattus norvegicus OX=10116 GN=Sh3bp1 PE=1 SV=1	3	5	74.8	43	↓	-0.69	→	0.33
Q8VHK0	Acot8	Acyl-coenzyme A thioesterase 8 OS=Rattus norvegicus OX=10116 GN=Acot8 PE=1 SV=1	1	1	36	21	↓	-0.83	→	0.27
Q5PQJ7	Tbcel	Tubulin-specific chaperone cofactor E-like protein OS=Rattus norvegicus OX=10116 GN=Tbce	1	2	48	21	↓	-0.89	→	-0.06
Q5RJZ1	Rtel1	Regulator of telomere elongation helicase 1 OS=Rattus norvegicus OX=10116 GN=Rtel1 PE=2	1	3	141.7	19	↓	-0.91	→	-0.25
Q9JK71	Magi3	Membrane-associated guanylate kinase, WW and PDZ domain-containing protein 3 OS=Rat	7	15	160.5	188	↓	-0.98	→	-0.01
Q923V4	Fbxo6	F-box only protein 6 OS=Rattus norvegicus OX=10116 GN=Fbxo6 PE=1 SV=1	5	11	32.8	93	↓	-1.07	→	0.13

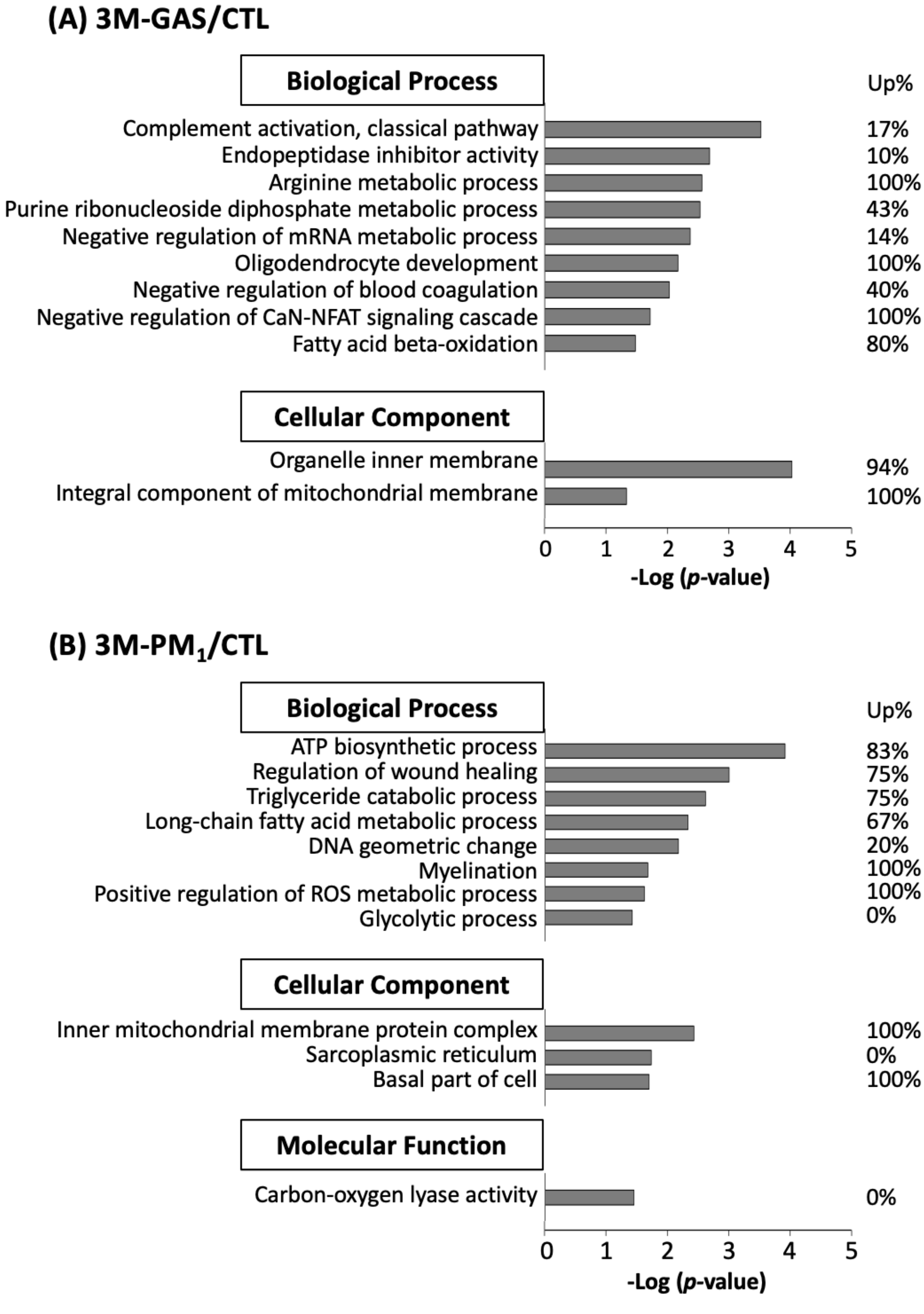

Additional file 4: Figure S1. The Gene Ontology analysis of DEPs in subchronic exposures groups. The enriched biological process, molecular function and cellular component in (**A**) 3M-GAS group and (**B**) 3M-PM_1 group with comparison to 3M-CTL group. The percentage of up-regulated proteins involved in each enriched term is indicated next to bar.

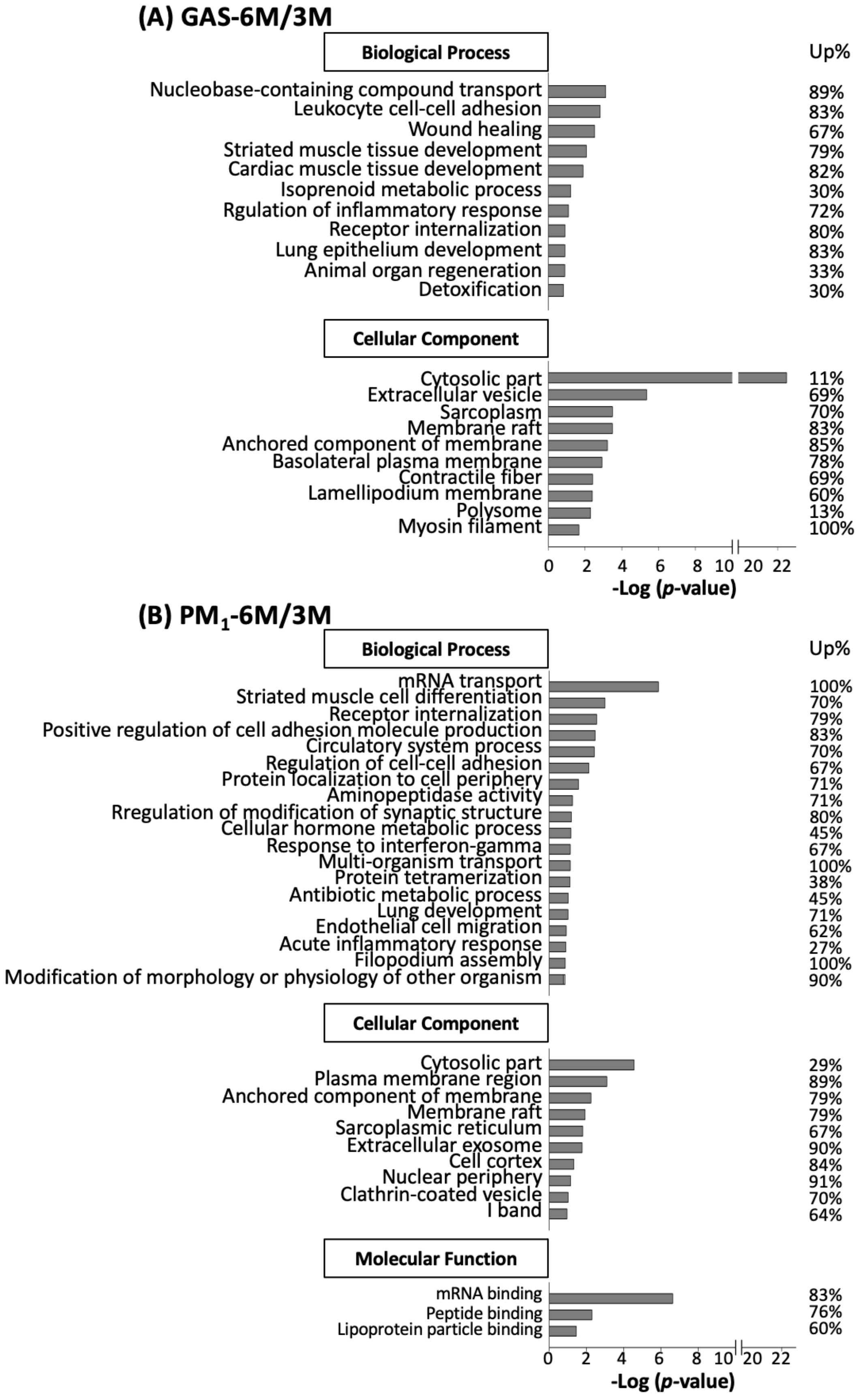
(A) GAS-6M/3M
Biological Process
Up%
Nucleobase-containing compound transport 89%
Leukocyte cell-cell adhesion 83%
Wound healing 67%
Striated muscle tissue development 79%
Cardiac muscle tissue development 82%
Isoprenoid metabolic process 30%
Rgulation of inflammatory response 72%
Receptor internalization 80%
Lung epithelium development 83%
Animal organ regeneration 33%
Detoxification 30%
Cellular Component
Cytosolic part 11%
Extracellular vesicle 69%
Sarcoplasm 70%
Membrane raft 83%
Anchored component of membrane 85%
Basolateral plasma membrane 78%
Contractile fiber 69%
Lamellipodium membrane 60%
Polysome 13%
Myosin filament 100%
0 2 4 6 8 10 20 22
-Log (p-value)
(B) PM1-6M/3M
Biological Process
Up%
mRNA transport 100%
Striated muscle cell differentiation 70%
Receptor internalization 79%
Positive regulation of cell adhesion molecule production 83%
Circulatory system process 70%
Regulation of cell-cell adhesion 67%
Protein localization to cell periphery 71%
Aminopeptidase activity 71%
Rregulation of modification of synaptic structure 80%
Cellular hormone metabolic process 45%
Response to interferon-gamma 67%
Multi-organism transport 100%
Protein tetramerization 38%
Antibiotic metabolic process 45%
Lung development 71%
Endothelial cell migration 62%
Acute inflammatory response 27%
Filopodium assembly 100%
Modification of morphology or physiology of other organism 90%
Cellular Component
Cytosolic part 29%
Plasma membrane region 89%
Anchored component of membrane 79%
Membrane raft 79%
Sarcoplasmic reticulum 67%
Extracellular exosome 90%
Cell cortex 84%
Nuclear periphery 91%
Clathrin-coated vesicle 70%
I band 64%
Molecular Function
mRNA binding 83%
Peptide binding 76%
Lipoprotein particle binding 60%
0 2 4 6 8 10 20 22
-Log (p-value)

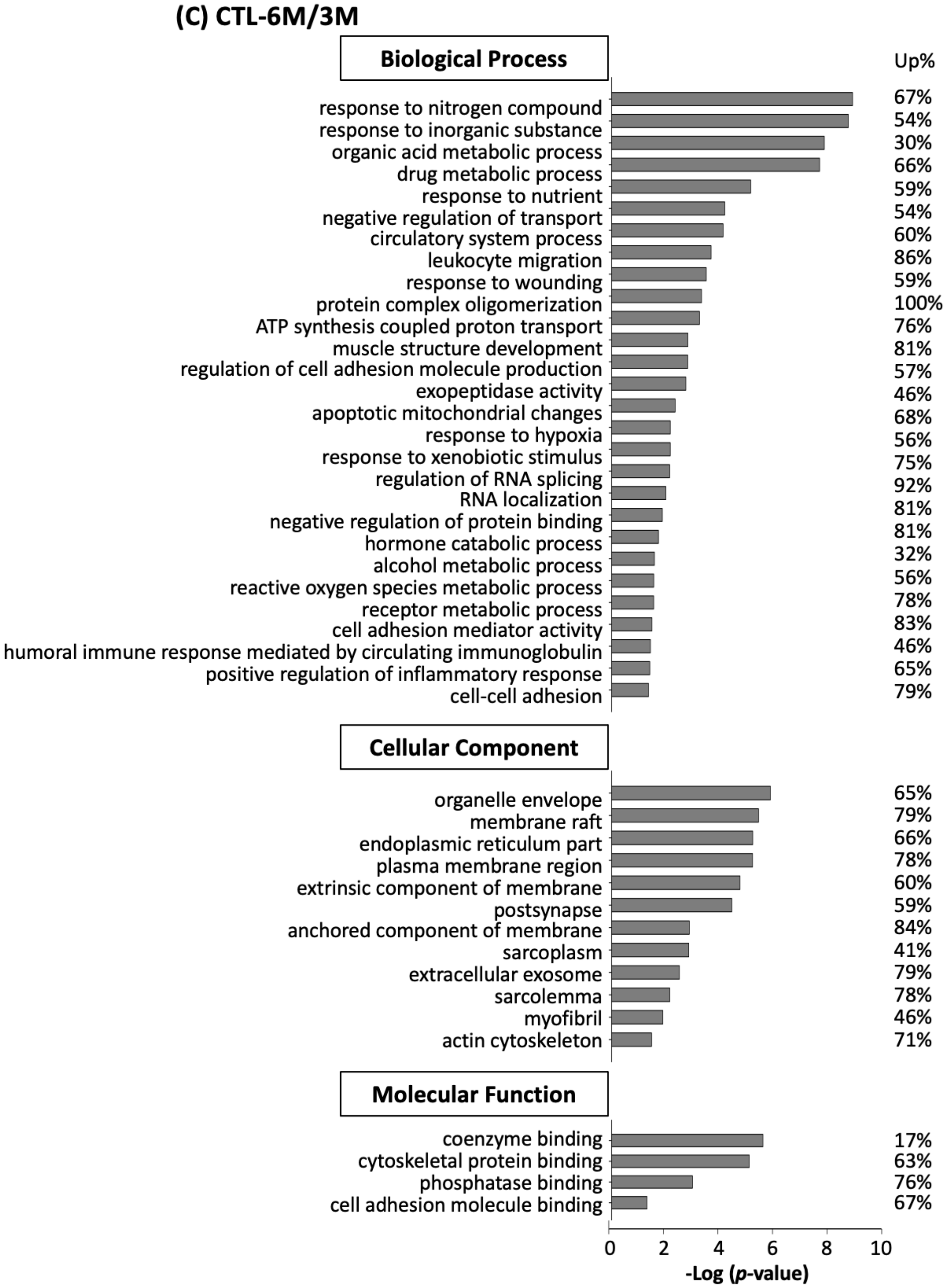

Additional file 5: Figure S2. The Gene Ontology analysis of DEPs in progressive exposure to GAS, PM_1, and CTL groups. The enriched biological process, molecular function and cellular component in (**A**) GAS group, (**B**) PM_1 group, and **(C)** CTL group. The percentage of up-regulated proteins involved in each enriched term is indicated next to bar.

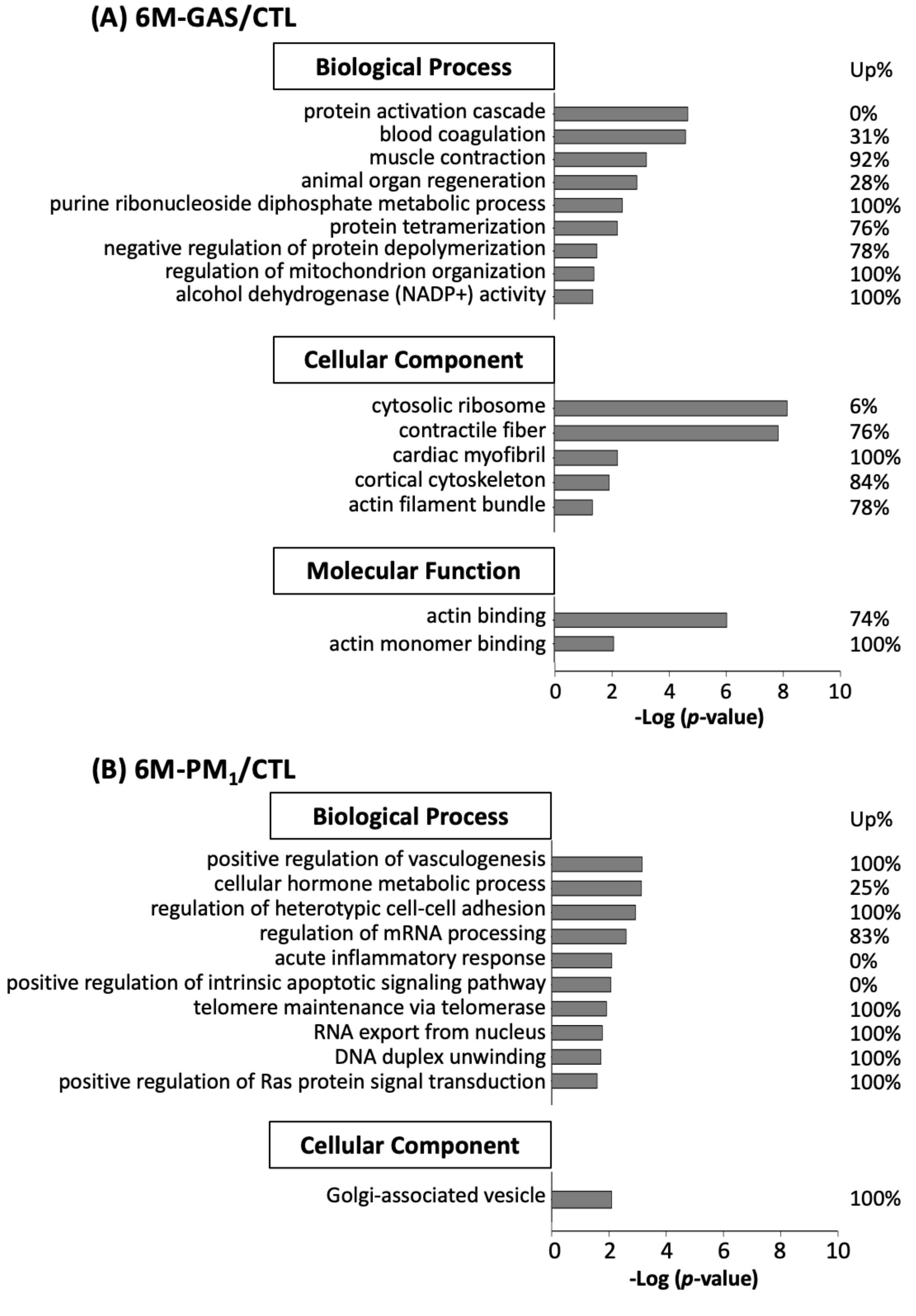

Additional file 6: Figure S3. The Gene Ontology analysis of DEPs in chronic exposure groups. The enriched biological process, molecular function and cellular component in (**A**) 6M-GAS group and (**B**) 6M-PM_1 group with comparison to 6M-CTL group. The percentage of up-regulated proteins involved in each enriched term is indicated next to bar.

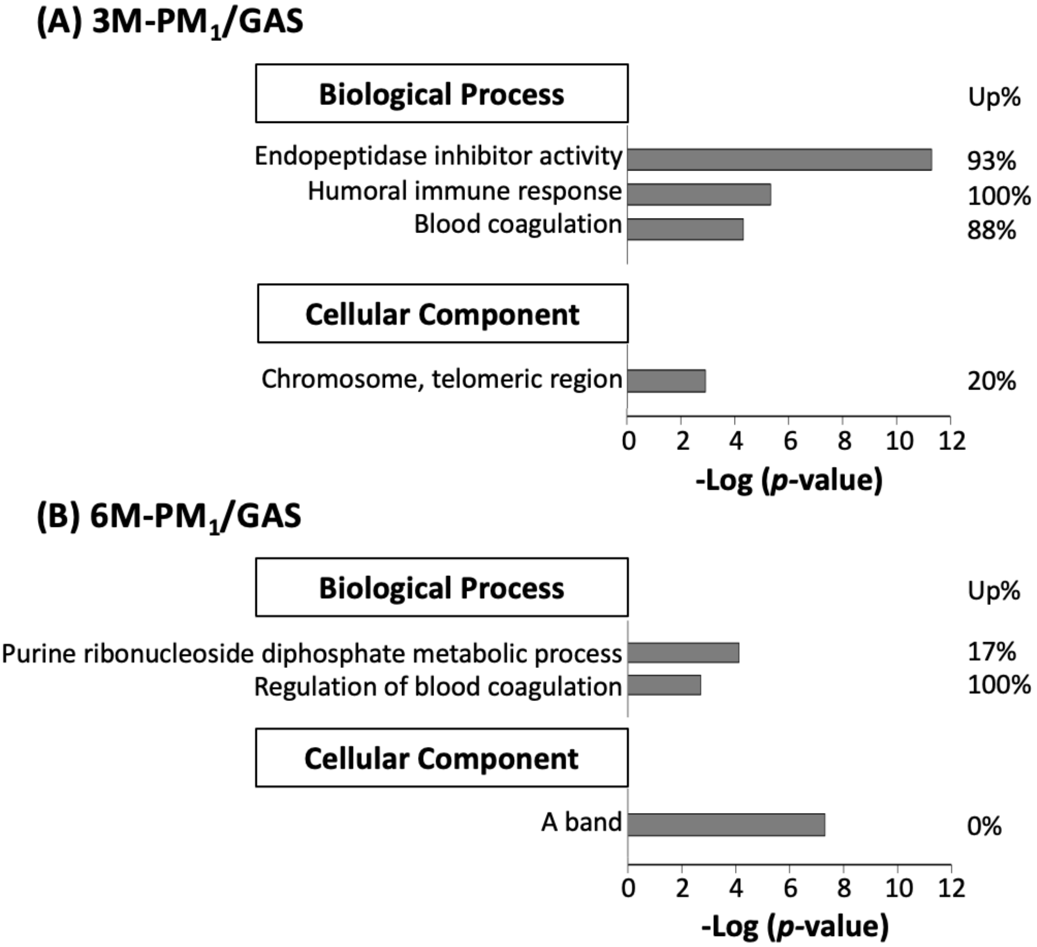

Additional file 7: Figure S4. The Gene Ontology analysis of DEPs specifically regulated by particles. The enriched biological process and molecular function specifically regulated by particles under (**A**) 3-month and (**B**) 6-month exposures. The percentage of up-regulated proteins involved in each enriched term is indicated next to bar.

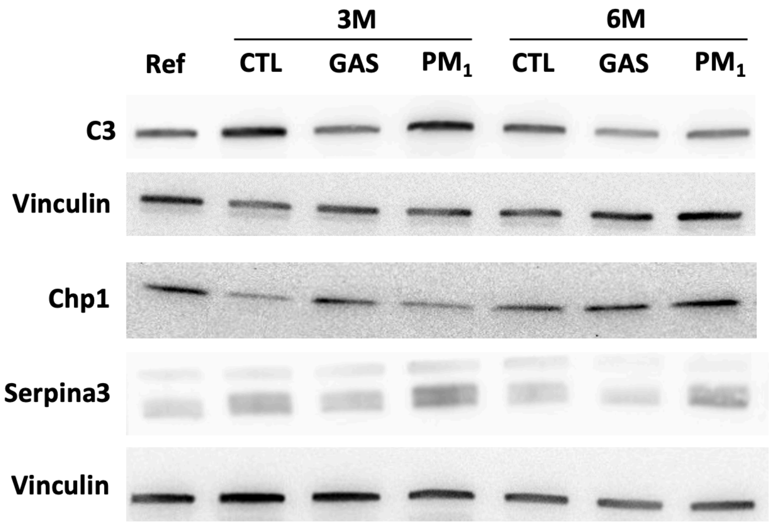

Additional file 8: Figure S5. Western blot validation. The representative Western blot results for expression levels of C3, Serpina3 and Chp1 in rat lung tissues. A reference sample was run in every analysis for normalization. Vinculin is served as loading control.
