# Supplementary figures and images for: Prolonged exposure to traffic-related particulate matter and gaseous pollutants implicate distinct molecular mechanisms of lung injury in rats
Source: Part Fibre Toxicol. 2021 Jun 25;18:24. doi: 10.1186/s12989-021-00417-y (PMC8235648; doi:10.1186/s12989-021-00417-y)

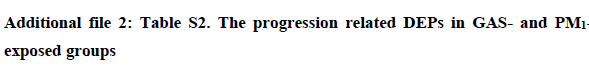

Supplement: Supplementary file 2 — Additional file 2: Table S2. The progression related DEPs in GAS- and PM1exposed groups [file 12989_2021_417_MOESM2_ESM.docx]

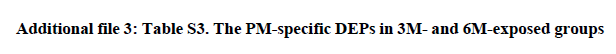

Supplement: Supplementary file 3 — Additional file 3: Table S3. The PM-specific DEPs in 3 M- and 6 M-exposed groups [file 12989_2021_417_MOESM3_ESM.docx]

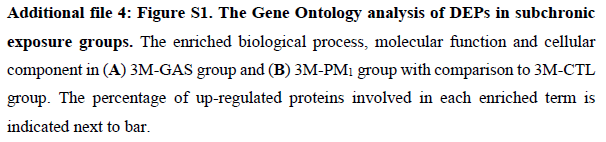

Supplement: Supplementary file 4 — Additional file 4: Figure S1. The Gene Ontology analysis of DEPs in subchronic exposure groups. The enriched biological process, molecular function and cellular component in (A) 3 M-GAS group and (B) 3 M-PM1 group with comparison to 3 M-CTL group. The percentage of up-regulated proteins involved in each enriched term is indicated next to bar. [file 12989_2021_417_MOESM4_ESM.docx]

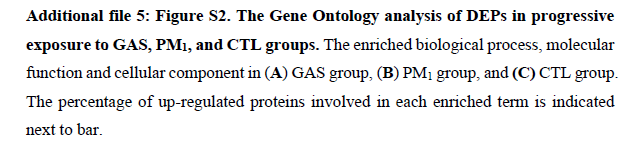

Supplement: Supplementary file 5 — Additional file 5: Figure S2. The Gene Ontology analysis of DEPs in progressive exposure to GAS, PM1, and CTL groups. The enriched biological process, molecular function and cellular component in (A) GAS group, (B) PM1 group, and (C) CTL group. The percentage of up-regulated proteins involved in each enriched term is indicated next to bar. [file 12989_2021_417_MOESM5_ESM.docx]

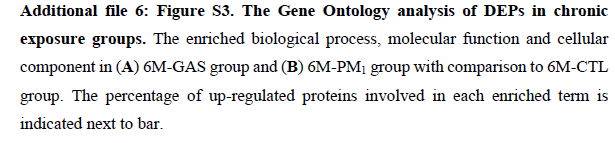

Supplement: Supplementary file 6 — Additional file 6: Figure S3. The Gene Ontology analysis of DEPs in chronic exposure groups. The enriched biological process, molecular function and cellular component in (A) 6 M-GAS group and (B) 6 M-PM1 group with comparison to 6 M-CTL group. The percentage of up-regulated proteins involved in each enriched term is indicated next to bar. [file 12989_2021_417_MOESM6_ESM.docx]

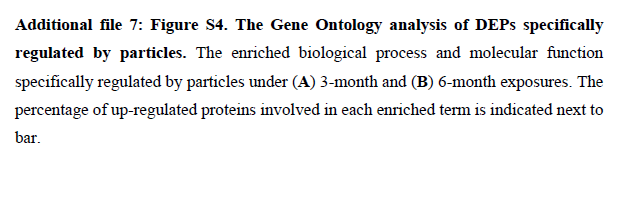

Supplement: Supplementary file 7 — Additional file 7: Figure S4. The Gene Ontology analysis of DEPs specifically regulated by particles. The enriched biological process and molecular function specifically regulated by particles under (A) 3-month and (B) 6-month exposures. The percentage of up-regulated proteins involved in each enriched term is indicated next to bar. [file 12989_2021_417_MOESM7_ESM.docx]

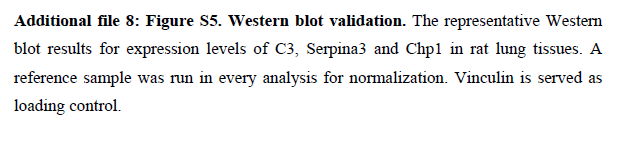

Supplement: Supplementary file 8 — Additional file 8: Figure S5. Western blot validation. The representative Western blot results for expression levels of C3, Serpina3 and Chp1 in rat lung tissues. A reference sample was run in every analysis for normalization. Vinculin is served as loading control. [file 12989_2021_417_MOESM8_ESM.docx]
